# Safety and Immunogenicity of Heterologous Prime-Boost Immunisation with *Plasmodium falciparum* Malaria Candidate Vaccines, ChAd63 ME-TRAP and MVA ME-TRAP, in Healthy Gambian and Kenyan Adults

**DOI:** 10.1371/journal.pone.0057726

**Published:** 2013-03-19

**Authors:** Caroline Ogwang, Muhammed Afolabi, Domtila Kimani, Ya Jankey Jagne, Susanne H. Sheehy, Carly M. Bliss, Christopher J. A. Duncan, Katharine A. Collins, Miguel A. Garcia Knight, Eva Kimani, Nicholas A. Anagnostou, Eleanor Berrie, Sarah Moyle, Sarah C. Gilbert, Alexandra J. Spencer, Peninah Soipei, Jenny Mueller, Joseph Okebe, Stefano Colloca, Riccardo Cortese, Nicola K. Viebig, Rachel Roberts, Katherine Gantlett, Alison M. Lawrie, Alfredo Nicosia, Egeruan B. Imoukhuede, Philip Bejon, Britta C. Urban, Katie L. Flanagan, Katie J. Ewer, Roma Chilengi, Adrian V. S. Hill, Kalifa Bojang

**Affiliations:** 1 Kenya Medical Research Institute, Centre for Geographical Medical Research (Coast), Kilifi, Kenya; 2 Medical Research Council Unit, Fajara, The Gambia; 3 Centre for Clinical Vaccinology and Tropical Medicine, The Jenner Institute, Churchill Hospital, Oxford, United Kingdom; 4 The Jenner Institute Laboratories, University of Oxford, Old Road Campus Research Building, Oxford, United Kingdom; 5 Clinical Biomanufacturing Facility, University of Oxford, Churchill Hospital, Oxford, United Kingdom; 6 Okairòs AG, Rome, Italy; 7 CEINGE, Naples, Italy; 8 European Vaccine Initiative, Heidelberg, Germany; 9 Department of Molecular Medicine and Medical Biotechnology, University Federico II Naples, Naples, Italy; 10 Liverpool School of Tropical Medicine, Liverpool, United Kingdom; University of California Los Angeles, United States of America

## Abstract

**Background:**

Heterologous prime boost immunization with chimpanzee adenovirus 63 (ChAd63) and Modified vaccinia Virus Ankara (MVA) vectored vaccines is a strategy recently shown to be capable of inducing strong cell mediated responses against several antigens from the malaria parasite. ChAd63-MVA expressing the *Plasmodium falciparum* pre-erythrocytic antigen ME-TRAP (multiple epitope string with thrombospondin-related adhesion protein) is a leading malaria vaccine candidate, capable of inducing sterile protection in malaria naïve adults following controlled human malaria infection (CHMI).

**Methodology:**

We conducted two Phase Ib dose escalation clinical trials assessing the safety and immunogenicity of ChAd63-MVA ME-TRAP in 46 healthy malaria exposed adults in two African countries with similar malaria transmission patterns.

**Results:**

ChAd63-MVA ME-TRAP was shown to be safe and immunogenic, inducing high-level T cell responses (median >1300 SFU/million PBMC).

**Conclusions:**

ChAd63-MVA ME-TRAP is a safe and highly immunogenic vaccine regimen in adults with prior exposure to malaria. Further clinical trials to assess safety and immunogenicity in children and infants and protective efficacy in the field are now warranted.

**Trial Registration:**

Pactr.org PACTR2010020001771828

Pactr.org PACTR201008000221638

ClinicalTrials.gov NCT01373879 NCT01373879

ClinicalTrials.gov NCT01379430 NCT01379430

## Introduction

Malaria caused by *Plasmodium falciparum* remains a leading cause of childhood morbidity and mortality, predominantly in Africa, in spite of the implementation of extensive control measures [1], [2]. An effective vaccine remains a key objective if disease transmission and severity is to be substantially reduced [3]. The most advanced malaria vaccine in development, the protein-adjuvant vaccine RTS,S/AS01 targeting the pre-erythrocytic stage of infection [4], is currently in phase III clinical trials and has been shown to confer partial protection over the 12 months following immunization [5], [6]. Whilst notable as the most efficacious malaria vaccine to date there remains a considerable need to improve on its limited clinical efficacy [7], either through modifications to the RTS,S vaccine or by developing vaccine strategies that combine multiple antigens or vaccine types [8].

Analysis of the immunological correlates of immunity induced by the RTS,S vaccine in both phase IIa sporozoite challenge studies [9], [10] and a trial in Mozambique [11] provide evidence that very high levels of antibodies to circumsporozoite protein (CS) correlate with protection in humans [12]. However, this correlation is relatively weak. It is unlikely that there is a component of direct T cell mediated protection induced by the vaccine as the magnitude of the CD4+ T cell response measured after vaccination is modest (approximately 150 SFU /million PBMCs on ELIspot) and no CD8+ T cells are induced [13].

Increasing data from animal models, fieldwork and inoculation of volunteers with irradiated sporozoites support an important role for CD8+ T cells in mediating pre-erythrocytic immunity, even in the absence of antibodies [14]. Whilst pre-clinical studies demonstrate a clear correlation between CD8+ T cells and protection [15]–[19], clinical vaccine studies have been hampered by the limited ability of existing subunit vaccine strategies, namely adjuvanted protein constructs, to induce high enough numbers of antigen specific CD8+ T cells to confer protection [20].

The Jenner Institute has been working to develop a T cell inducing pre-erythrocytic *P. falciparum* malaria vaccine using the sporozoite and liver stage antigen ME-TRAP. This antigen contains a fusion protein of multiple epitopes (ME; a string of 20 epitopes, mainly CD8+ T cell epitopes from pre-erythrocytic antigens) and the *P. falciparum* pre-erythrocytic antigen, thrombospondin-related adhesion protein (TRAP) [21], [22].

Multiple vectors for this antigen have been clinically tested including DNA, fowl pox (FP) and the orthopox virus modified vaccinia virus Ankara (MVA) [23]–[34]. Whilst some of these vaccines are capable of inducing partial protection following controlled human malaria infection (CHMI) in malaria naive volunteers [21], this did not translate into efficacy in field studies [32], [33] likely due to a substantial reduction in T cell immunogenicity observed in malaria exposed vaccinees compared to UK volunteers [32].

Most recently, heterologous prime boost immunization with chimpanzee adenovirus 63 (ChAd63) followed by MVA, both expressing ME-TRAP, has been shown to be the most immunogenic vaccine regimen to date, inducing more than 2000 IFNγ producing T cells post MVA boost in malaria naïve volunteers [35]. This translated into significant clinical efficacy following CHMI administered by mosquito bite with both sterile and partial protection observed for multiple vaccinees in a phase IIa trial in the UK in which strong CD8+ T cell responses were induced (*Ewer et al.* submitted).

The safety, immunogenicity and efficacy of malaria vaccines may be affected by the intensity and pattern of local malaria transmission which determine pre-existing natural immunity to malaria and the potential natural ‘boosting’ of the vaccine induced immune responses [29]. It is therefore useful to assess the safety and immunogenicity of candidate vaccines in malaria exposed adults prior to age de-escalation and administration to children and infants, the target population. Here we present the safety and immunogenicity results of two Phase Ib clinical trials of ChAd63-MVA ME-TRAP in malaria exposed male adult volunteers, under taken at two sites with similar malaria transmission patterns in West and East Africa. Both studies included a dose escalation of ChAd63 ME-TRAP and one site (Kenya) compared the safety and immunogenicity of MVA ME-TRAP administered by intramuscular and intradermal routes.

## Methods

The protocols for these trials and supporting CONSORT checklist are available as supporting information; see Protocol S1, Protocol S2 and Checklist S1.

### Objective

The objective of the studies was to assess the safety and immunogenicity of ChAd63 ME-TRAP and MVA ME-TRAP administered in a heterologous prime boost regimen to healthy malaria-exposed adults.

### Study settings

The first trial (Trial A) was conducted at the Medical Research Council (MRC) Unit field site located within Sukuta Health Centre in Kombo North district of The Gambia, West Africa. Sukuta village has an estimated population of 17,000 (2003 census). The climate is typical of sub-Saharan Africa with a long dry season lasting from December–June followed by a relatively short rainy season from July–November when the majority of *P. falciparum* malaria transmission occurs [36].

The second trial (Trial B) was conducted in Vipingo, Kilifi County, Kenya, East Africa. Participants were recruited from the Rea Vipingo Sisal Plantation Estates in Kilifi which has over 1000 employees and a land area of 3,950 hectares. In Kilifi, there are two seasons of high transmission of *P. falciparum* malaria coinciding with the long monsoon rains (April to June) and the short rains (October to December) [37].

Recent studies have reported a decline in malaria transmission in both sites [38], [39] but a surge was recorded during the period of vaccinations in the Sukuta site (*M. Afolabi personal communication*).

### Participants

Healthy males aged 18–50 years were invited to participate in the studies. There was no selection of participants on the basis of pre-existing neutralizing antibodies (NAb) to the ChAd63 vector prior to enrolment. Volunteers were considered eligible if they were consenting adult males aged 18–50 years in good health who were likely to remain resident in the study area for the study duration. Exclusion criteria included any evidence of any acute or chronic illness or hematological, renal or hepatic pathology. Specific exclusion criteria included; prior receipt of an investigational malaria vaccine, recent or planned use of any investigational drug, vaccine, immunoglobulin or any blood product, confirmed or suspected immunodeficiency, history of surgical splenectomy, concurrent participation in another clinical trial or within 3 months of this study (see Protocol S1 (Trial A: The Gambia), Protocol S2 (Trial B: Kenya) for the full list of inclusion and exclusion criteria). Blood positivity for *P. falciparum* at screening was not an exclusion criterion.

### Study Design

**Figure 1:**
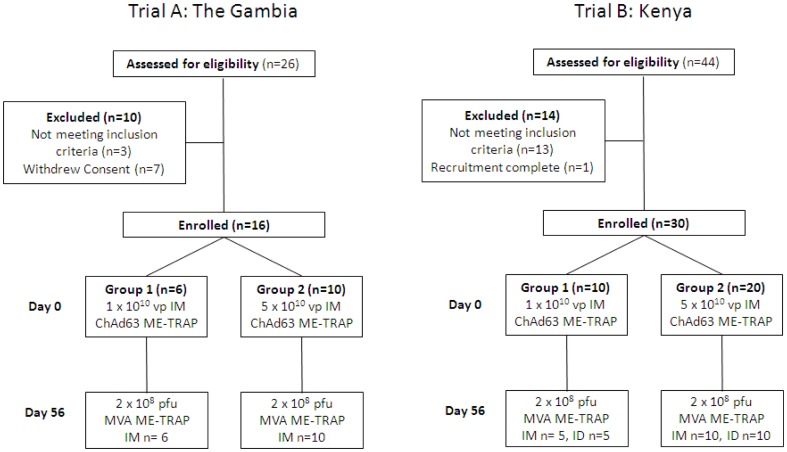
Clinical Trial Designs. Trial A = Phase Ib clinical trial in The Gambia, West Africa. Trial B = Phase Ib clinical trial in Kilifi, Kenya, East Africa. IM = intramuscular administration. ID = intradermal administration. In Trial A, 10 volunteers were excluded following screening for the following reasons: severe thrombocytopenia, severe proteinuria, spastic deformity of arm and withdrawal of consent (seven individuals). In Trial B, 14 volunteers were excluded following screening for the following reasons: hypertension (two individuals), positive serology for HIV (two individuals), positive Hepatitis B surface antigen (four individuals), participation in a previous malaria vaccine trial (2 individuals), peptic ulcer disease, allergic disease, recruitment complete (one participant).

We conducted two Phase Ib open-label, dose-escalation malaria vaccine trials (Figure 1). Both clinical trials evaluated low (1×10^10^ vp) and high dose (5×10^10^ vp) ChAd63 ME-TRAP. Trial B also compared intramuscular and intradermal routes of administration of 2×10^8^ pfu MVA ME-TRAP. The same lot of each vaccine was used in both trials. The trials were conducted independently however the same Data and Safety Monitoring Board (DSMB) was used for each trial. The clinical trial protocols and supporting CONSORT checklist are available as Supplementary Information; see Protocol S1 (Trial A: The Gambia), Protocol S2 (Trial B: Kenya), and Checklist S1.

In Trial A, eligible participants were allocated to receive either ChAd63 ME-TRAP 1×10^10^ viral particles (vp) (group 1; n = 6) or ChAd63 ME-TRAP 5×10^10^ vp (group 2; n = 10) administered intramuscularly in the deltoid. All participants were subsequently vaccinated in the opposite arm 56 days later with 2×10^8^ plaque forming units (pfu) MVA ME-TRAP administered intramuscularly. The first participant in group 1 to receive ChAd63 ME-TRAP 1×10^10^ vp was vaccinated in isolation. 48 hours later, two further participants were enrolled in group 1. Prior to dose escalation of ChAd63 ME-TRAP from 1×10^10^ vp to 5×10^10^ vp, safety data from group 1 up to 14 days post ChAd63 ME-TRAP was reviewed by the DSMB. There was a protocol-required interval of at least 14 days between immunization of groups 1 and 2. Details of clinical follow-up and safety monitoring are given in Protocol S1 and S2.

In Trial B, eligible participants were allocated to receive either ChAd63 ME-TRAP 1×10^10^ viral particles (vp) (group 1; n = 10) or ChAd63 ME-TRAP 5×10^10^ vp (group 2; n = 20) administered intramuscularly in the deltoid. All participants were subsequently vaccinated in the opposite arm 56 days later with 2×10^8^ plaque forming units (pfu) MVA ME-TRAP. Participants in each group were randomised 1∶1 to receive MVA ME-TRAP administered intramuscularly (IM) or intradermally (ID). The first 3 participants in group 1 were vaccinated with ChAd63 ME-TRAP 1×10^10^ vp 7 days ahead of the remaining 7 participants in this group. There was an 8 day interval between enrolment of group 1 and group 2. Details of clinical follow-up and safety monitoring are given in Protocol S1 and S2.

For both trials, a time window ranging between 1 and 28 days depending on the visit was allowed for vaccination and follow-up visits. Throughout the paper, study day refers to the nominal time point for a group and not the actual day of sampling.

### Randomisation in Trial B

30 participants were systematically allocated to receive either 1×10^10^ vp ChAd63 ME - TRAP or 5×10^10^ vp dose ChAd63 ME in a ratio of 1∶2. 8 weeks later participants were randomised 1∶1 to receive 2×10^8^ pfu MVA ME-TRAP administered intramuscularly (IM) or intradermally (ID). The randomization sequence was generated by an independent statistician using STATA programme. Group allocations were kept in sealed opaque envelopes stored in a locked cabinet by the study coordinator who gave them to the research nurses on day of vaccination. Participants and clinical study staff were un-blinded to group allocation, however, field workers were blinded to group allocation.

### Sample size

These were observational and descriptive studies to assess the safety and immunogenicity of ChAd63 ME-TRAP and MVA ME-TRAP in malaria exposed adults. The sample sizes were chosen to allow estimation of the magnitude of the primary outcome measures, especially of serious adverse events (AEs) rather than assessment of statistically significant differences between groups.

### Ethical & Regulatory Approval

The clinical trial protocols and associated documents were approved by Gambia Government/MRC Joint Ethics Committee for Trial A and The Kenya Medical Research Institute National Ethics Review Committee for Trial B. Documents for both clinical trials were reviewed and approved by the Oxford Tropical Research Ethics Committee (OXTREC). Regulatory approval was given by the Medicines Board of The Gambia for Trial A and The Pharmacy and Poisons Board of Kenya for Trial B. All participants gave documented informed consent prior to any study procedure being undertaken. The study was conducted according to the principles of the Declaration of Helsinki (2008) and the International Conference on Harmonization (ICH) Good Clinical Practice (GCP) guidelines. An independent DSMB and local safety monitors provided safety oversight and GCP compliance was independently monitored by an external organization at both trial sites (Appledown Clinical Research Ltd, Great Missenden, UK).

### ChAd63 ME-TRAP and MVA ME-TRAP Vaccines

Generation of the recombinant vectors has been previously described [40], [41]. Vaccines were manufactured under Good Manufacturing Practice (GMP) conditions by the Clinical Biomanufacturing Facility, University of Oxford (ChAd63 ME-TRAP) and IDT Biologika GmbH, Dessau, Germany (MVA ME-TRAP). Briefly, ChAd63 ME-TRAP was grown in suspension HEK293 cells and purified by caesium chloride density-gradient centrifugation. MVA ME-TRAP was generated in chicken embryo fibroblasts (CEFs) and purified by sucrose density-gradient centrifugation. Each vaccine lot underwent comprehensive quality control analysis to ensure that the purity, identity and integrity of the virus met pre-defined specifications. Vaccine lots were stored at the clinical site in a −70°C freezer and vaccines were temperature monitored when moved.

The antigen ME-TRAP contains a fusion protein of multiple epitopes (ME) and the *P. falciparum* pre-erythrocytic thrombospondin-related adhesion protein (TRAP). The ‘ME’ is a string of 20 epitopes, mainly CD8 T cell epitopes from *P. falciparum* pre-erythrocytic antigens, fused to the thrombospondin-related adhesion protein. The individual cytotoxic T lymphocyte (CTL) epitopes which constitute the ‘multiple epitope’ part of ME-TRAP represent six potentially protective target antigens and are included to try to broaden the immune response rate in the vaccinated population. The ME string is fused to the entire sequence of the T9/96 strain of *P. falciparum* TRAP and the ME-TRAP hybrid is a 2398 base-pair insert which encodes for a single polypeptide of 789 amino acids [21].

### Safety

In each trial participants were observed for 1 hour post each immunization. Following each immunization participants in both trials were reviewed at home by a trained field worker and findings recorded on standardised case report forms. Local and systemic vaccine reactogenicity was evaluated and graded for severity, outcome and association to vaccination as per the criteria outlined in Tables 1, 2, 3, 4, 5. Local solicited reactions were; pain, discoloration, swelling, warmth, pruritus, scaling or blistering at the injection site. Systemic solicited symptoms were; fever (axillary temperature>37.5°C), feverishness, malaise, arthralgia, headache, myalgia and nausea or vomiting. Unsolicited symptoms that occurred within 30 days of each immunization were assessed, recorded and their relationship to the immunization determined. Serious adverse events (SAEs) were assessed throughout the study period. First response® Rapid Diagnostic kits (Trial A) or blood film microscopy (Trial B) [42] were performed for diagnostic purposes whenever participants presented with symptoms suggestive of malaria at each trial site. In Trial A blood was sampled at all clinic visits post vaccination (days 14, 56, 63, 90 and 300 post ChAd63 ME-TRAP) and full blood count, creatinine and alanine aminotransferase (ALT) measured. In Trial B blood was sampled at all clinic visits post vaccination (days 14, 56, 63, 91 and 308 post ChAd63 ME-TRAP) and full blood count, creatinine and ALT measured.

**Table 1:** Assessment of Severity of Local AEs. Discoloration.

Grade	Diameter (mm)
0	0
1	<50
2	50–100
3	>100

**Table 2:** Assessment of Severity of Local AEs. Swelling.

Grade	Diameter (mm)
0	0
1	<20
2	20–50
3	>50

**Table 3:** Assessment of Severity of Local AEs. Pain.

Grade	Description
0	No pain at all
1	Painful to touch, no restriction in movement of arms, able to work, drive, carry heavy objects as normal
2	Painful when limb is moved (*i.e.* restriction in range of movement in arm, difficulty in carrying objects)
3	Severe pain at rest (*i.e.* unable to use arm due to pain.)

**Table 4:** Assessment of Severity of Systemic AEs.

Scale	Description	Definition
0		Absence of the indicated symptom
1	Mild	Awareness of a symptom but the symptom is easily tolerated
2	Moderate	Discomfort enough to cause interference with usual activity
3	Severe	Incapacitating; unable to perform usual activities; requires absenteeism or bed rest

**Table 5:** Assessment of Relationship of AE to Immunization.

**0**	**No Relationship**	No temporal relationship to study product ***and*** Alternate aetiology (clinical state, environmental or other interventions); ***and*** Does not follow known pattern of response to study product
**1**	**Possible**	Reasonable temporal relationship to study product; *or* Event not readily produced by clinical state, environmental ***or*** other interventions; ***or*** Similar pattern of response to that seen with other vaccines
**2**	**Probable**	Reasonable temporal relationship to study product; ***and*** Event not readily produced by clinical state, environment, ***or*** other interventions *or* Known pattern of response seen with other vaccines
**3**	**Definite**	Reasonable temporal relationship to study product; ***and*** Event not readily produced by clinical state, environment, ***or*** other interventions; ***and*** Known pattern of response seen with other vaccines

### Peripheral Blood Mononuclear Cell (PBMC) and Serum Preparation

Blood samples were collected into lithium or sodium heparin-treated vacutainer blood collection tubes (Becton Dickinson, UK). PBMC were isolated and used within 6 hours in fresh assays as previously described [43]. Excess cells were frozen in foetal calf serum (FCS) containing 10% dimethyl sulfoxide (DMSO) and stored in liquid nitrogen. For serum preparation, untreated blood samples were stored at 4°C and then the clotted blood was centrifuged for 5 min (1000 *xg*). Serum was stored at −80°C.

### Peptides for T cell Assays

Peptides were purchased from NEO Peptide (Cambridge, MA, USA). The peptides, 20 amino acids (aa) in length and overlapping by 10 aa covered the entire ME-TRAP insert present in the viral vectored vaccines. Peptides were also synthesised for the sequence of TRAP from the 3D7 strain. Peptides were reconstituted in 100% DMSO at 50–200 mg/mL and combined into various pools for ELISPOT and flow cytometry assays. Peptides are listed in Table S1.

### *Ex-vivo* interferon-γ (IFN-γ) ELISPOT

The kinetics and magnitude of the T cell response to ME-TRAP were assessed over time by *ex-vivo* IFN-γ ELISPOT assays performed on blood samples taken at each clinic review (days 14, 56, 63, 90 and 300 post ChAd63 ME-TRAP in Trial A and days 14, 56, 63, 91 and 308 post ChAd63 ME-TRAP in Trial B). *Ex-vivo* IFN-γ ELISPOT assays were performed with an 18–20 hour stimulation of PBMC with peptides pools containing up to 10 peptides per pool, including peptides representing the T9/96 and 3D7 strains. Fresh PBMC were used in all ELISPOT assays using a previously described protocol, except that 50 µL/well ME-TRAP peptide pools (final concentration of each peptide 10 µg/mL) were added to duplicate wells, 50 µL/well R10 and DMSO control were added to negative un-stimulated wells, and 50 µL/well Staphylococcal enterotoxin B (SEB) (final concentration 0.02 µg/mL) plus phytohemagglutinin (PHA) (final concentration 10 µg/mL) was added to positive control wells. Spots were counted using an ELISPOT counter (Autoimmun Diagnostika (AID), Germany). Results are expressed as the mean of the duplicate IFN-γ spot-forming units (SFU) per million PBMC. Background responses in un-stimulated control wells were almost always less than 20 spots, and were subtracted from those measured in peptide-stimulated wells. Responses are shown as the summed response to all the ME-TRAP (T9/96) peptide pools (unless otherwise stated).

### Statistical Analysis

Data were analyzed using GraphPad Prism version 5.03 for Windows (GraphPad Software Inc., California, USA) and Stata 10.0 (Statacorp LP, Texas, USA). Geometric mean or median responses for each group are described. Significance testing of differences between two groups used the two-tailed Mann-Whitney U test or Wilcoxon signed rank test as appropriate. Correlations were analyzed using Spearman's rank correlation co-efficient (r_s_) for non-parametric data. Collated immunology data was analysed by multivariate linear regression using log-transformed ELISPOT results. A value of *P*<0.05 was considered significant.

## Results

### Study Recruitment

Recruitment for Trial A took place in the Gambia between 19^th^ May 2010 and 9^th^ June 2010. Sixteen healthy male adult participants were enrolled, immunized and followed up (Figure 1). The mean age of volunteers was 33.3 years (range 23–48 years). All participants were from the Mandinka ethnic group. Vaccinations began in June 2010 and all follow-up visits were completed by May 2011. With the exception of a participant in group 2 who was lost to follow-up after review on Day 90, all volunteers attended all visits as scheduled and completed the study.

Recruitment for Trial B took place in Kenya between 10^th^ June 2010 and 7^th^ July 2010. Thirty healthy male adult participants were enrolled, immunized and followed up (Figure 1). The mean age of volunteers was 32.5 years (range 22–50). 47% of participants were from the Mijikenda ethnic group, 27% were Luos and the remaining 26% from other ethnic groups. Vaccinations began in June 2010 and all follow-up visits were completed by May 2011. All volunteers attended all visits as scheduled and completed the study.

### Safety and Reactogenicity

**Figure 2:**
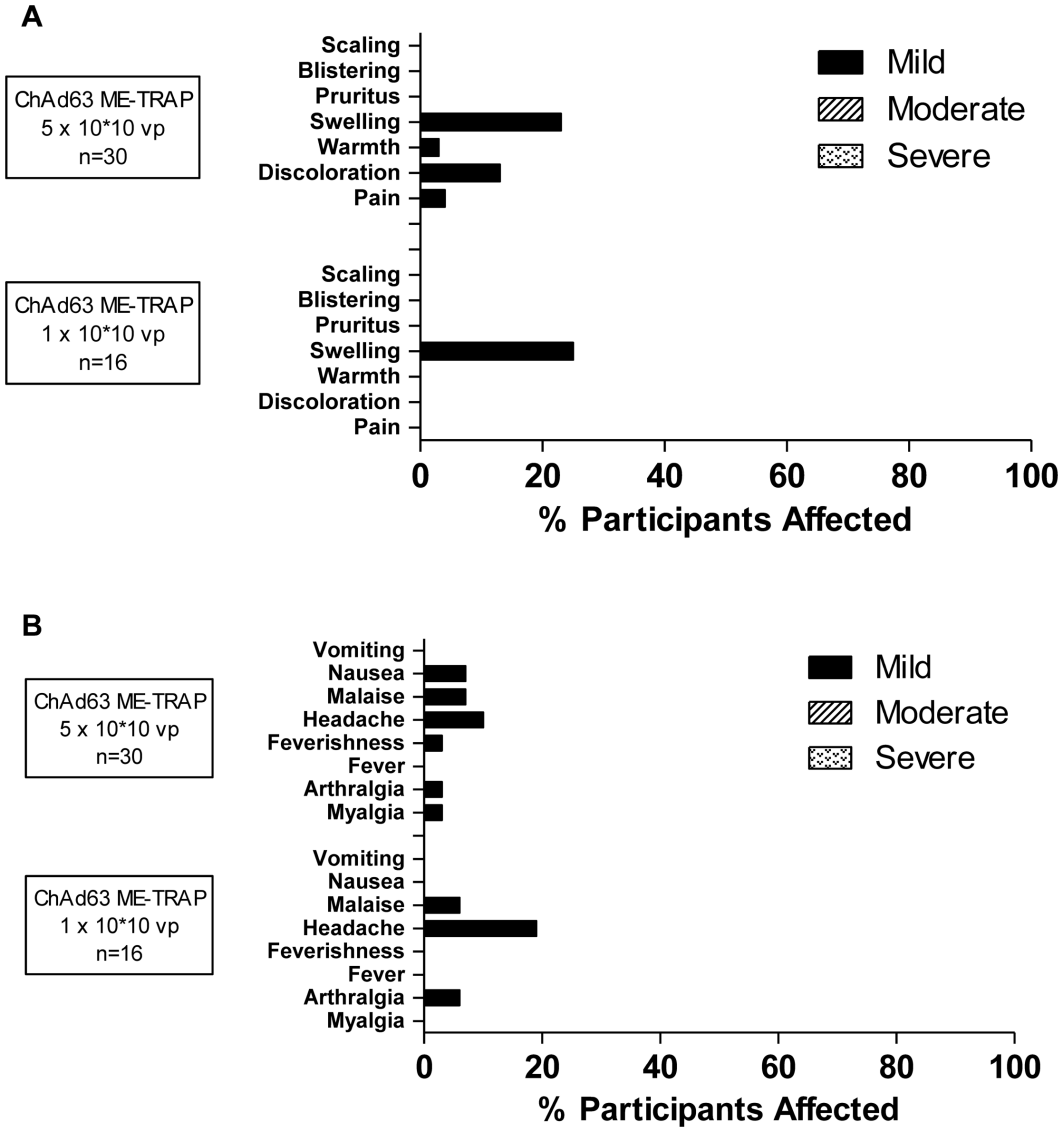
Local and systemic AEs deemed definitely, probably or possibly related to ChAd63 ME-TRAP. Only the highest intensity of each AE per subject is listed. Data are combined for all AEs for all volunteers receiving the same vaccine at the stated dose. There were no immunization related serious AEs. IM = intramuscular. **(A)** Local AEs post ChAd63 ME-TRAP. **(B)** Systemic AEs post ChAd63 ME-TRAP.

**Figure 3:**
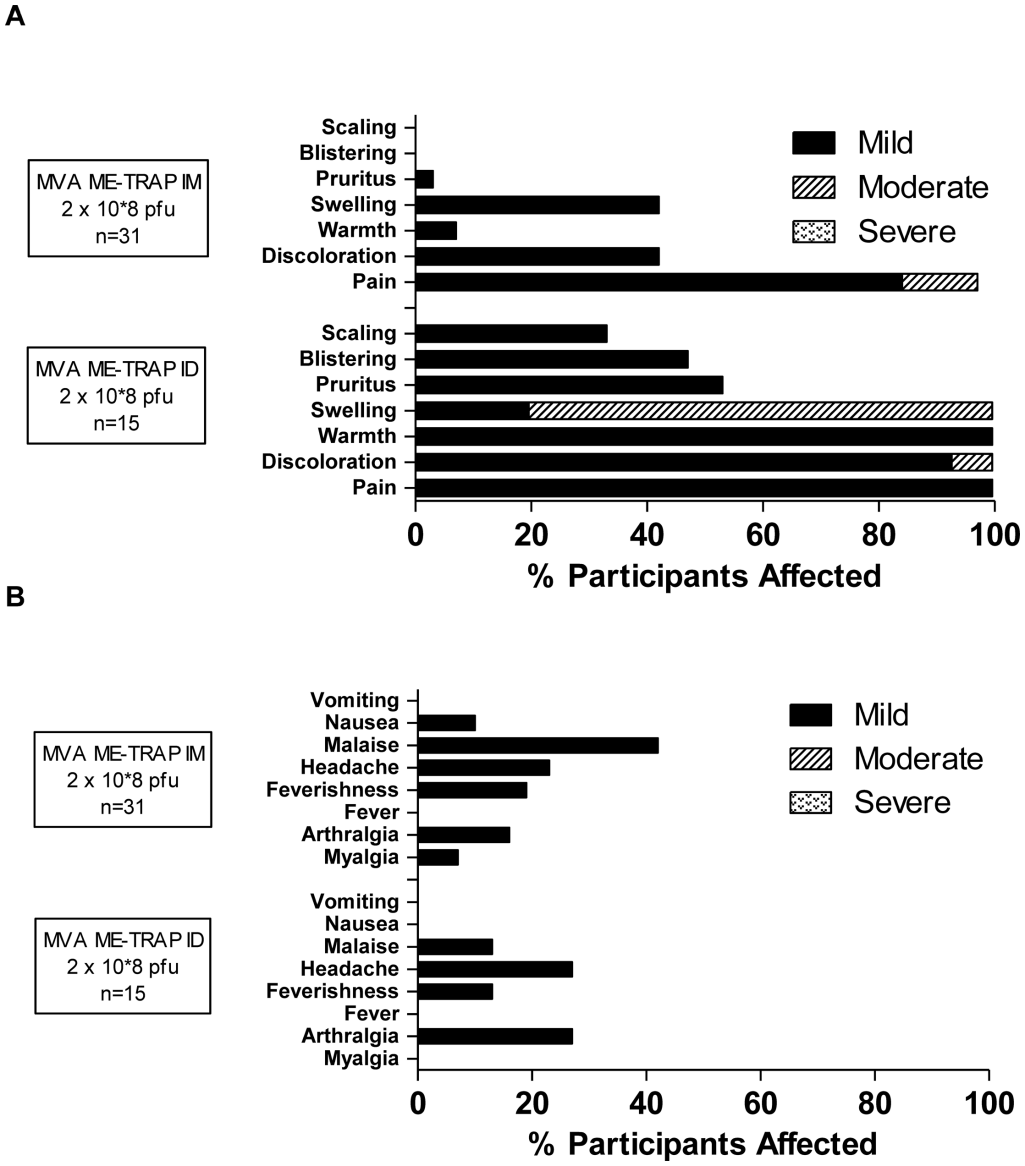
Local and systemic AEs deemed definitely, probably or possibly related to MVA ME-TRAP. Only the highest intensity of each AE per subject is listed. Data are combined for all AEs for all volunteers receiving the same vaccine at the stated dose. There were no immunization related serious AEs. IM = intramuscular. ID = Intradermal. **(A)** Local AEs post MVA ME-TRAP. **(B)** Systemic AEs post MVA ME-TRAP.

No unexpected AEs or SAEs occurred and no volunteers were withdrawn due to AEs. AEs associated with ChAd63 ME-TRAP AEs were all mild in intensity (Figure 2 & Table S2). No clear difference in reactogenicity was noted between participants receiving 1×10^10^ vp and 5×10^10^ vp ChAd63 ME-TRAP. All AEs resolved without sequelae within 72 hours of immunization. MVA ME-TRAP was more reactogenic than ChAd63 ME-TRAP though still well tolerated with the majority of AEs mild in intensity (Figure 3 & Table S2). Whilst systemic reactogenicity of MVA ME-TRAP was unaffected by route of administration, intradermal injection was associated with increased local reactogenicity (namely injection site swelling, warmth, discoloration, blistering and pain) compared to intramuscular administration. All AEs resolved without sequelae. Local AEs post-MVA ME-TRAP resolved between 1 to 15 days post immunization apart from one case of swelling that lasted for 30 days occurring in an individual who received MVA ME-TRAP intradermally (maximum 35 mm of swelling gradually resolving over time, not associated with a sterile abscess). Systemic AEs post-MVA ME-TRAP resolved within 48 hours of immunization. Minor laboratory abnormalities were seen post immunization. However, all were mild, clinically insignificant and resolved fully (Table S3). In Trial A, two volunteers were diagnosed and treated for *P. falciparum* malaria by First response® Rapid Diagnostic kits; one volunteer in group 1, 6 months post MVA ME-TRAP and another in group 2, 34 days post MVA ME-TRAP. These volunteers demonstrated no unusual features of the illness and were included in the final analyses. Similarly, 2 volunteers in Trial B were diagnosed and treated for *P. falciparum* malaria by blood film, one on day 17 and one on day 139 and were included in the final analyses. These volunteers demonstrated no unusual features of the illness.

### Immunogenicity

**Figure 4:**
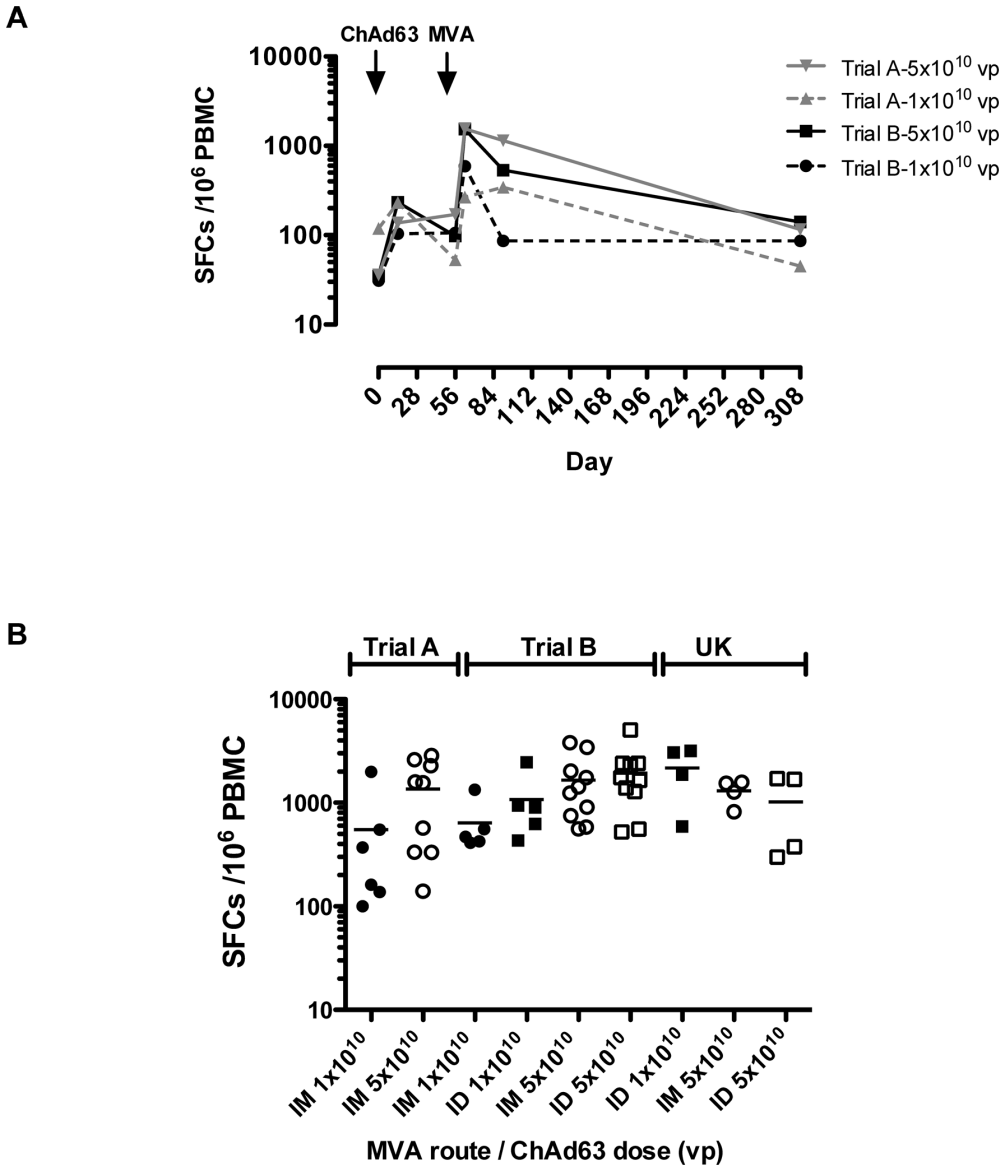
IFN-γ ELISPOT responses to ChAd63-MVA ME-TRAP. (**A**) Time course of IFN-γ ELISPOT responses to ChAd63-MVA ME-TRAP. Lines show median immune response to high dose (solid line) and lower dose (dashed line) of ChAd63 ME-TRAP in Trial A (grey line) and Trial B (black line). For Trial B medians are for groups with MVA given IM and ID combined. (**B**) Peak immune response (day 63–7 days post MVA vaccination) to ChAd63 and MVA ME-TRAP stratified by route of administration of MVA, dose of ChAd63 and trial site. Bar represents geometric mean. Circles represent MVA given IM, squares represent MVA given ID. Closed symbols represent 1×10^10^ vp ChAd63 and open symbols 5×10^10^ vp ChAd63.

Heterologous prime boost with ChAd63-MVA ME-TRAP induced high frequencies of antigen-specific IFNγ-secreting T cells in both trials as measured by ex-vivo IFNγ ELISPOT. Peak IFNγ ELISPOT responses were detected 7 days post MVA ME-TRAP when a positive response (defined as responses above the lower limit of detection and at least double the response measured at Day 0) was detected in 90% and 100% of recipients in Trial A and Trial B respectively (Table 6 & Figure 4A). Responses were well maintained post immunization and detectable in 88% of all vaccinees 9 months after the final immunization (median 116 SFC/10^6^ PBMC, 95% CI 133, 268).

**Table 6:** Comparison of IFN-γ ELISPOT data between African & UK volunteers receiving ChAd63-MVA at peak of vaccine induced immune response (7 days post immunization with MVA ME-TRAP 2×10^8^ pfu).

Trial Site	Kenya	Kenya	Kenya	Kenya	Gambia	Gambia	Oxford	Oxford	Oxford
Route of Admin	IM	ID	IM	ID	IM	IM	ID	IM	ID
**MVA ME-TRAP**									
Dose **ChAd63 ME-TRAP** vp	1×10^10^	1×10^10^	5×10^10^	5×10^10^	1×10^10^	5×10^10^	1×10^10^	5×10^10^	5×10^10^
(All IM)									
Number Participants	5	5	10	10	6	9	4	4	4
Median	426	906	1334	1699	266	1558	2465	1410	1031
IQR	208–945	529–1704	712–2382	1101–2410	129–909	333–2443	910–3138	932–1571	319–1707

Values are SFC per million PBMC for summated peptide pools spanning the length of the ME-TRAP insert tested in duplicate with response to negative (medium) control wells subtracted. IM = intramuscular administration. ID = intradermal administration. Vp = virus particles.

In Trial A there was no statistically significant difference in peak IFNγ ELISPOT response between individuals receiving 1×1010 vp ChAd63 ME-TRAP (median 266 SFC/106 PBMC, 95% CI -208, 1310) and individuals receiving 5×1010 vp ChAd63 ME-TRAP (1558 SFC/106 PBMC, 95% CI 550, 2179) (p = 0.11; Mann Whitney U, 2 tailed test). In contrast in Trial B, the median peak IFNγ ELISPOT response elicited in individuals receiving 5×1010 vp ChAd63 (1536 SFC/106 PBMC (95% CI 1230, 2355) was significantly greater than the peak response in individuals receiving 1×1010 vp ChAd63 ME-TRAP (590 SFC/106 PBMC, 95% CI 399, 1314, p = 0.011 2 tailed Mann Whitney test).

In Trial B there was no significant difference in peak immune response between volunteers receiving MVA administered intramuscularly or intradermally for either dose of ChAd63 ME-TRAP (ChAd63 ME-TRAP 1×1010 vp p = 0.22; ChAd63 ME-TRAP 5×1010 vp p = 0.62, 2-tailed Mann Whitney test) (Figure 4B and Table 6).

ELISPOT data was combined from both trials and analysed using a multivariate linear regression model. Data was stratified by dose of ChAd63 ME-TRAP and trial site. Only dose of ChAd63 ME-TRAP had a significant effect with a 2.3 (95% CI 1.4–3.8) fold increase in mean ELISPOT response in the individuals receiving 5×1010 vp. Of note, route of administration and trial site did not have significant effects on outcome (0.8× fold increase [95% CI 0.4–1.4], p = 0.4 for IM versus ID, 1.6×fold increase [95% CI 0.9–3.0], p = 0.1 for Kenya versus The Gambia).

## Discussion

In these two Phase Ib trials we have shown in healthy, malaria-exposed adult volunteers that a recombinant ChAd63-MVA heterologous prime-boost immunization regimen encoding ME-TRAP is safe as well as very immunogenic for T-cell induction. ChAd63 ME-TRAP demonstrated an excellent safety profile, inducing only a small number of AEs, all of which were mild in intensity. ChAd63 ME-TRAP had a similar reactogenicity profile in our malaria exposed population to that seen in UK volunteers who received comparable doses of ChAd63 ME-TRAP [35]. No clear increase in reactogenicity was noted with the dose escalation of ChAd63, consistent with data from UK volunteers [35]. These findings add to the growing evidence that ChAd63 is a safe vector for clinical use [35], [43], [44].

The safety and immunogenicity of intradermally administered MVA ME-TRAP at doses of up to 1.5×10^8^ pfu MVA ME-TRAP have previously been assessed in malaria exposed adults [28]–[30]. Our data, presented here show that increasing the dose of MVA ME-TRAP administered intradermally to malaria exposed adults to 2×10^8^ pfu is associated with an increase in frequency but not severity of local AEs, causing relatively short-lived injection site pain, discoloration, warmth and swelling (with the exception of one case of moderate injection site swelling lasting 30 days). The increased dose of MVA ME-TRAP did not however translate into an increase in systemic reactogenicity. MVA ME-TRAP administered intramuscularly in our subjects was associated with considerably fewer local AEs than intradermal administration; however, short-lived injection site pain was still reported by 97% of volunteers. This finding was also seen in UK volunteers [35].

In agreement with data from previous clinical studies of ChAd63 vectored vaccines,[35], [43] there was a significant increase in IFNγ responses post MVA boost in groups receiving the higher dose of ChAd63 ME-TRAP (5×10^10^ vp). Of note, there was no statistically significant difference in peak immune responses between individuals in Trial B who received MVA ME-TRAP administered intradermally or intramuscularly. Given this finding and the increased frequency of local AEs associated with intradermal administration, future studies in adults will use 2×10^8^ pfu MVA ME-TRAP administered intramuscularly.

Whilst a previous study of vectored malaria vaccines observed a reduction in T-cell immunogenicity in malaria exposed populations compared to UK volunteers [32], IFNγ responses in our volunteers receiving 5×10^10^ vp ChAd63 ME-TRAP were comparable with Phase Ia data (Table 6) [35]. Further analysis will assess the cellular composition of T-cell responses to assess and quantify mono-functional gamma-interferon-secreting CD8+ T cells as potential markers of vaccine efficacy (*Ewer et al. submitted*). However, importantly the vaccine-induced T cell responses reported here appear to be the most potent reported to date in Africa for any vaccine type. Responses exceeding 1000 SFU /million PBMCs are very difficult to induce with any vaccination strategy and the levels attained here in this first study of ChAd63-MVA in Africa are therefore encouraging for more widespread use of these vaccine vectors.

Concerns have been raised that pre-existing anti-vector immunity could limit the immunogenicity or compromise safety of adenoviral vectored vaccines in exposed populations[45], [46]. Low prevalence of serum neutralising antibodies to ChAd63 in the target population [46] and the proven potency of ChAd63 in pre-clinical and clinical studies [35], [43], [44], [47], including those studied here, make this a promising vector. Further analysis will assess the relationship between baseline anti-ChAd63 antibodies and immunogenicity.

Chimpanzee adenoviruses were first used as a vaccine in humans in 2007 [35] and are now in clinical development for HIV (*Hanke et al unpublished*), Hepatitis C [47], pandemic influenza (*Gilbert et al. unpublished*) as well as for liver-stage and blood-stage malaria [43], [48]. All of these vaccines will have major target populations in Africa and these initial safety and immunogenicity data from African vaccinees are therefore of interest for many disease areas.

Future Phase Ib studies will now assess the safety and immunogenicity of ChAd63-MVA ME-TRAP in children and infants. If these data are favourable, field efficacy studies will be undertaken in infants to assess whether strong cellular immunity against ME-TRAP can translate into significant efficacy against naturally acquired *P. falciparum* infection and disease in the field.

## Supporting Information

Table S1 **TRAP T9/96 and 3D7 peptide sequences and peptide pools.** The sequences in bold represent the 3D7 strain sequences that differ from the T9/96 strain. When no sequence is present for the 3D7 strain, it means that both the 3D7 and T9/96 sequences are identical and the T9/96 peptide has been used in the 3D7 pool.
(PDF)

Table S2 **Local and systemic AEs deemed definitely, probably or possibly related to ChAd63 ME-TRAP or MVA ME-TRAP.** Only the highest intensity of each AE per subject is listed. Data are combined for all AEs for all volunteers receiving the same vaccine at the stated dose. Number  =  number of volunteers experiencing named AE. %  =  percentage of immunised volunteers experiencing named AE. There were no immunization related serious AEs. IM  =  intramuscular administration. ID  =  intradermal administration.
(PDF)

Table S3 **Laboratory abnormalities post immunization deemed definitely, probably or possibly related to ChAd63 ME-TRAP or MVA ME-TRAP.** All were mild, deemed possibly related to vaccination and resolved fully with no long term sequelae. None of the laboratory abnormalities were deemed clinically significant. All laboratory abnormalities resolved by time of next venepuncture* (duration of abnormality is therefore likely to be overestimated, as the abnormality may have resolved prior to retesting). ALT  =  alanine aminotransferase.
(PDF)

Checklist S1 **CONSORT Checklist.**
(DOC)

Protocol S1 **Clinical trial protocol for Trial A.**
(PDF)

Protocol S2 **Clinical trial protocol for Trial B.**
(PDF)
