# Supplementary material for: Safety and Immunogenicity of Heterologous Prime-Boost Immunisation with Plasmodium falciparum Malaria Candidate Vaccines, ChAd63 ME-TRAP and MVA ME-TRAP, in Healthy Gambian and Kenyan Adults
Source: PLoS One. 2013 Mar 19;8(3):e57726. doi: 10.1371/journal.pone.0057726 (PMC3602521; doi:10.1371/journal.pone.0057726)
Supplement: Table S1 — TRAP T9/96 and 3D7 peptide sequences and peptide pools. The sequences in bold represent the 3D7 strain sequences that differ from the T9/96 strain. When no sequence is present for the 3D7 strain, it means that both the 3D7 and T9/96 sequences are identical and the T9/96 peptide has been used in the 3D7 pool. (PDF) [file pone.0057726.s001.pdf]

Table S1: TRAP T9/96 and 3D7 peptide sequences and peptide pools

Peptide Name	Peptide sequence T9/96	Peptide sequence 3D7	T9/96 Peptide Pool	3D7 Peptide Pool
TRAP-1	MNHLGNVKYLVIVFLIFFDL		TT1-10	TD1-10
TRAP-2	VIVFLIFFDLFLVNGRDVQN		TT1-10	TD1-10
TRAP-3	FLVNGRDVQNNIVDEIKYSE	**FLVNGRDVQNNIVDEIKYRE**	TT1-10	TD1-10
TRAP-4	NIVDEIKYSEEVCNDQVDLY	**NIVDEIKYREEVCNDEVDLY**	TT1-10	TD1-10
TRAP-5	EVCNDQVDLYLLMDCSGSIR	**EVCNDEVDLYLLMDCSGSIR**	TT1-10	TD1-10
TRAP-6	LLMDCSGSIRRHNWVNHAVP		TT1-10	TD1-10
TRAP-7	RHNWVNHAVPLAMKLIQQLN		TT1-10	TD1-10
TRAP-8	LAMKLIQQLNLNDNAIHLYV	**LAMKLIQQLNLNDNAIHLYA**	TT1-10	TD1-10
TRAP-9	LNDNAIHLYVNVFSNNAKEI	**LNDNAIHLYASVFSNNAREI**	TT1-10	TD1-10
TRAP-10	LNDNAIHLYVNVFSNNAKEI	**SVFSNNAREIIRLHSDASKN**	TT1-10	TD1-10
TRAP-11	IRLHSDASKNKEKALIIIRS	**IRLHSDASKNKEKALIIIKS**	TT11-20	TD11-20
TRAP-12	KEKALIIIRSLLSTNLPYGR	**KEKALIIIKSLLSTNLPYGK**	TT11-20	TD11-20
TRAP-13	LLSTNLPYGRTNLTDALLQV	**LLSTNLPYGKTNLTDALLQV**	TT11-20	TD11-20
TRAP-14	TNLTDALLQVRKHLNDRINR		TT11-20	TD11-20
TRAP-15	RKHLNDRINRENANQLVVIL		TT11-20	TD11-20
TRAP-16	ENANQLVVILTDGIPDSIQD		TT11-20	TD11-20
TRAP-17	TDGIPDSIQDSLKESRKLSD		TT11-20	TD11-20
TRAP-18	SLKESRKLSDRGVKIAVFGI		TT11-20	TD11-20
TRAP-19	RGVKIAVFGIGQGINVAFNR		TT11-20	TD11-20
TRAP-20	GQGINVAFNRFLVGCHPSDG		TT11-20	TD11-20
TRAP-21	FLVGCHPSDGKCNLYADSAW		TT21-30	TD21-30
TRAP-22	KCNLYADSAWENVKNVIGPF		TT21-30	TD21-30
TRAP-23	ENVKNVIGPFMKAVCVEVEK		TT21-30	TD21-30
TRAP-24	MKAVCVEVEKTASCGVWDEW		TT21-30	TD21-30
TRAP-25	TASCGVWDEWSPCSVTCGKG		TT21-30	TD21-30
TRAP-26	SPCSVTCGKGTRSRKREILH		TT21-30	TD21-30
TRAP-27	TRSRKREILHEGCTSEIQEQ	**TRSRKREILHEGCTSELQEQ**	TT21-30	TD21-30
TRAP-28	EGCTSEIQEQCEEERCPPKW	**EGCTSELQEQCEEERCLPKR**	TT21-30	TD21-30
TRAP-29	CEEERCPPKWEPLDVPDEPE	**CEEERCLPKREPLDVPDEPE**	TT21-30	TD21-30
TRAP-30	EPLDVPDEPEDDQPRPRGDN		TT21-30	TD21-30
TRAP-31	DDQPRPRGDNSSVQKPEENI	**DDQPRPRGDNFAVEKPNENI**	TT31-40	TD31-40
TRAP-32	SSVQKPEENIIDNNPQEPSP	**FAVEKPNENIIDNNPQEPSP**	TT31-40	TD31-40
TRAP-33	IDNNPQEPSPNPEEGKDENP	**IDNNPQEPSPNPEEGKGENP**	TT31-40	TD31-40
TRAP-34	NPEEGKDENPNGFDLDENPE	**NPEEGKGENPNGFDLDENPE**	TT31-40	TD31-40
TRAP-35	NGFDLDENPENPPNPDIPEQ	**NGFDLDENPENPPNPPNPPN**	TT31-40	TD31-40
TRAP-36	NPPNPDIPEQKPNIPEDSEK	**NPPNPPNPPNPPNPPNPPNP**	TT31-40	TD31-40
TRAP-37	*NONE*	**PPNPPNPPNPDIPEQKPNIP**	TT31-40	TD31-40
TRAP-38	DIPEQKPNIPEDSEKEVPSD	**DIPEQKPNIPEDSEKEVPSD**	TT31-40	TD31-40
TRAP-39	EDSEKEVPSDVPKNPEDDRE		TT31-40	TD31-40
TRAP-40	VPKNPEDDREENFDIPKKPE		TT31-40	TD31-40
TRAP-41	ENFDIPKKPENKHDNQNNLP		TT41-50	TD41-50
TRAP-42	NKHDNQNNLPNDKSDRNIPY	**NKHDNQNNLPNDKSDRYIPY**	TT41-50	TD41-50
TRAP-43	NDKSDRNIPYSPLPPKVLDN	**NDKSDRYIPYSPLAPKVLDN**	TT41-50	TD41-50
TRAP-44	SPLPPKVLDNERKQSDPQSQ	**SPLAPKVLDNERKQSDPQSQ**	TT41-50	TD41-50
TRAP-45	ERKQSDPQSQDNNGNRHVPN		TT41-50	TD41-50
TRAP-46	DNNGNRHVPNSEDRETRPHG		TT41-50	TD41-50
TRAP-47	SEDRETRPHGRNNENRSYNR		TT41-50	TD41-50
TRAP-48	RNNENRSYNRKYNDTPKHPE		TT41-50	TD41-50
TRAP-49	KYNDTPKHPEREEHEKPDNN		TT41-50	TD41-50

TRAP-50	REEHEKPDNNKKKGESDNKY		TT41-50	TD41-50
TRAP-51	KKKGESDNKYKIAGGIAGGL		TT51-57	TT51-57
TRAP-52	KIAGGIAGGLALLACAGLAY		TT51-57	TT51-57
TRAP-53	ALLACAGLAYKFVVPGAATP		TT51-57	TT51-57
TRAP-54	KFVVPGAATPYAGEPAPFDE		TT51-57	TT51-57
TRAP-55	YAGEPAPFDETLGEEDKDLD		TT51-57	TT51-57
TRAP-56	TLGEEDKDLDEPEQFRLPEE		TT51-57	TT51-57
TRAP-57	EPEQFRLPEENEWN		TT51-57	TT51-57
