# Supplementary material for: Safety and Immunogenicity of Heterologous Prime-Boost Immunisation with Plasmodium falciparum Malaria Candidate Vaccines, ChAd63 ME-TRAP and MVA ME-TRAP, in Healthy Gambian and Kenyan Adults
Source: PLoS One. 2013 Mar 19;8(3):e57726. doi: 10.1371/journal.pone.0057726 (PMC3602521; doi:10.1371/journal.pone.0057726)
Supplement: Table S2 — Local and systemic AEs deemed definitely, probably or possibly related to ChAd63 ME-TRAP or MVA ME-TRAP. Only the highest intensity of each AE per subject is listed. Data are combined for all AEs for all volunteers receiving the same vaccine at the stated dose. Number = number of volunteers experiencing named AE. % = percentage of immunised volunteers experiencing named AE. There were no immunization related serious AEs. IM = intramuscular administration. ID = intradermal administration. (PDF) [file pone.0057726.s002.pdf]

**Table S2: AE Data**

| | ChAd63 ME-TRAP | | MVA ME-TRAP 2 x $10^8$ pfu | |
|---|---|---|---|---|
| | 5 x $10^9$ vp | 5 x $10^{10}$ vp | IM | ID |
| | n=16 | n=30 | n=31 | n=15 |
| **LOCAL AEs** | | | | |
| Pain | 0 | 12 (4%) | 30 (96.8%) | 15 (100%) |
| Swelling | 4 (25%) | 7 (23.3%) | 13 (41.9%) | 15 (100%) |
| Discoloration | 0 | 4 (13.3%) | 13 (41.9%) | 14 (93.3%) |
| Warmth | 0 | 1 (3.3%) | 2 (6.5%) | 15 (100%) |
| Itch | 0 | 0 | 1 (3.2%) | 8 (53.3%) |
| Blistering | 0 | 0 | 0 | 7 (46.7%) |
| Scaling | 0 | 0 | 0 | 5 (33.3%) |
| | | | | |
| **SYSTEMIC AEs** | | | | |
| Headache | 3 (18.8%) | 3 (10%) | 7 (22.6%) | 4 (26.7%) |
| Malaise | 1 (6%) | 2 (6.7%) | 13 (41.9%) | 2 (13.3%) |
| Arthralgia | 1 (6%) | 1 (3.3%) | 5 (16.1%) | 4 (26.7%) |
| Feverish | 0 | 1 (3.3%) | 6 (19.4%) | 2 (13.3%) |
| Nausea | 0 | 2 (6.7%) | 3 (9.7%) | 0 |
| Myalgia | 0 | 1 (3.3%) | 2 (6.5%) | 0 |
| Fever | 0 | 0 | 0 | 0 |
