# Supplementary material for: Safety and Immunogenicity of Heterologous Prime-Boost Immunisation with Plasmodium falciparum Malaria Candidate Vaccines, ChAd63 ME-TRAP and MVA ME-TRAP, in Healthy Gambian and Kenyan Adults
Source: PLoS One. 2013 Mar 19;8(3):e57726. doi: 10.1371/journal.pone.0057726 (PMC3602521; doi:10.1371/journal.pone.0057726)
Supplement: Table S3 — Laboratory abnormalities post immunization deemed definitely, probably or possibly related to ChAd63 ME-TRAP or MVA ME-TRAP. All were mild, deemed possibly related to vaccination and resolved fully with no long term sequelae. None of the laboratory abnormalities were deemed clinically significant. All laboratory abnormalities resolved by time of next venepuncture* (duration of abnormality is therefore likely to be overestimated, as the abnormality may have resolved prior to retesting). ALT = alanine aminotransferase. (PDF) [file pone.0057726.s003.pdf]

Table S3: Laboratory abnormalities post immunization

Laboratory Abnormality	Vaccine	Dose	Onset post immunization (days)	Duration (days)*
Increased Haematocrit	ChAd63 ME-TRAP	$1x10^{10}$ vp	14	42
Increased Haematocrit	ChAd63 ME-TRAP	$5x10^{10}$ vp	14	42
Thrombocytosis	ChAd63 ME-TRAP	$5x10^{10}$ vp	14	42
Elevated ALT	ChAd63 ME-TRAP	$5x10^{10}$ vp	14	42
Elevated ALT	MVA ME-TRAP(IM)	2×10^{8} pfu	35	217
