# Supplementary material for: Safety and Immunogenicity of Heterologous Prime-Boost Immunisation with Plasmodium falciparum Malaria Candidate Vaccines, ChAd63 ME-TRAP and MVA ME-TRAP, in Healthy Gambian and Kenyan Adults
Source: PLoS One. 2013 Mar 19;8(3):e57726. doi: 10.1371/journal.pone.0057726 (PMC3602521; doi:10.1371/journal.pone.0057726)
Supplement: Protocol S1 — Clinical trial protocol for Trial A. (PDF) [file pone.0057726.s005.pdf]

© University of Oxford 2009

Safety and immunogenicity of heterologous prime-boost with the candidate malaria vaccines AdCh63 ME-TRAP and MVA ME-TRAP in healthy adults and children in a malaria endemic area

A Phase I vaccine trial

Trial Number:	VAC 041
Protocol Version:	6.0
Date:	26th October , 2010
OXTREC Number:	64-09
SCC Number:	SCC 1186
Sponsor:	University of Oxford
Funding body:	EDCTP
Author(s):	Christopher JA Duncan, Susanne HC Sheehy, Nicholas Anagnostou, Alison Lawrie, Kalifa Bojang, Katie Flanagan, Jenny Mueller, Adrian VS Hill

© University of Oxford 2009

STATEMENT OF COMPLIANCE

The trial will be conducted in compliance with the protocol, International Conference on Harmonisation Good Clinical Practice Guideline E6 (R1) (ICH-GCP) and the applicable regulatory requirements.

SIGNATURE PAGE

PROTOCOL SIGNATURE SHEET

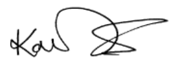

Principal Investigator: ________________________________ Date: 26th October 2010

Confidentiality Statement
This document contains confidential information that must not be disclosed to anyone other than the Sponsor, the Investigator Team, members of the Independent Ethics Committee and the Gambia Medicines Board. This information cannot be used for any purpose other than the evaluation or conduct of the clinical investigation without the prior written consent of Professor Adrian Hill.

Contents

Statement of Compliance 2
Signature Page 2
Contents 3
Key Roles and General Information 4
List of abbreviations 6
List of protocol amendments 7
1. Study Summary 9
2. Background Information 12
3. Objectives 20
4. Study Design 20
5. Selection and Withdrawal of Subjects 24
6. Randomisation 25
7. Blinding 25
8. Investigational Medicinal Product 25
9. Study Schedule 30
10. Study Procedures 32
11. Assessments and Evaluations 35
12. Statistics 40
13. Data Handling and Record Keeping 40
14. Data Access and Quality Assurance 41
15. Finance 42
16. Ethical Considerations 42
17. Indemnity/Compensation/Insurance 44
18. References 44

© University of Oxford 2009

KEY ROLES AND GENERAL INFORMATION

**Trial Centres:**	Medical Research Council Laboratories<br>PO Box 273, Banjul<br>The Gambia<br><br>Sukuta Health Centre<br>The Gambia
**Principal Investigator:**	Dr Kalifa Bojang<br>Medical Research Council<br>PO Box 273, Banjul<br>The Gambia
**Study Physician:**	**Dr** Muhammed Afolabi
**Co-Investigator(s):**	Dr Katie Flanagan<br>PO Box 273, Medical Research Council<br>Banjul<br>The Gambia<br><br>Professor Adrian V.S. Hill<br>Dr Christopher J.A. Duncan<br>Dr Susanne H.C. Sheehy<br>Dr Nicholas A Anagnostou<br>Centre for Clinical Vaccinology and Tropical Medicine<br>University of Oxford<br>Churchill Hospital, Oxford OX3 7LJ
L**ocal Safety Monitor:**	Professor Ousman Nyan<br>University of The Gambia<br>P.O. Box 3530<br>Serrekunda, The Gambia<br>West Africa
**Data Safety and Monitoring Board:**	Chair:<br>Professor Geoffrey Targett<br>Emeritus Professor of Immunology of Parasitic Diseases<br>London School of Hygiene & Tropical Medicine<br>Room 410, Keppel Street, London WC1E 7HT, UK<br><br>DSMB members:<br><br>Dr. Paul Milligan<br>London School of Hygiene & Tropical Medicine<br>Reader in Epidemiology and Medical Statistics<br>Room 411a, Keppel Street, London WC1E 7HT, UK

Professor Mahamadou Thera
MRTC, Bamako, University of Mali
Colline de Badalabougou
BP E2528, Mali

Dr. Bernhards Ogutu
KEMRI
P.O. Box 2254 - 00202
Nairobi
Kenya

Project Manager (Gambia): Abdoulie Jadama
PO Box 273, Medical Research Council
Banjul
The Gambia

Project Manager (Oxford): Dr Katherine Gantlett
Vaccine Program Coordinator
Centre for Clinical Vaccinology and Tropical Medicine
University of Oxford
Churchill Hospital, Oxford OX3 7LJ

Senior Immunologist: Dr Katie Flanagan
PO Box 273, Medical Research Council
Banjul
The Gambia

Sponsor: University of Oxford
Named contact: Heather House
Clinical Trials and Research Governance
Manor House
John Radcliffe Hospital
Oxford, OX3 9DZ

Monitor: Ceri McKenna
Appledown Clinical Research Limited
Orchard's End,
Greenland's Lane,
Prestwood,
Gt. Missenden,
Bucks. HP16 9QX

LIST OF ABBREVIATIONS

**AdCh63**	Chimpanzee Adenovirus 63
**AE**	Adverse event
**ALT**	Alanine aminotransferase
**CCVTM**	Clinical Centre for Vaccinology and Tropical Medicine
**CBF**	Clinical Biomanufacturing Facility
**CMI**	Cell-mediated immunity
**CRF**	Case report form
**CTL**	Cytotoxic T lymphocytes
**DSMB**	Data Safety and Monitoring Board
**ELISA**	Enzyme-linked immunosorbant assay
**ELISPOT**	Enzyme-linked immunospot
**FBC**	Full blood count
**FP9**	Fowlpox 9
**GCP**	Good Clinical Practice
**GMP**	Good Manufacturing Practice
**IB**	Investigator's Brochure
**ICS**	Intracellular Cytokine Staining
**IDT**	Impfstoffwerk Dessau-Tornau GmbH
**IEC**	Independent Ethics Committee
**IFN-γ**	Gamma interferon
**IMP**	Investigational Medicinal Product
**IRB**	Independent Review Board
**HBV**	Hepatitis B virus
HDCRV	Human Diploid Cell Rabies Vaccine
**HIV**	Human immunodeficiency virus
**HLA**	Human leukocyte antigen
**LFT**	Liver function test
**LSM**	Local safety monitor
**ME-TRAP**	Multiple epitope string with thrombospondin-related adhesion protein
**MHRA**	Medicines and Healthcare products Regulatory Agency
**MVA**	Modified vaccinia Virus Ankara
**NHS**	National Health Service
**OXTREC**	Oxford Tropical Research Ethics Committee
**PBMC**	Peripheral blood mononuclear cells
**PCR**	Polymerase chain reaction
**pfu**	Plaque forming units
**SAE**	Serious adverse event
**SOP**	Standard Operating Procedures
**SUSAR**	Suspected unexpected serious adverse reaction
**vp**	Virus particles

© University of Oxford 2009

LIST OF PROTOCOL AMENDMENTS

Version 2.0 9th December 2009 Dr S Sheehy, Dr C Duncan

Section	Section Title	Modification
2.1	Background Information	Updated
2.2	Rationale	Addition of sero-survey to assess local seroprevalence of neutralising antibodies to AdCh63.<br>Addition of immunogenicity criterion for enrolment of group 2.
7.4	Dispensing & Administration	Addition of precautions for the use of GMO products
7.5	Trial Regime	Addition of immunogenicity criterion for enrolment of group 2

Version 3.0 3rd March 2010 Dr Kalifa Bojang

Section	Section Title	Modification
Title page and sections 1, 2.2, 2.3, 3, 4.1, 4.2, 4.4, 5.1, 7.5, 8.1, 10.3.6, 11, 15.3 and 16.2	Protocol Title and Study Summary, Rationale, Risks and Benefits, Objectives, Description and Justification of Study Design, Study groups, Safety, Inclusion Criteria, Trial regimen, Screening, Collection of Adverse Events, Statistics, Informed Consent and Compensation	Updated to reflect the fact that the protocol is only for the adult part of the study
Title page	Authors	Kalifa Bojang and Katie Flanagan included
Key Roles and General Information	Trial Physician<br>Project manager	Muhammed Afolabi appointed<br>Abdoulie Jadama
List of Abbreviations	List of Abbreviations	List updated
Appendix A	Tables' numbering	Table numbering adjusted

Version 4.0 23rd August 2010 Dr S. Sheehy, Dr C.Duncan, Dr K. Bojang, Prof A.V.S Hill

Section	Section Title	Modification
Title page and sections 1, 2.2, 2.3, 3, 4.1, 4.2, 4.4, 5.1, 7.5, 8.1, 10.3.6, 11, 15.3 and 16.2	Protocol Title and Study Summary, Rationale, Risks and Benefits, Objectives, Description	Updated to add paediatric part of the study

	and Justification of Study Design, Study groups, Safety, Inclusion Criteria, Trial regimen, Screening, Collection of Adverse Events, Statistics, Informed Consent and Compensation	
Appendix C	-	Removed and IB updated

Version 5.0 14th September 2010 Dr S. Sheehy, Dr C.Duncan
Dr K. Bojang, Prof A.V.S Hill

Section	Section Title	Modification
5.2	Exclusion Criteria	Positive malaria rapid antigen test
9.1	Screening	Inclusion of malaria rapid antigen test negative and exclusion of antigen test positive children
17.2	Compensation	Carers of children will be offered compensation for transport expenses

Version 6.0 25th October 2010 Dr N Anagnostou, Dr S Sheehy, Prof AVS Hill

Section	Section Title	Modification
Section 1; Section 10.1	Study Summary; Clinical Evaluations	Modification of time windows for Clinic Attendance numbers 4 (D56) and 5 (D63)
Section 4.4.1; Section 8.5; Section 12.4.1	Safety Monitoring; Trial Regimen; Safety Review	Description of the new, staged, schedule of vaccination and safety monitoring for groups 2 and 3
Section 5.2	Exclusion criteria	Modification of criteria for exclusion of volunteers on the basis of Haemoglobin, Creatinine and ALT levels
Section 8.2	Formulation	MVA ME-TRAP vials used can also be of a volume of 450 µL
Section 1; Section 10.1	Study Summary; Clinical Evaluations	Blood sample used for HLA typing changed to that from the day of screening. *P. Falciparum* PCR added to tests done on blood taken at D0.
Section 4.3.2; Section 10.1	Secondary and Tertiary Endpoints; Clinical Evaluations	Purpose of polymerase chain reaction (PCR) testing for *P. falciparum*.
Section 10.3.2	Special Laboratory Evaluations	*P. Falciparum* PCR done on blood taken at D0.

1. Study Summary

**Trial Title**	Safety and immunogenicity of a heterologous prime-boost vaccine strategy with AdCh63 ME-TRAP and MVA ME-TRAP in healthy adults and children in a malaria endemic area
**Trial Identifier**<br>**Clinical phase**	VAC 041<br>I
**Active ingredients of vaccines/products**	Chimpanzee Adenovirus 63 expressing multiple epitopes with thrombospondin-related adhesion protein (AdCh63 ME-TRAP)<br><br>Modified vaccinia Virus Ankara expressing multiple epitopes with thrombospondin-related adhesion protein (MVA ME-TRAP)<br><br>Human diploid cell rabies vaccine (HDCRV)
**Finished products**	*AdCh63 ME-TRAP*<br>*MVA ME-TRAP*<br>*HDCRV*
**Dose(s)**	AdCh63 ME-TRAP: 1 x 10^{10}vp and 5 x 10^{10}vp<br>MVA ME-TRAP: 1 x 10^{8} pfu and 2 x 10^{8} pfu<br>HDCRV: 1ml
**Route(s)**	Intramuscular needle injection into deltoid region of arm
**Principal Investigator**<br><br>**Trial Centre**	Dr Kalifa Bojang<br><br>MRC<br>PO Box 273, Banjul<br>The Gambia<br>Sukuta Health Centre<br>The Gambia
**Planned Trial Period**	January 2010 until June 2011
**Study Duration**	12-18 months
**Primary Objective**<br><br>**Secondary Objective**<br><br>**Tertiary Objective**	Safety of a heterologous prime-boost vaccine strategy with AdCh63 ME-TRAP and MVA ME-TRAP in healthy adults and children in a malaria endemic area.<br>Immunogenicity of a heterologous prime-boost vaccine strategy with AdCh63 ME-TRAP and MVA ME-TRAP in healthy adults and children in a malaria endemic area.<br>Descriptive comparison of immunogenicity of two doses of MVA ME-TRAP in children
**Planned Sample Size**	16 adults<br>36 children (aged 2-6 years)
**Vaccination Schedule**<br>**Follow-up duration**	Days 0 and 56<br>10 months from enrolment
**Blood Sampling Schedule**<br>**Primary Evaluation Criteria**	Screening, D0, D14, D56, D63, D90, D300<br>Local and systemic solicited and unsolicited adverse events
**Secondary Evaluation Criteria**	Ex vivo and Cultured ELISPOT,<br>ICS for CD4+ and CD8+ T lymphocytes,<br>ELISA for TRAP antibody,<br>AdCh63 neutralising antibody
**Study Design**	Single blinded randomised controlled dose escalation phase I study

Schematic of Study Design:

Group 1 (Adult males)	A	6 volunteers	AdCh63 ME-TRAP 1×10^{10}vp	MVA ME-TRAP 2×10^{8} pfu
	B	10 volunteers	AdCh63 ME-TRAP 5×10^{10}vp	MVA ME-TRAP 2×10^{8} pfu

Group 2 (Aged 2-6)	A	6 volunteers	AdCh63 ME-TRAP 1×10^{10}vp	MVA ME-TRAP 1×10^{8} pfu
	B	6 volunteers	AdCh63 ME-TRAP 1×10^{10}vp	MVA ME-TRAP 2×10^{8} pfu
	C	6 volunteers	HDCRV 1ml	HDCRV 1ml

Group 3 (Aged 2-6)	A	6 volunteers	AdCh63 ME-TRAP 5×10^{10}vp	MVA ME-TRAP 1×10^{8} pfu
	B	6 volunteers	AdCh63 ME-TRAP 5×10^{10}vp	MVA ME-TRAP 2×10^{8} pfu
	C	6 volunteers	HDCRV 1ml	HDCRV 1ml

	**Clinic Attendance number**	**1**	**2**	**3**	**4**	**5**	**6**	**7**
	**Timeline (days)***	S	D0	D14	D56	D63	D90	D300
	**Time windows (days)**			±7	-7/+30	-1/+7	±14	±28
	**Vaccination**		1		2			
	**Inclusion/Exclusion criteria**	X						
	**Informed consent**	X						
	**Medical history**	X						
	**Phys. exam.**	X						
	**Urinalysis**	X						
	**Review contra-indications**	X	X		X			
	**Post-dose follow-up (days)****			14	56	7	34	244
	**Local & systemic events/reactions**		X	X	X	X	X	X
	**HLA typing (mL)**	3						
	P. falciparum PCR		2					
	**HIV (mL)**	3						
**GROUP 1**	**GROUP 1: Exploratory immunology (incl ELISPOT) and safety bloods (mL)**	7	27	30	30	30	30	30
	**GROUP 1: Blood volume (mL)**	10	30	30	30	30	30	30
	**GROUP 1: Cumulative**	**10**	**40**	**70**	**100**	**130**	**160**	**190**

	**blood volume (mL)**							
**GROUPS 2 & 3**	**GROUPS 2 & 3: Blood volume (mL)**	**5*****	**5******	**5**	**5**	**5**	**5**	**5**
	**GROUPS 2 & 3: Cumulative blood volume (mL)**	**5**	**10**	**15**	**20**	**25**	**30**	**35**

*****Timeline is approximate only. Exact timings of visits relate to the previous visit – *i.e.* each visit must occur the specified number of days after the last visit ± time window
** Volunteers will be followed up daily at home by a field worker for a minimum of three days following each vaccination
*** Includes HLA
**** Includes *P. Falciparum* PCR

2. Background Information

2.1. Background Information

Introduction

Malaria transmission is now falling in some parts of Africa [1-5], leading some to suggest elimination is possible [6]. This is desirable but ambitious: there were 500 million malaria episodes worldwide in 2002, over a million deaths in Africa [7, 8], and previous attempts at elimination have had mixed success [9-11]. It is currently thought that additional control measures will be required [12], including vaccination [13].

Progress towards a malaria vaccine

The candidate pre-erythrocytic malaria vaccine RTS,S, targeting the circumsporozoite protein, is the most advanced malaria vaccine in development. Preliminary estimates of efficacy against infection rates after curative anti-malarial treatment were 34.0% (95%CI 8-53%) in adults [14] and 65.9% (95%CI 43-80) in infants [15]. Efficacy against the more clinically relevant endpoint of clinical malaria in 1-4 year old children was 29.9% (95%CI 11-45%) [16]. Efficacy with the more immunogenic AS01 adjuvant was 52.9% (95%CI 28-69%) in Kenya and Tanzania [17]. A Phase III trial is now in progress. The observed efficacy of RTS,S is partial, indicating that we should continue to develop other candidate vaccines, which could be used in combination with RTS,S if efficacy is established, or give rise to more efficacious approaches. The only other vaccination approach that has demonstrated partial efficacy in humans is using virally vectored vaccines to induce T cells.

T cell immunity in malaria

Malaria immunity is complex, however T cell responses provide protection against malaria in animal models [18], in the field [19-21], following irradiated sporozoite inoculation [22, 23] [24] and following vaccination.
Immunisation of mice with irradiated sporozoites of murine *Plasmodium* provides protection against later challenge with murine malaria sporozoites [18]. This protective immunity can be transferred to non immune mice by transferring the CD8+ T lymphocyte clones specific to pre-erythrocytic malaria surface antigens, the circumsporozoite protein (CS), or thrombospondin related adhesion protein (TRAP) that were induced by irradiated sporozoites [22, 23]. In immune mice, depletion of CD8+ cells renders them susceptible to further infection with *P. berghei* [25]. Incubation of infected hepatocytes with unfractionated spleen cells from immune animals resulted in elimination of infected hepatocytes [26]. This has been shown to be mediated by T lymphocyte recognition of a circumsporozoite protein derived peptide on infected mouse hepatocytes that provokes lysis of the infected cell and parasite death [27]. A recent study has confirmed the protective effect of repeated human inoculation with irradiated sporozoites against experimental *P. falciparum* challenge [24].

The class 1 Human Leucocyte Antigen (HLA) HLA B53, present in West African populations, confers protection against severe forms of malaria. Amongst this group of individuals, HLA B53 restricted cytotoxic T lymphocytes (CD8+) recognise a conserved peptide from liver-stage-specific antigen 1 (LSA-1) [19, 20].
T cell memory responses, quantified by cultured ELISPOT to TRAP antigens, are protective against clinical malaria infection in Kenya [21]. Conversely, *P. falciparum* infection has a deleterious effect on T cell responses, increasing the risk of further malaria episodes [28].

This evidence has prompted the development of a heterologous prime-boost immunisation strategy to induce T cell responses against pre-erythrocytic stages of parasite development. The prime-boost strategy

uses two different vectors to deliver a common antigen construct, which achieves an expansion of T cells reactive to the common antigen, rather than to the vectors used. Numerous vectors have been investigated.

Previous studies with T cell inducing vaccinations

Following challenge studies in Oxford protecting 2 out of 16 volunteers using FP9 ME-TRAP followed by MVA ME-TRAP [29], studies were undertaken in adults and then children in Kilifi [30, 31]. Immunogenicity was lower than expected [28], and efficacy was not seen in a study of 400 children in Kilifi district [32]. Furthermore, FP9 ME-TRAP shows variability in potency by batch [33]. Further development of T cell inducing vaccination in Oxford in pre-clinical studies has therefore examined more immunogenic vectors such as adenovirus vectors, in order to attain greater efficacy.

Pre-clinical studies

Using the chimpanzee adenovirus vectors AdCh6, AdCh7 and AdCh9 encoding ME-TRAP (rendered non-replication competent by deletion of the virus E1 and E3 genes), in a *P. berghei* challenge model it was shown that single immunisation resulted in sterile protection at sporozoite challenge in 67%, 83% and 92% of mice respectively [34]. In contrast, single dose MVA ME-TRAP and FP9 ME-TRAP alone offered no protection [35].

AdCh68 and AdCh63 expressing ME-TRAP generated similar construct specific T cell immunogenicity (measured by splenic peptide-specific interferon-γ (IFN-γ) secreting CD8+ T cells). However at challenge with *P.berghei* AdCh63 generated 83% protection at 20 days compared to 30% protection in the AdCh68 group (A. Reyes-Sandoval, unpublished). Protection was correlated with CD8+ IFN-γ T cells by ICS. Similar studies have compared the potency of the simian adenovirus vectors encoding ME-TRAP. AdCh9 produced the highest levels $CD8^+$ cells, and provided 92% protection to a *P. berghei* sporozoite challenge 14 days later compared to 83% protection with AdHu5 and AdCh7. At 60 days however protection to challenge was reduced to 17% in the AdCh9 group, 50% in the AdCh7 group and was absent in all other groups (A. Reyes Sandoval, submitted). A comparison of AdCh63 and AdCh9 expressing ME-TRAP showed a similar level of splenic IFN-γ secreting cells in mice, but AdCh63 produced high responses when measured in the blood (>20,000 SFC/million PBMCs compared to 12.5 million for AdCh9). After a challenge with *P. berghei* sporozoites, AdCh63 showed 83% protection at 20 days compared to 33% in the AdCh9 group (A. Reyes Sandoval, submitted).

Extensive preclinical toxicology testing in mice using AdCh63 ME-TRAP and MVA ME-TRAP has demonstrated no treatment-related toxicity. Biodistribution studies have shown no evidence of replication within the host. Full details of these studies are contained in the Investigator Brochure for the relevant IMP. AdCh63 ME-TRAP has been safely administered to rhesus macaques and MVA ME-TRAP to Chimpanzees by the intramuscular route [36].

Clinical studies of AdCh63 ME-TRAP & MVA ME-TRAP in Oxford

85 healthy volunteers have received AdCh63 ME-TRAP in Oxford to date. 46 healthy males in The Gambia and Kenya have received AdCh63 ME-TRAP. The safety profile of AdCh63 ME-TRAP is excellent, with tolerable reactogenicity up to 2×10^{11} vp. This compares favourably to the studies of AdHu5 vector (see Investigator Brochure for AdCh63 ME-TRAP). Over 700 healthy volunteers and 5 HIV positive volunteers have received MVA ME-TRAP in Oxford, The Gambia and Kenya [29, 30, 32, 37]. No linked serious adverse events have been reported for either vaccine. MVA ME-TRAP has been administered by the intramuscular route with improved local reactogenicity and similar systemic reactogenicity. Full safety and immunogenicity data is contained in the Investigator Brochures for these IMPs. A further 70 volunteers have received the AdCh63 vector with different malaria inserts (AMA1 and MSP1) with a remarkably similar tolerability profile to AdCh63 ME-TRAP.

Experimentally induced malaria sporozoite infections (termed “sporozoite challenge”) allow direct evaluation of a vaccine’s efficacy prior to field studies. A phase IIa study began in February 2009 to determine the efficacy of AdCh63 ME-TRAP when used alone, and in combination with MVA ME-TRAP.

AdCh63 was unprecedentedly immunogenic, but did not protect any of 10 vaccinated volunteers. The combination of AdCh63 ME-TRAP and MVA ME-TRAP was more immunogenic and protected 3 out of 14 volunteers over two challenges. In addition, 5/14 volunteers had a significant delay to parasitaemia measured by blood film microscopy and quantitative real-time polymerase chain reaction. Protection in these volunteers was correlated with levels of CD8+ IFN-γ T cells by ICS. All of 6 control unvaccinated volunteers developed malaria, as have all the 68 unvaccinated volunteers challenged to date in Oxford [38].

Investigational products relevant to this application

The Modified vaccinia Ankara (MVA) vector
The MVA vector was selected for its safety and immunogenicity profile. Vaccinia was successfully used to vaccinate against and eliminate smallpox. MVA was originally derived from the vaccinia strain Ankara by over 500 serial passages in primary chicken embryo fibroblasts (CEF cells). MVA has six major genomic deletions compared to the parental Ankara genome and cannot replicate in non-transformed mammalian cells. The viral genome has been proven to be stable through a large series of passages in chicken embryo fibroblasts [39]. MVA shows no cytopathic effect or plaque formation in cells of human origin. In irradiated mice MVA did not elicit any morbidity or lethality even when administered at high doses intra-cerebrally [39]. From 1972 until 1980 (the end of compulsory smallpox vaccination) MVA was licensed in Germany [40] and was included in the official immunisation schedule [41]. In a large field study carried out in Germany over 120,000 previously unvaccinated individuals were vaccinated with MVA (0.2 mL) administered either intra-dermally or subcutaneously. The study population included high-risk groups (e.g. people suffering from allergies, elderly people, alcoholics) [39]. MVA proved to be non-contagious and avirulent. Viral replication is blocked late during infection of cells but importantly viral and recombinant protein synthesis is unimpaired even during this abortive infection. Replication-deficient recombinant MVA has been viewed as an exceptionally safe viral vector.

AdCh63 vector
Adenoviruses are attractive vectors for human vaccination. They possess a genetically stable virion so that inserts of foreign genes are not deleted. Adenoviruses can infect large numbers of cells without any evidence of insertional mutagenesis. Previous mass vaccination campaigns in very large numbers of US military personnel using orally administered live human adenovirus serotype 4 and 7 have shown good safety and efficacy data [42]. 201 healthy volunteers have to date received the AdCh63 vector with no serious adverse events, and a further 13 have received AdCh63 ME-TRAP mixed with MVA ME-TRAP with no serious adverse events.

The most widely studied recombinant adenovirus vector is the human adenovirus AdHu5. However, the ubiquity of human adenovirus infections can generate host anti-vector immunity that may limit the utility of this vector. Depending on the geographical region, between 45 and 80 % of adults carry AdHu5-neutralising antibodies [43]. Immunisation with AdHu vectors in animal models in the presence of pre-exposure to human adenoviruses attenuates responses to the vaccine [44-46]. Phase I trials of a multiclade HIV-1 vaccine delivered by a replication defective AdHu5 have previously excluded volunteers with pre-existing antibodies to AdHu5 at titres greater than 1:12 [47]. Higher antibody titres attenuate immunogenicity, although they do not result in higher reactogenicity [48].

The prevalence of immunity to human adenoviruses prompted the consideration of simian adenoviruses as vectors. They exhibit hexon structures homologous to that of human adenoviruses. Simian adenoviruses are not known to cause pathological illness in humans and the prevalence of antibodies to chimpanzee origin adenoviruses is less than 5% in humans residing in the US [49]. In a recent study in Kenya, 23% of children (aged 1-6 years) had high-titre neutralising antibodies to AdHu5, whilst only 4% had high-titre neutralising antibodies to AdCh63 [50].

There is no available or validated *in vitro* cell co-culture method to examine co-infection with human and simian adenovirus vectors as the latter are non-replicating. Due to a lack of any sequence homology

between the replication-deficient AdCh63 and MVA vectors, complementation of MVA by AdCh63 does not occur. Pre-clinical bioavailability studies have demonstrated no persistence of the AdCh63 vector 24 hours post intramuscular administration. Therefore, residual priming AdCh63 vector is very unlikely to be present at the time of administration of a MVA boost, 8 weeks later.

The ME-TRAP insert
The polypeptide encoded consists of a series of known CTL epitopes from *Plasmodium falciparum* pre-erythrocytic stage antigens [51] fused to a complete pre-erythrocytic stage antigen, Thrombospondin Related Adhesion Protein (TRAP) [52]. The individual CTL epitopes are recognised by a number of common human HLA types, represent a variety (six) of potentially protective target antigens and are included to ensure an immune response to the vaccine in the majority of the population vaccinated [53]. TRAP is an abundant pre-erythrocytic stage antigen. Human volunteers immunised with irradiated sporozoites and protected against malaria develop T cell responses against TRAP making it a strong candidate for inclusion in a malaria vaccine [54]. Viral vectors containing the CS antigen used in the RTS,S vaccines are much less immunogenic than TRAP, and were not protective in sporozoite challenge studies [55].

Laboratory assays
When assessing immunogenicity we will use the gamma-interferon enzyme-linked immunospot (ELISPOT) assay in two forms; the ex-vivo form (which has correlated directly with protection in two mouse models of malaria) [56], and the short-term culture form(which has correlated with protection in the field trial of RTS,S/AS02 in the Gambia [57] and in sporozoite challenge studies of viral vector vaccinations in Oxford [58]. We will assay with pools of 20-mer peptides. The ELISPOT enumerates T cells in volunteers' peripheral blood which secrete gamma-interferon on contact with an epitope from the construct. Gamma-interferon secreted by T cells after interaction with infected liver cells has been shown to induce death of liver-stage parasites[25]. We will also use flow cytometry studies to examine the $CD8^+$ IFN-γ T cell population that correlated with protection in the recent sporozoite challenge studies described above, depending on the availability of cells.

2.2. Rationale

The purpose of this trial is to assess the safety and immunogenicity of these promising candidate vaccines in healthy children and adult volunteers in a malaria endemic region. The regimen proposed here has protected non-immune volunteers in Oxford against sporozoite challenge, and so may be protective against naturally acquired infection in The Gambia.

A parallel study is planned to assess the safety and immunogenicity in healthy adult volunteers in Kenya, with subsequent safety and efficacy studies in Kenyan infants in due course (see vaccine development plan).

We will conduct a sero-survey of approximately 30 stored samples from healthy Gambian adults to determine the adult prevalence of neutralising antibodies (NA) to the AdCh63 vector in Gambians. We anticipate that the seroprevalence will be very low as it has been in all studies to date including UK, Italian and Kenyan subjects. We would expect the prevalence of anti-vector antibodies to increase a little with age, and so we propose to screen adults rather than children. This survey will be conducted prior to dosing but not prior to study commencement. An adult prevalence of <30% (defined as NA titre of ≤ 1:200) would allow the trial site to be considered suitable.

GROUP 1:
The study population will initially comprise of 16 healthy adult males aged 18-50 years (Group 1). Although female volunteers have received these vaccines in Oxford, it is conventional for phase I studies of new vaccines in a new population to occur first in adult males to avoid the theoretical risk to foetuses which cannot be fully excluded by pregnancy testing and contraception during the course of the trial.

We will examine the safety and immunogenicity of the vaccination regimen found to be protective in Oxford (i.e. AdCh63 ME-TRAP prime followed by MVA ME-TRAP boost). MVA ME-TRAP has been extensively used in clinical trials (including sites in Kenya and The Gambia), and so we propose to begin with the full dose. AdCh63 ME-TRAP has not been used before in The Gambia, and so we propose to begin with one fifth of the dose used in Oxford in 6 volunteers before increasing to the full dose. AdCh63 ME-TRAP has previously been delivered by intramuscular (IM) injection and MVA ME-TRAP has usually been given by intradermal (ID) injection. However, MVA can be safely delivered by the intramuscular route [55] and IM MVA ME-TRAP is under review in Oxford. As IM would be the preferred route of administration for vaccinations in children, we propose to administer all vaccines by the IM route.

We do not propose to include a placebo group for adult volunteers. At this stage our objective is to describe the safety profile in a small number of individuals, and the confidence intervals for the proportion of individuals with a particular event would be too wide for meaningful comparison with a placebo group. Immunogenicity will be judged by comparison with baseline.

GROUPS 2&3:

Following analysis of data from group 1, in the event of:

1. Acceptable safety and tolerability (ie demonstration of a similar adverse event and safety profile to that seen in healthy adult volunteers to date and favourable review by the DSMB) and
2. Acceptable immunogenicity (a mean of ≥ than 500 antigen specific SFU/million PBMCs observed post boost)

we will plan to subsequently assess safety and immunogenicity in 24 healthy children aged 2-6 years (Groups 2-3) compared to 12 healthy controls (aged 2-6 years). AdCh63 ME-TRAP has not been administered to children before, and so we propose to begin with one fifth of the dose used in Oxford in adult volunteers (group 2) before increasing to the full dose of AdCh63 ME-TRAP (group 3). It is anticipated that the full dose of 5×10^{10} vp AdCh63 ME-TRAP will be tolerated in children aged 2-6.

MVA ME-TRAP has been administered to children previously and well tolerated at a dose of 1.5×10^{8} pfu.[32] It is anticipated that 2×10^{8} pfu MVA ME-TRAP will also be well tolerated. However, a lower dose of 1×10^{8} pfu MVA ME-TRAP may be as immunogenic but less reactogenic than 1.5 or 2×10^{8} pfu. For this reason, in each group 2 and 3, vaccinated children will be randomised to receive either 1×10^{8} pfu MVA ME-TRAP or 2×10^{8} pfu MVA ME-TRAP.

MVA ME-TRAP has previously been administered by the intradermal (ID) route in children.[32] However, MVA can be safely delivered by the intramuscular route [55] and IM MVA ME-TRAP has been safety administered intramuscularly to adults in Oxford, The Gambia & Kenya (Duncan et al unpublished). AdCh63 ME-TRAP has been safety administered intramuscularly in adults with reduced numbers of local adverse events compared to the intradermal route (O'Hara et al unpublished). The IM route is the preferred route of administration for vaccinations in children, so we propose to administer all vaccines in groups 2 & 3 by the IM route.

It is anticipated that there will a considerable rate of concurrent diseases in this paediatric population. This, combined with the known difficulties assessing adverse events in young children support the planned inclusion of paediatric control groups to aid in the objective assessment of the relationship of adverse events to vaccination. The comparator vaccine (human diploid cell rabies vaccine) has a good safety and tolerability profile, has no malaria protective effects, and is not routinely administered in the Extended Programme of Immmunization (EPI). It has been widely used as a comparator vaccine in early phase malaria vaccine trials in Africa. At the end of the trial, the PI will arrange for all trial participants to receive a standard schedule of HDCRV.

Vaccine development plan
A correlate of immunity has been identified in the recent challenge studies and this makes immunogenicity studies at Phase I particularly informative. In previous studies we found attenuated immunogenicity in volunteers from The Gambia and Kenya which may have been due to previous exposure to malaria [28]. Phase I immunogenicity data from adults and children in The Gambia gathered early in vaccine development would therefore provide key information to aid optimisation of the vaccine regime before larger efficacy trials in the field.

If the safety and immunogenicity data that we observe in this and the comparable study in Kenya are favourable, and the results of the ongoing sporozoite challenge studies in Oxford are encouraging, we plan to proceed to a Phase IIb trial in young children in Africa in 2011.

Potential subjects with HIV
Safety data for MVA is available from HIV infected adults in Germany [59] and New York [60], and for MVA ME-TRAP in adults in Kenya [30], and for 400 children in Kenya of whom 1% were probably HIV positive [32]. HIV is not reported as a risk factor for human adenoviral infection [61]. Furthermore, since AdCh63 is replication deficient in human cells, there will be no further viral replication in the human host, irrespective of immunodeficiency. For these reasons we anticipate that these vaccines will be safe for use in the HIV positive population. Indeed, the need for an HIV test before immunisation would preclude widespread delivery of a successful immunisation, and it is clearly desirable to protect HIV infected individuals as well as uninfected. However, since this is first trial of AdCh63 ME-TRAP and MVA ME-TRAP in Africa and it is standard practice to obtain safety data in healthy subjects before immunising those with HIV, we will screen for and exclude HIV positive volunteers and plan to include HIV positive volunteers in clinical trials at a later development stage.

2.3. Risks and Benefits

2.3.1. Potential Risks

The general risks to participants are associated with blood sampling and vaccination. The volume of blood drawn over the study period should not compromise these otherwise healthy volunteers. Potential risks from vaccination include local and systemic reactions, which are described below. It is expected that the side effect profile of AdCh63 ME-TRAP will be similar to that seen amongst healthy volunteers in Oxford. MVA ME-TRAP has been previously studied in healthy adults in Kenya and Gambia and in children in Kenya. A detailed summary of the adverse event profile of AdCh63 ME-TRAP and MVA ME-TRAP at the doses proposed is included in the IB for each IMP. There have been no vaccine-related serious adverse events with either malaria vaccine. Full information on the licensed human diploid cell rabies vaccine can be found in the summary of product characteristics (SPC).

Local reactions
Mild tenderness, bruising, light-headedness, or rarely vasovagal syncope may result from venepuncture.
Vaccination usually provokes a local inflammatory reaction.

MVA ME-TRAP

Intradermal (for information only, not to be used in this study)

This description covers the expected local reactions associated with intradermal administration of MVA ME-TRAP from previous trials in heterogenous populations of malaria-naive and malaria-exposed adults and children.

Redness or skin discolouration occurs at the vaccine site in most vaccinees (77% to 100%) following intradermal MVA ME-TRAP (range 5 – 21 mm in early UK trials using 3 x 10^7 pfu to a mean discolouration of 88mm in Kenyan trials using 1.5 x 10^8 pfu). In Caucasian skin, this discolouration typically manifests as a 5mm central red area with a paler pink surrounding area that ranges in size from about 1 –7cm in diameter and peaks at 48 hours post vaccination. In African skin the erythema is less obvious and instead a discolouration is apparent with similar sizing. It is generally apparent within the first 24 hours post vaccination and has resolved within 4 weeks of vaccination.

Induration occurs at the vaccine site in the majority of volunteers (75% to 100%), again in a dose related manner. Induration is evident by thickening of the skin and peaks within 72 hours of vaccination, again being absent at resolving by 4 weeks post vaccination.

At higher doses of MVA ME-TRAP administered intradermally, (e.g. 1.5 or 2 x 10^8 pfu), blistering can occur and on occasion these blisters can become deroofed. In previous trials this has occurred at a frequency between 2% and 20% with blister size ranging between 20 – 28 mm.

The majority of vaccinees report discomfort at the site of vaccination which is short lived, resolving within 24-48 hours. For a small number of vaccinees the discomfort affects activities of daily living and is classified as moderate or, on occasion, severe in nature.

Other injection site effects include itching and scaling which occur in 20-25% of vaccinees, peaking at 48 – 72 hours and resolving by 4 weeks post vaccination.

Intramuscular

To date, there has been a significant reduction in the frequency and duration of injection-site reactions when IM MVA ME-TRAP has been compared to ID MVA ME-TRAP in malaria-naive and malaria-exposed adults (O'Hara et al, unpublished). This has also been observed with other MVA vector vaccines [55]. Injection site pain is the commonest local reaction, and usually resolves within 48 hours. Other local reactions, such as redness/discolouration and swelling, are observed much less frequently and for a shorter duration than with the ID route (O'Hara et al, unpublished).

AdCh63 ME-TRAP

To date 85 healthy volunteers have received AdCh63 ME-TRAP alone, and five in repeat immunisation schedules. Injection site discomfort occurs frequently. Less frequent adverse reactions include erythema, swelling, itching and warmth. Local adverse reactions are mild in nature in the majority of cases and resolve rapidly (within 48 hours).

During the manufacturing process of AdCh63 ME-TRAP a biocide named Kathon will be used. Kathon is added to body washes, conditioners, liquid soaps, shampoos and wipes as a preservative. The maximum dose is 0.1% for 'rinse off' products and for 'leave on' products it is 0.05%. It has been approved by regulatory authorities throughout the world as a preservative in these products. As a skin sensitiser it is known to cause contact dermatitis. An internal study SOP/P22 was set up by Clinical Biomanufacturing Facility (CBF) to quantify the levels of Kathon that were removed during the final purification step of buffer exchange during the manufacture of the vaccine. This study utilized high performance liquid chromatography and showed that trace amounts of Kathon may be left on the desalting column after carrying out the rinse and sanitisation steps. However, the study confirmed greater than 99.9975% removal

of Kathon to approximately 30 fold less than the limits for 'leave on' products containing Kathon. We will exclude anyone from the study with a history of clinically significant contact dermatitis or sensitivity to Kathon.

HDCRV
Mild and self-limited local reactions such as pain at the site of injection, redness and swelling occur in 21–74% of vaccinees. Bleomicin and neomycin are used in the manufacturing process and therefore volunteers with a history of hypersensitivity to these drugs, or aminoglycoside antibiotics, will be excluded from the trial.

Systemic Reactions
A proportion of volunteers may report a transient mild 'flu-like illness within 24 hours of vaccination which resolves rapidly.

MVA ME-TRAP
Systemic effects can occur with MVA ME-TRAP and are dose related, with symptoms occurring at a higher frequency and greater intensity in volunteers receiving higher doses. At higher doses (1.5 - 2 × 10^8 pfu) the majority of subjects experience a mild flu-like illness (symptoms of malaise (up to 87.5%), myalgia (up to 75%) and feverishness (up to 75%) after the first dose of MVA ME-TRAP that is short-lived (12 to 24 hours). This occurs within 24 hours of vaccination and is less frequent with lower doses (<15% of immunisations) and second or third MVA immunisations. Objective fever occurs in up to 37.5% of vaccinees.

AdCh63 ME-TRAP
Similar mild 'flu-like symptoms can occur with AdCh63 ME-TRAP. These symptoms occur in 28/39 of volunteers at 5 x 10^{10}vp intramuscularly. The frequency of 'flu-like symptoms increases with use of the 2 x 10^{11}vp dose to 9/10. Objective fever has not been observed in 46 males in Africa, and has occurred in 26/85 volunteers in the UK (at doses up to 2 x 10^{11} vp).

HDCRV
Mild systemic reactions such as fever, headache, dizziness and gastrointestinal symptoms occur in 5–40%, and systemic hypersensitivity following booster injections in 6% of vaccinees, but are less common following primary immunization.

With any vaccine, serious allergic reactions including anaphylaxis may occur. The exact risk of anaphylaxis is difficult to quantify, and is estimated at approximately 1 in 10^5 to 10^6. Volunteers will be vaccinated in a clinical area where Advanced Life Support drugs and equipment are immediately available for the management of serious adverse reactions.

Prophylactic use of paracetamol or non-steroidal anti-inflammatory drugs is not required. Parents of study subjects will be provided with paracetamol and they will be advised to use this drug to treat local or systemic adverse events as they see fit should these develop post-immunisation, but not prophylactically.

2.3.2. Known Potential Benefits

There are no known benefits to volunteers for participation in this phase I study. All children enrolled will receive a full course of rabies vaccine (HDCRV) at the end of the trial, this will be arranged by the PI, Dr K Bojang.

3. OBJECTIVES

3.1. Primary Objective

To assess the safety of a heterologous prime-boost vaccine strategy with AdCh63 ME-TRAP and MVA ME-TRAP in healthy adults and children in The Gambia.

3.2. Secondary Objectives

To assess the immunogenicity of a heterologous prime-boost vaccine strategy with AdCh63 ME-TRAP and MVA ME-TRAP in healthy adults and children in The Gambia.

3.3 Tertiary Objective

To compare the immunogenicity of two doses of MVA ME-TRAP in children.

4. STUDY DESIGN

4.1. Description and Justification of Study Design

This is a single-blinded controlled dose escalation phase I study. The trial period will be approximately 12 months.

The sample size for phase I studies balances the need to avoid exposing a large group to an unknown risk with the need for data from an adequate sample. The aim is not to provide pre-marketing safety data but to justify the future study of larger groups. The sample size was determined by the requirement to make a preliminary evaluation of inter-group variability to avoid excessive risk. Further justification of the study design is contained in Section 2.2. The addition of a control group to the groups involving children aged 2-6 (Groups 2 and 3) is to control for the anticipated high frequency of concurrent diseases in this population and aid in the objective assessment of the relationship of adverse events to vaccination. There may be benefit to the malaria vaccinated volunteers in terms of reduced susceptibility to malaria although this trial is not designed to assess this possibility, and there is no evidence that this is the case in this population at present since the vaccine has not been tested. A comparator vaccine, rather than saline placebo will be used for the control groups. The comparator vaccine will be the HDCRV. Although this will be administered in a 0, 2 month regimen, there is evidence from the SPC that two vaccines may be immunogenic and may provide some efficacy against rabies. To avoid potential inequality, all volunteers will receive a full standard schedule of HDCRV at the end of the trial.

The control group volunteers will also undergo venesection for safety laboratory testing, and will undergo ex-vivo ELISPOT immunology analysis exactly as planned for the vaccinated volunteers, to control for TRAP-specific T cell responses which may develop during the study period as a result of natural exposure to *P.falciparum*.

Allocation bias will be reduced by randomised allocation to study groups. In addition, single-blinding will be employed to reduce bias in reporting of adverse events. Given that the crucial safety assessment for these viral vectored vaccines occurs in the first 72 hours (when the majority of vaccine-related adverse events are

reported) the fieldworkers conducting the early safety assessments during this period will be blinded to vaccine allocation, as will the volunteer and their carer. Should the fieldworker have a significant safety concern the trial clinician (who will not be blinded to group allocation) will review the volunteer.

4.2. Study groups

Study groups are as follows: (All injections are administered by the intramuscular route – IM).

Group 1A (6 healthy male volunteers aged 18-50 years):
AdCh63 ME-TRAP 1 x 10^{10}vp IM followed by MVA ME-TRAP 2 x 10^{8} pfu IM 8 weeks later

Group 1B (10 healthy male volunteers aged 18-50 years):
AdCh63 ME-TRAP 5 x 10^{10}vp IM followed by MVA ME-TRAP 2 x 10^{8} pfu IM 8 weeks later

Group 2A (6 healthy children aged 2-6 years):
AdCh63 ME-TRAP 1 x 10^{10}vp IM followed by MVA ME-TRAP 1 x 10^{8} pfu IM 8 weeks later

Group 2B (6 healthy children aged 2-6 years):
AdCh63 ME-TRAP 1 x 10^{10}vp IM followed by MVA ME-TRAP 2 x 10^{8} pfu IM 8 weeks later

Group 2C (6 healthy children aged 2-6 years): Control
HDCRV 1ml IM followed by HDCRV 1ml IM 8 weeks later

Group 3A (6 healthy children aged 2-6 years):
AdCh63 ME-TRAP 5 x 10^{10}vp IM followed by MVA ME-TRAP 1 x 10^{8} pfu IM 8 weeks later

Group 3B (6 healthy children aged 2-6 years):
AdCh63 ME-TRAP 5 x 10^{10}vp IM followed by MVA ME-TRAP 2 x 10^{8} pfu IM 8 weeks later

Group 3C (6 healthy children aged 2-6 years): Control
HDCRV 1ml IM followed by HDCRV 1ml IM 8 weeks later

Duration of study involvement, clinic visits and blood sampling regimes are identical for all volunteers in each study group. There will be a total of 7 clinic attendances, 7 blood sampling occasions and two vaccination visits for each volunteer. Total blood volume for the study period (300 days from first vaccination for all volunteers) will be 190 mLs for group 1 and 35mls for groups 2 & 3.

4.3. Endpoints

4.3.1. Primary Endpoint

All solicited and unsolicited local and general vaccine-linked adverse events (AEs) including clinically significant laboratory abnormalities.

4.3.2. Secondary & Tertiary Endpoints

Measures of immunogenicity will include, where practicable:

Ex vivo ELISPOT responses to overlapping pools of ME-TRAP peptides
Cultured ELISOPT responses to overlapping pools of ME-TRAP peptides
ICS by flow cytometry
TRAP antibody ELISA

© University of Oxford 2009

Although not a safety or immunogenicity endpoint, neutralising antibody titre against AdCh63 will be measured to establish if high-titre neutralising antibodies against the AdCh63 vector correlate with measures of immunogenicity.

Although not a safety or immunogenicity endpoint, polymerase chain reaction (PCR) testing for *Plasmodium falciparum* will be performed on blood taken at the first vaccination visit to facilitate interpretation of immunogenicity data in the context of possible natural exposure to malaria. This PCR assay for *P. falciparum* is still under development and is not validated for clinical diagnostic purposes.

As a tertiary endpoint, a comparison of the immunogenicity of two doses of MVA ME-TRAP will be made.

4.4. *Safety*

4.4.1. Safety Monitoring

Safety oversight will be the responsibility of the investigators, the independent Local Safety Monitor (LSM) and the independent Data Safety and Monitoring Board (DSMB).

Safety monitoring for Group 1

The dose escalation of AdCh63 ME-TRAP to 5 x 10^{10}vp between groups 1A and 1B will only occur following satisfactory safety review by the DSMB.

Safety monitoring for Groups 2 and 3

As detailed in the schedule below, administration of AdCh63 ME-TRAP will occur in three stages. Approval from the Chair of the Gambia Government/MRC Laboratories Ethics Committee and DSM following submission of 14-day safety profiles following stage 1a will be required to proceed to stage 2a. Approval from the Chair of the Gambia Government/MRC Joint Ethics Committee following submission of 14-day safety profiles to the DSMB following stage 2a will be required to proceed to stage 3a.

Stage 1a

**Group 2A**	AdCh63 ME-TRAP 1 x 10^{10}vp IM
**Group 2C**	HDCRV 1ml IM

↓ 1. Submission of 14 day safety profile to DSMB & Chair of Ethics Committee
2. Approval from the Chair of the Ethics Committee & DSMB

Stage 2a

**Group 2B**	AdCh63 ME-TRAP 1 x 10^{10}vp IM
**Group 3A**	AdCh63 ME-TRAP 5 x 10^{10}vp IM

↓ 1. Submission of 14 day safety profile to DSMB
2. Approval from the Chair of the Ethics Committee & DSMB

Stage 3a

**Group 3B**	AdCh63 ME-TRAP 5 x 10^{10}vp IM
**Group 3C**	HDCRV 1ml IM

As detailed in the schedule below, administration of MVA ME-TRAP will occur in three stages. Approval from the Chair of the Ethics Committee & DSMB following submission of 14-day safety profiles to the DSMB & Ethics Committee following stage 1b will be required to proceed to stage 2b. Approval from the Chair of the Ethics Committee & DSMB following submission of 14-day safety profiles to the DSMB & Ethics Committee following stage 2b will be required to proceed to stage 3b.

**Group 2A**	MVA ME-TRAP 1 x 10^8 pfu IM
**Group 2C**	HDCRV 1ml IM

Stage 1b

1. Submission of 14 day safety profile to DSMB and Chair of Ethics Committee
2. Approval from the Chair of the Ethics Committee & DSMB

Stage 2b

**Group 2B**	MVA ME-TRAP 2 x 10^8 pfu IM
**Group 3A**	MVA ME-TRAP 1 x 10^8 pfu IM

1. Submission of 14 day safety profile to DSMB & Chair of Ethics Committee
2. Approval from the Chair of the Local Ethics Committee

Stage 3b

**Group 3B**	MVA ME-TRAP 2 x 10^8 pfu IM
**Group 3C**	HDCRV 1ml IM

4.4.2. Discontinuation of the Study

Both arms of the study will be discontinued in the event of any of the following:

- New scientific information is published to indicate that volunteers in the study are being exposed to undue risks as a result of administration of the IMPs by any route of administration, or as a result of the follow-up schedule.
- Serious concerns about the safety of the IMPs arise as a result of one or more vaccine related SAE occurring in the subjects enrolled in this or any other ongoing study of the IMPs.
- For any other reason at the discretion of the Principal Investigator.

4.5. Data Collection

Adverse events will be documented in individual case report forms (CRFs) for each volunteer. They will be recorded under two headings; local and general. There will be separate sections for concomitant medication, concomitant vaccination, non-serious adverse event documentation, serious adverse event documentation and study conclusion. Any deviations from the study protocol will be documented (see section 12.6). Case report forms will be kept securely, and HIV status will not be recorded in the CRF.

5. Selection and Withdrawal of Subjects

5.1. Inclusion Criteria

Consenting adult males aged 18-50 years in good health and healthy children aged 2-6 years.with consenting parents.

5.2. Exclusion Criteria

Any of the following constitute an exclusion criterion:

- Clinically significant history of skin disorder (psoriasis, contact dermatitis etc.), allergy, symptomatic immunodeficiency, cardiovascular disease, respiratory disease, endocrine disorder, liver disease, renal disease, gastrointestinal disease, neurological illness.
- Severe malnutrition.
- Hypersensitivity to HDCRV.
- History of allergic disease or reactions likely to be exacerbated by any component of the vaccines, e.g. egg products, Kathon, neomycin, betapropiolactone.
- History of splenectomy
- Haemoglobin less than 9.0 g/dL, where judged to be clinically significant in the opinion of the investigator
- Serum Creatinine concentration greater than 70 μmol/L, where judged to be clinically significant in the opinion of the investigator
- Serum ALT concentration greater than 45 U/L, where judged to be clinically significant in the opinion of the investigator
- Blood transfusion within one month of enrolment.
- History of vaccination with previous experimental malaria vaccines.
- Administration of any other vaccine or immunoglobulin within two weeks before vaccination.
- Current participation in another clinical trial, or within 12 weeks of this study.
- Any other finding which in the opinion of the investigators would increase the risk of an adverse outcome from participation in the trial.
- Likelihood of travel away from the study area.
- HIV positive.
- Positive malaria antigen test

5.3. Withdrawal Criteria

Subjects may be withdrawn from the study:

- By withdrawing consent
- On the decision of the Investigator
- On the advice of the DSMB

The Investigator may withdraw the subject for any of the following reasons:

- Any adverse event which results in inability to comply with study procedures
- Ineligibility either arising during the study or retrospectively (having been overlooked at screening)
- Significant protocol deviation

- Loss to follow up (applies to a subject who consistently does not return for protocol study visits, is not reachable by telephone or other means of communication and/or is not able to be located)

The reason for withdrawal will be recorded in the CRF. If the subject is withdrawn due to an AE, the Principal Investigator will arrange for appropriate specialist management or follow up visits until the AE has resolved or stabilised. The regulatory authorities will be informed in a timely manner according to recognised guidelines. The extent of follow up after premature discontinuation will be determined by the Investigator but will be at least for the whole study period. If possible within the study period, volunteers will be replaced.

6. RANDOMISATION

Adult volunteers in group 1 will be allocated to study groups by investigators (i.e. not randomised).
Children in groups 2 and 3 will be randomised to groups A, B or C. Randomisation of subjects will done by an independent statistician at the Centre for Statistics in Medicine, Oxford using stratified randomisation (stratified on age - 2 categories, split by the median value of those recruited). As all volunteers will be recruited and screened before randomisation, the allocation list will be prepared after the final patient has been screened. The statistician carrying out the randomisation will have no knowledge of the participants, with the exception of age, as this is required for the stratification.

7. BLINDING

For groups 2 and 3, the investigators and the vaccinators will be un-blinded to the group allocations. However, the children's parents/carers and field worker assessing adverse events in the 3 days immediately following vaccination will be blinded to the group allocation.

8. INVESTIGATIONAL MEDICINAL PRODUCT

8.1. Description

MVA ME-TRAP is manufactured under Good Manufacturing Practice conditions by Impfstoffwerk Dessau-Tornau (IDT), Germany. MVA ME-TRAP is supplied as a liquid. The vaccine suspension is supplied as sterile 0.2 mL aliquots in 1.0 mL clear glass injection vials.

AdCh63 ME-TRAP is manufactured under Good Manufacturing Practice conditions by the Clinical Biomanufacturing Facility (CBF), Churchill Hospital, Oxford. The virus suspension is supplied as sterile 0.6 mL aliquots in glass vials.

HDCRV is manufactured by Sanofi Pasteur MSD Ltd. It is supplied as a dry pinkish beige to orangey yellow dry powder for reconstitution in a solvent (water for injection, a clear, colourless solution).

8.2. Formulation

MVA ME-TRAP will be provided in lots of vials of 200 µL or 450 µL volume in 10 mM Tris buffer. The dose of MVA ME-TRAP to be used in this study is 1 or 2 × 10^8 pfu.

Each vial of AdCh63 ME-TRAP contains a concentration of 1.35 x 10^{11} vp/ml in 10 mM Histidene, 35 mM NaCl. The doses of AdCh63 ME-TRAP to be used in this study will be 1 x 10^{10} vp (Groups 1A, 2A & 2B) and 5 x 10^{10} vp (Groups 1B, 2B, 3A & 3B).

After reconstitution the HDCRV is 1ml, (inactivated, strain PM/WI 38 1503-3M) ≥ 2.5 IU. Excipients are human albumin solution and water for injection (1ml). The dose will be 1ml.

8.3. Product Storage

The AdCh63 ME-TRAP and MVA ME-TRAP vials are stored between –70^{0}C and –90^{0}C, in a locked freezer at the University of Oxford, Churchill Hospital. All movements of the study vaccines between IDT or CBF and the University of Oxford and between the locked freezer and clinic room will be documented. The vaccines will be shipped from Oxford on dry ice, then stored in a –70^{0}C freezer at the MRC unit until required.
HDCRV will be stored between +2^{0}C and +8^{0}C. Records of vaccine storage will be documented in a vaccine accountability log.

8.4. Dispensing and Administration

Malaria vaccines will be transported to Sukuta Health Centre on dry ice on the morning of use. They will be thawed at Sukuta Health Centre and kept in a cold box, and used within 4 hours of thawing. HDCRV will be removed from a refrigerator and kept in a cool-box until used.

The following constitute contraindications to administration of vaccine at that point in time; if any one of these occur at the time scheduled for vaccination, the subject may be vaccinated at a later date, or withdrawn at the discretion of the Principal Investigator. Medical care including inpatient care if necessary will be offered.

- Acute disease at the time of vaccination (Acute disease is defined as the presence of a moderate or severe illness with or without fever) All vaccines can be administered to persons with a minor illness such as mild diarrhoea, mild upper respiratory infection with or without low-grade febrile illness, *i.e.* axillary temperature of <37.5°C (99.5°F). Details of any minor illness will be recorded in the CRF.
- Axillary temperature of ≥37.5°C (99.5°F) at the time of vaccination.

AdCh63 ME-TRAP and MVA ME-TRAP will be administered intramuscularly according to standard operating procedures (SOP). Each volunteer will be monitored for one hour (or longer if necessary) after each vaccination. Resuscitation (including intubation) equipment and medication will be available in the clinic site and a clinician trained in resuscitation will be present at all times.

AdCh63 ME-TRAP and MVA ME-TRAP are genetically modified organisms. In order to minimise dissemination of the recombinant vectored vaccine viruses into the environment, the inoculation site will be covered with a dressing after immunisation. This should absorb any virus that may leak out through the needle track, and will be removed from the injection site after 30 minutes. Vaccine administrators will follow precautions for the safe handling of GMOs (including the use of eye protection and gloves).

HDCRV 1ml will be administered using an identical type of needle and syringe to those used to administer malaria vaccines. In order to ensure adequate blinding of the volunteer and their carer, the same personal protective equipment will be worn by the vaccinator, as described above.

8.5. Trial Regimen

There will be an initial dose escalation in adults and children to confirm safety of a lower dose of AdCh63 ME-TRAP before using the full dose.

Group 1:

Volunteers in group 1B will not be vaccinated until a minimum of 14 days have elapsed following vaccination of volunteers in group 1A, following safety review by the LSM and DSMB.

To avoid a previously unsuspected AE affecting multiple volunteers, one volunteer in group 1A will be vaccinated alone. 48 hours later a further two volunteers will be vaccinated. This is to allow safety assessment by Investigators. In the absence of significant safety concerns affecting these initial volunteers at 48 hours post-vaccination, the remaining volunteers in this group will be vaccinated.

Groups 2 and 3:

Vaccination of volunteers in groups 2 & 3 will not begin until a minimum of 14 days have elapsed following vaccination of volunteers in group 1B, and a safety review of adverse event data for groups 1A & 1B has been reviewed by the LSM & DSMB.

The following flow chart outlines the regimen for vaccination of volunteers in groups 2 and 3.

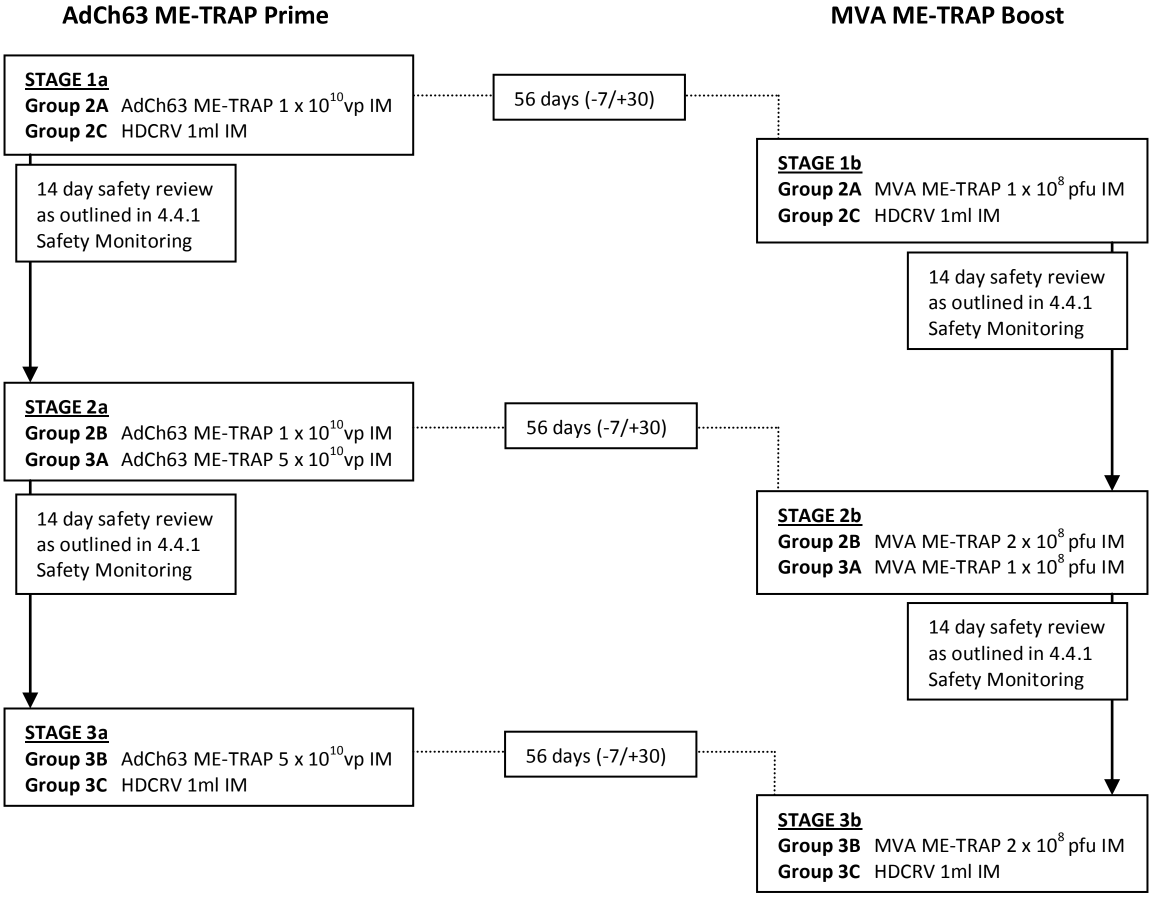

In stage 1a, two volunteers from group 2A and one control volunteer from group 2C will be immunised simultaneously, with AdCh63 ME-TRAP 1 x 10^{10} vp and HDCRV, respectively. The remaining volunteers in groups 2A and 2C will be vaccinated after 48 hours have elapsed, providing there are no safety concerns affecting these initial volunteers.

Stage 2a will not proceed until approval is granted following 14 day safety monitoring following stage 1a. One volunteer from group 2B and two volunteers from group 3A will be immunised simultaneously, with AdCh63 ME-TRAP 1 x 10^{10} vp and AdCh63 ME-TRAP 5 x 10^{10} vp, respectively. The remaining volunteers in groups 2B and 3A will be vaccinated after 48 hours have elapsed, providing there are no safety concerns affecting these initial volunteers.

Stage 3a will not proceed until approval is granted following 14 day safety monitoring following stage 2a. All volunteers in groups 3B and 3C may be vaccinated simultaneously.

In stage 1b, two volunteers from group 2A and one control volunteer from group 2C will be immunised simultaneously, with MVA ME-TRAP 1 x 10^8 pfu and HDCRV, respectively. The remaining volunteers in groups 2A and 2C will be vaccinated after 48 hours have elapsed, providing there are no safety concerns affecting these initial volunteers.

Stage 2b will not proceed until approval is granted following 14 day safety monitoring following stage 1b. One volunteer from group 2B and two volunteers from group 3A will be immunised simultaneously, with MVA ME-TRAP 2 x 10^8 pfu and MVA ME-TRAP 1 x 10^8 pfu, respectively. The remaining volunteers in groups 2B and 3A will be vaccinated after 48 hours have elapsed, providing there are no safety concerns affecting these initial volunteers.

Stage 3b will not proceed until approval is granted following 14 day safety monitoring following stage 2b. All volunteers in groups 3B and 3C may be vaccinated simultaneously.
Results of the FBC, creatinine and ALT will be reviewed before any immunisation is administered.
Results of blood tests will be communicated to volunteers/their carers on their next clinic visit.

8.6. Labeling and Packaging

Example vial labels (these are included for example only and are subject to change):

CLINICAL TRIAL: VAC041<br>**MVA ME-TRAP VACCINE XX mL**<br>Vial contains XXx 10^8 pfu/mL<br>Vial No: _____ For Intramuscular Injection<br>Batch no: XXXXXX Store at - 70°C<br>Volunteer no: _____ Expiry Date: ____		**FOR CLINICAL TRIAL USE ONLY**<br>Sponsor: University of Oxford<br>CCVTM, Old Road, Oxford. OX3 7LJ<br>Tel: 01865 857382<br>Fax: 01865 857471
CLINICAL TRIAL: VAC041<br>**AdCh63 ME-TRAP VACCINE XX ml**<br>Vial contains XXX x 10^{11} vp/ml<br>Vial No: _____ For Intramuscular Injection<br>Batch no: XXXX, Fill XXX Store at - 70°C<br>Volunteer no: _____ Expiry Date: ____		**FOR CLINICAL TRIAL USE ONLY**<br>Sponsor: University of Oxford<br>CCVTM, Old Road, Oxford. OX3 7LJ<br>Tel: 01865 857382<br>Fax: 01865 857471

8.7. Accountability

All movements of vaccines will be documented in vaccine accountability logs according to local site SOPs.

8.8. Revaccination exclusion criteria

Anaphylactic reaction following administration of vaccine constitutes an absolute contraindication to further administration of vaccine. If this occurs during the study, the subject will be withdrawn and followed until resolution of the event, as with any serious adverse event.

9. Study Schedule

9.1. Screening

The MRC study team will hold local community meetings and explain the study to potential participants and the carers of potentially eligible children. During these meetings the investigators will explain the following: the need for a vaccine (including a simple picture of the burden of malaria on the community); the current status of vaccine development (including the fact that this is likely to be a prolonged process); the study screening and informed consent procedure; risks of vaccination and the unproven benefits of vaccination. It will be stressed that this is an experimental vaccine and cannot be guaranteed to provide protection, and that it will therefore still be necessary to seek treatment for possible malaria even after vaccination. It will be stressed that some of the children enrolled in the study will receive a rabies vaccine and not the malaria vaccine, but that at the end of the trial all volunteers in groups 2 and 3 will be offered the standard rabies immunization (not as part of the trial protocol). It will be made clear that carers will not know which vaccine their child has received until the end of the study. Potential participants and their carers will be informed that we will request HIV testing for volunteers. We will explain that we would plan eventually to offer immunisation to those testing HIV positive, but that normal practice is to begin with those who are HIV negative. In order to preserve the confidentiality of those volunteers infected with HIV who are undergoing immunisation, we will not discuss the option of immunisation for those testing positive publicly, since it may lead to speculation regarding the identity of those infected with HIV, particularly if there are a variety of different regimens being used. It will also be made clear to the carer of prospective volunteers that a photograph of the child and carer will be taken to aid identification of the study participant.

After this meeting the field worker(s) will identify potential volunteers and invite them to the Sukuta Health Centre for further discussion. During these discussions, it will be stressed that this is the beginning of a long process of vaccine development, which will be accelerated by the conduct of such small trials in Africa.

At Sukuta Health Centre field workers will explain the study further to each volunteer/their carer on an individual basis. Individuals who feel that the trial is appropriate for them or their child will be invited to attend a formal screening visit. This will include a further discussion of the study with the principal investigator or study physician, and another opportunity for private discussion.

Screening visit

We will provide detailed information about the study for distribution to the volunteers/carers. The principal investigator will endeavour to ensure that all volunteers/carers fully understand the risks. Any volunteer/carer who appears to have less than complete understanding will not be enrolled. As with any experimental vaccine the volunteers/carers must understand that the vaccines have not yet been shown to prevent infection and this will be stressed during the recruitment stage. They must also understand the very small chance of anaphylactic reactions and thereby the importance of complying with the one-hour observation period after each vaccination. The information sheet covers these points in detail, and each volunteer will have the contents of the sheet explained in individual meetings on 2 separate occasions, including a quiz/ comprehension test administered prior to signing/thumb printing the form. This process is similar to that used previously and we believe has produced a high standard of informed consent.

At the screening visit following written informed consent investigators will take a clinical history, examine all volunteers carefully and conduct a number of standard laboratory tests (FBC, malaria rapid antigen test, ALT and creatinine, and dipstick urinalysisfor protein and blood to screen subjects for clinically significant acute or chronic diseases. Those with abnormal results at screening will be offered appropriate investigations and treatment or referral as necessary. Malaria antigen test positive children will be excluded from the study. An identification photograph will also be taken, as above. This will be identified by the unique study number and will be destroyed at the end of the study.

When the results of these screening investigations are available a vaccination visit will be arranged, not more than 90 days following the screening visit.

HIV testing

Volunteers/carers will have access to a trained counsellor. Volunteers will have the option to enrol without being informed of their status, although our experience previously was that all volunteers wished to know their status.

HIV serostatus (positive and negative) will be established using the standard diagnostic methods in place at the MRC Fajara. Results will be disclosed after the initial test, with an explanation that confirmatory testing is needed for those who are HIV seropositive.

We would offer follow up counselling to support further those diagnosed HIV positive. Those diagnosed positive would receive standard medical care at a local Government facility, according to Gambia Government guidelines.

9.2. Enrolment

Volunteers are considered enrolled into the study when they receive their first vaccination. This will occur not more than 90 days following the screening visit.

9.3. Follow-up

Volunteers are followed until 300 days (approximately 10 months) after the first vaccination.

Field workers will assess and record local adverse reactions including pain, swelling, discolouration and limitation of arm movement. He or she will then assess and record systemic adverse events as outlined in section 11.2. Each solicited side effect will be classified as absent, mild, moderate or severe. Due to the high anticipated background frequency of illness in children in this area, solicited and unsolicited adverse events will only be recorded up to 30 days after each vaccination for volunteers in group 2 and 3. Serious adverse events will be collected throughout the study period. Each volunteer will be visited at home daily for three days by a field worker after each vaccination and if necessary the volunteer will continue to be seen regularly until the symptom(s) have resolved. In addition the volunteers will be seen on day 14, day 63, day 90 and day 300 after vaccination for full safety and reactogenicity assessment by the Principal Investigator. Volunteers will be asked to present to the clinic if they develop any illness up to 56 days after vaccination. The emerging safety data will be described to the volunteers when they attend for the next immunisation.

Rabies immunisation will be arranged by the PI for all participants in groups 2 and 3, although this will not be covered by the trial protocol.

9.4. Study Termination

Every reasonable effort should be made to maintain protocol compliance and participation in the study. If a subject is withdrawn from the study for any reason, the reason will be recorded. If withdrawal is the result of a serious AE, the investigator will offer to arrange for appropriate specialist management of the problem and the Ethical committee will be informed in a timely manner. The extent of follow up will be determined by the investigator but will be at least for the whole study period. Subjects withdrawn prematurely for any reason will not be re-entered in to the trial, although they may be requested to return to the clinic for safety evaluation. A complete safety evaluation will be made for any subject who terminates from the study prematurely. Group 2 and 3 volunteers will also be offered the standard rabies immunisation at the end of the trial .

The following will result in study termination and will not be considered normal protocol completion:

- *Development of an AE - applies to a subject who is withdrawn from the study primarily due to a severe or serious adverse event.*
- *Lost to follow-up - applies to a subject who consistently fails to return for protocol study visits, is not reachable by telephone or other means of communication and/or is not able to be located.*
- *Research terminated by investigator - - the entire study is terminated by the investigator for any reason.*
- *Withdrawal of Consent - applies to a subject who withdraws consent to participate in the study for any reason.*
- *Protocol deviation- applies to a subject who fails to achieve critical endpoints or did not meet entrance criteria but was enrolled into the study.*
- *Other - is a category used when previous categories do not apply and requires an explanation.*

10. STUDY PROCEDURES

10.1. Clinical Evaluations

Screening Visit:

Medical history and physical examination will be performed at screening to exclude any significant medical conditions as described in Section 5.2. HLA typing will also be performed on blood taken at this visit, as outlined in Section 10.3.

D0 (Vaccination with AdCh63 ME-TRAP)

Medical history, temperature monitoring +/- physical examination will be performed and eligibility will be reviewed by the Investigator prior to vaccination. Vaccine will be administered as outlined in Section 8.4. Venepuncture for exploratory immunology and *Plasmodium falciparum* polymerase chain reaction will be performed as outlined in Section 10.3. As outlined in Sections 4.3.2 and 10.3.2, *P. falciparum* PCR testing will be performed at a later stage, the results will be used in the interpretation of immunogenicity data, and this assay is not validated for clinical diagnostic purposes. As such, PCR results will have no impact on the conduct of the study.

D1, 2 and 3

Each volunteer will be visited at home daily for three days by a field worker for assessment and recording of any solicited and unsolicited AEs in the CRF. If necessary the volunteer will continue to be seen regularly until the symptom(s) have resolved or stabilised.

D14 (+/-7 days)

Medical history, temperature monitoring +/- physical examination will be performed and recorded in the CRF. Any solicited and unsolicited AEs will be recorded. Venepuncture will be performed for FBC, ALT, Creatinine and exploratory immunology as outlined in Section 10.3.

D56 (Vaccination with MVA ME-TRAP) (- 7/ +30 days)

Medical history, temperature monitoring +/- physical examination will be performed and eligibility will be reviewed by the Investigator prior to vaccination. Vaccine will be administered as outlined in Section 8.4. Venepuncture will be performed for FBC, ALT, Creatinine and exploratory immunology as outlined in Section 10.3.

D57, 58 and 59
Each volunteer will be visited at home daily for three days by a field worker for assessment and recording of any solicited and unsolicited AEs in the CRF. If necessary the volunteer will continue to be seen regularly until the symptom(s) have resolved or stabilised.

D63 (-1/+7 days)
Medical history, temperature monitoring +/- physical examination will be performed and recorded in the CRF. Any solicited and unsolicited AEs will be recorded. Venepuncture will be performed for FBC, ALT, Creatinine and exploratory immunology as outlined in Section 10.3.

D90 (+/- 14 days)
Medical history, temperature monitoring +/- physical examination will be performed and recorded in the CRF. Any solicited and unsolicited AEs will be recorded. Venepuncture will be performed for FBC, ALT, Creatinine and exploratory immunology as outlined in Section 10.3.

D300 (+/- 28 days)
Medical history, temperature monitoring +/- physical examination will be performed and recorded in the CRF. Any solicited and unsolicited AEs will be recorded. Venepuncture will be performed for FBC, ALT, Creatinine and exploratory immunology as outlined in Section 10.3.

Medical history and physical examination directed towards specific AEs may be performed at various other time-points throughout the study, at the discretion of investigators.

10.2. Concomitant Therapy

The following data are collected for concomitant medications:

Name
Dose and frequency
Start and stop dates
Indication

10.3. Laboratory Evaluations

10.3.1. Clinical Laboratory Evaluations

FBC, ALT, Creatinine – Screening, D14, D56, D63, D90, D300.
HIV ELISA (Murex) +/- Hexagon and Peptilav (for positives only) +/- PCR for indeterminate results – Screening.
HLA testing – D0
Screening dipstick Urinalysis blood and protein

10.3.2. Special Laboratory Evaluations

Ex vivo IFN-γ ELISPOT +/- ICS +/- *Cultured* IFN-γ ELISPOT – D0, D14, D 56, D63, D90, D300
TRAP ELISA – at the discretion of investigators
Anti-AdCh63 neutralising antibody – D0 and additional time-points at the discretion of investigators

Plasma and cells will be stored at -20°C and -192°C respectively. *Ex vivo* ELISPOTs are currently being performed in the laboratory at the MRC, Fajara and results are validated by rigorous use of negative and positive controls. This technique uses an overnight stimulation by antigen of separated lymphocytes from the volunteer's blood sample to count the number of interferon gamma producing cells. Analysis by FACS,

where sufficient numbers of lymphocytes are isolated, will allow further characterisation of the response, by co-staining lymphocytes with CD4 and CD8 as well as IFN gamma. This may be performed on fresh or frozen cells.

Other immunology studies, including gene expression studies as an exploratory analysis, may be performed at the time-points in the schedule of attendances at the discretion of investigators.

A sample of blood from venepuncture on Day 0 will be stored for later testing by polymerase chain reaction (PCR) for *Plasmodium falciparum*.

11. ASSESSMENTS AND EVALUATIONS

11.1. Definition of criteria

11.1.1. Primary Evaluation Criteria

Assessment of safety and tolerability.

11.1.2. Secondary & Tertiary Evaluation Criteria

Assessment of immunogenicity.

11.2. Parameters to be recorded

Safety:

The following solicited AEs (Table 1) will be recorded in the AE section of the CRF.

**Adverse events**	
**Local (injection site)**	Pain at the injection site
	Redness/Discolouration at the injection site
	Swelling at injection site
	Warmth at the injection site
	Itch at the injection site (not for group 2/3)
	Scaling/Pustules/Blistering at injection site
	Limitation of arm movement (group 2/3)
**General – Adults**	Documented fever (Axillary temperature > 37.5° C)
	Symptoms of feverishness
	Malaise
	Arthralgia
	Headache
	Myalgia
	Nausea / vomiting
	Other (specify)
**General – Children aged 2-6**	Documented fever (Axillary temperature > 38° C)
	Reported fever by carer
	Reduced oral intake
	Reduced activity
	Vomiting

Table 1: Solicited local and general adverse events documented in the CRF

11.3. Method and Timing of measurements

11.3.1. Definitions

Definitions for the terms adverse event (or experience), adverse reaction, and unexpected adverse reaction have previously been agreed to by consensus of the more than 30 Collaborating Centres of the WHO International Drug Monitoring Centre (Uppsala, Sweden). Although those definitions can pertain to situations involving clinical investigations, some minor modifications are necessary, especially to accommodate the pre-approval, development environment.

The following definitions, with input from the WHO Collaborative Centre, have been agreed:

11.3.2. Adverse Event

Any untoward medical occurrence in a clinical investigation subject occurring in any phase of the clinical study whether or not considered related to the vaccine. This includes an exacerbation of pre-existing conditions or events, intercurrent illnesses, or vaccine or drug interaction. Anticipated day-to-day fluctuations of pre-existing conditions, including the disease under study, that do not represent a clinically significant exacerbation will not be considered adverse events. Discrete episodes of chronic conditions occurring during a study period will be reported as adverse events in order to assess changes in frequency or severity.

Adverse events will be documented in terms of a medical diagnosis(es). When this is not possible, the adverse event will be documented in terms of signs and symptoms observed by the investigator or reported by the subject at each study visit.

Pre-existing conditions or signs and/or symptoms (including any which are not recognised at study entry but are recognised during the study period) present in a subject prior to the start of the study will be recorded on the Medical History form within the subject's CRF.

11.3.3. Adverse Reactions

In the pre-approval clinical experience with a new medicinal product or its new usages, particularly as the therapeutic dose(s) may not be established, all noxious and unintended responses to a medicinal product related to any dose should be considered adverse reactions.
The phrase “responses to a medicinal product” means that a causal relationship between a medicinal product and an adverse event is at least a reasonable possibility, *i.e.* the relationship cannot be ruled out.

11.3.4. Unexpected Adverse Reactions

An adverse reaction, the nature or severity of which is not consistent with the applicable product information (*e.g.* Investigator's Brochure for an unapproved investigational medicinal product) is considered as an unexpected adverse reaction.

11.3.5. Serious Adverse Events

A serious adverse event (experience) or reaction is any untoward medical occurrence that at any dose:

- results in death,
- is life-threatening,

Note: The term "life-threatening" in the definition of "serious" refers to an event in which the patient was at risk of death at the time of the event; it does not refer to an event which hypothetically might have caused death if it were more severe.

- requires inpatient hospitalisation or prolongation of existing hospitalisation,
- results in persistent or significant disability/incapacity, or
- is a congenital anomaly/birth defect.

Medical and scientific judgment should be exercised in deciding whether expedited reporting is appropriate in other situations, such as important medical events that may not be immediately life-threatening or result in death or hospitalisation but may jeopardise the patient or may require intervention to prevent one of the other outcomes listed in the definition above. These should also usually be considered serious.

11.3.6. Collection of Adverse Events

At each visit vital signs will be documented on the CRF together with local reactions at the injection site. The largest diameter through the injection site of any redness will be recorded in millimetres. The largest diameter through the injection site of local swelling, defined as a more generalized swelling of the deltoid muscle will be recorded in millimetres. Severity of these local findings will be graded using the scale given in Table 2.

Table 2: Grading a) for swelling

Grade	**Diameter [mm]**
0	0
1	< 20
2	20 – 50
3	> 50

b) for redness

Grade	**Diameter [mm]**
0	0
1	< 50
2	50 – 100
3	> 100

Study subjects/carers will be asked to indicate the maximum degree of pain they experience at the injection site using a scale ranging from 0 to 3 as described in Table 3.

Table 3: Pain scale (adults and children if able to report) and limitation of arm movement scale

**Grade**	**Description**
**0**	No pain at all
**1**	Painful on touch, no restriction in movement of arms, able to work, drive, carry heavy objects as normal
**2**	Painful when limb is moved<br>(*i.e.* restriction in range of movement in arm, difficulty in carrying objects)
**3**	Severe pain at rest<br>(*i.e.* unable to use arm due to pain.)

Limitation of arm movement

**Grade**	**Description**
**0**	No limitation (> 180 degrees)
**1**	Limitation to < 180 degrees
**2**	Limitation to < 90 degrees
**3**	Limitation to < 30 degrees

All local reactions will be considered causally related to the vaccination.

At each visit subjects will be requested to report local and general side effects they might have experienced since they last were seen. These will only be recorded in the CRF if they occurred up to 30 days post vaccination, unless they meet the criteria for serious adverse event as outlined below. The investigator will assess the severity of the solicited signs and symptoms using the key provided in Tables 4 and 5.

Table 4: Intensity of the general adverse events in adults will be assessed as described:

GRADE 0	None
GRADE 1	Mild: Transient or mild discomfort (< 48 hours); no medical intervention/therapy required
GRADE 2	Moderate: Mild to moderate limitation in activity - some assistance may be needed; no or minimal medical intervention/therapy required
GRADE 3	Severe: Marked limitation in activity, some assistance usually required; medical intervention/therapy required.

Table 5: Intensity of the general adverse events in children (aged 2-6) will be assessed as described:

Systemic (General)	Grade 1	Grade 2	Grade 3
Fever (axillary temp)	≥38C- ≤39C	≥39C - ≤40C	≥40C
Decreased oral intake	Minimal decrease in oral intake	Below 50% of normal oral intake in 24 hr	No oral intake in 24hr
Vomiting	1 episode in 24hr; no interference with activity	2-3 episodes in 24 hr OR some interference with activity	> 3 episodes in 24 hours OR prevents daily activity
Diarrhea	Unformed stool OR 1-3 more stools than baseline in 24 hr	Partially liquid stools OR 4-6 more stools than baseline in 24hr	Completely liquid stools OR >6 more stools than baseline in 24 hr
Reduced activity	Minimal interference with activity	Below 50% of normal activity in 24 hr	Prevents daily activity
Unsolicited adverse events	Minimal interference with activity	Below 50% of normal activity in 24 hr	Prevents daily activity

Activity for the purposes of this assessment will be considered oral intake, sleep and play.

The investigator using the guidelines provided in section 11.3.8. will assess the relationship of the event to the administration of the vaccine. Both severity of the event and its relationship to the vaccine administration will be documented in the CRF if occurring up to 30 days post vaccination for groups 2 and 3, unless it fulfils criteria for a serious adverse event (section 11.3.5).

Further details for any AE (such as start/stop date and any treatment) will also be gathered, regardless of the relationship to the vaccine in sufficient detail to also allow assessment of the AE according to case definitions published by the Brighton Collaboration [62]. Space is allocated on the CRF to document any unsolicited adverse event reported by the volunteer/carer up to day 30 post-vaccination for groups 2 & 3, and throughout the study period for group 1. Serious adverse events (SAE) as defined in section 11.3.5 of the protocol will be documented in the CRF and reported as described.

Adverse events will not be collected on volunteers in groups 2 and 3 given rabies vaccine at the end of the trial.

11.3.7. Follow-up of Adverse Events

Adverse events possibly, probably or definitely related to the vaccine, whether serious or not, which persist at the end of the trial will be followed up by the investigator until their complete resolution or stabilisation. Moreover, any serious adverse event possibly, probably or definitely related to the vaccine and occurring after trial termination should be reported by the investigator according to the procedure described below.

Outcome of any non-serious adverse event occurring within 30 days post-vaccination or any SAE reported during the entire study will be assessed as:

- *Recovered/resolved*
- *Not recovered/not resolved*
- *Recovering/resolving*
- *Recovered with sequelae/resolved with sequelae*
- *Fatal (SAEs only)*

Subjects who have moderate or severe on-going adverse events at the completion of the study will be advised to consult a physician if the event is not considered to be related to the study vaccine. A follow-up visit will be arranged to manage the problem and to determine the severity and duration of the event, if it is considered to be related to the study vaccine. If appropriate, specialist review will be arranged by MRC investigators.

11.3.8. Reporting of Adverse Events

Every SAE occurring throughout the trial must be reported by telephone, email or fax to the sponsor, LSM and DSMB by the investigator as soon as (s)he is alerted of it and within one working day, even if the investigator considers that the adverse event is not related to vaccination. The investigator will then complete a SAE report form as soon as possible and within five working days or seven calendar days.

Any relevant information concerning the adverse event that becomes available after the SAE report form has been sent (outcome, precise description of medical history, results of the investigation, copy of hospitalisation report, etc.) will be forwarded to the Sponsor in a timely manner. The anonymity of the subjects shall be respected when forwarding this information.

SAEs that are suspected to be related to the vaccine will be reported to the Ethics Committee within 15 calendar days of the site becoming aware of the event. If the event is fatal or life-threatening, the event will be reported within 7 calendar days.

Suspected unexpected serious adverse reactions (SUSARs) will be reported according to national regulatory guidelines. The sponsor pledges to inform the Authorities of any trial discontinuation and specify the reason for discontinuation.

The causal relationship between the AE and the product will first be evaluated by the investigator with the following scale:

No relationship:
No temporal relationship to study product; *and*
Alternate aetiology (clinical state, environmental or other interventions); *and*
Does not follow known pattern of response to study product

Possible relationship:
Reasonable temporal relationship to study product; *or*
Event not readily produced by clinical state, environmental or other interventions; *or*
Similar pattern of response to that seen with other vaccines

Probable relationship:
Reasonable temporal relationship to study product; *and*
Event not readily produced by clinical state, environment, or other interventions *or*

Known pattern of response seen with other vaccines

Definite relationship:
Reasonable temporal relationship to study product; *and*
Event not readily produced by clinical state, environment, or other interventions; *and*
Known pattern of response seen with other vaccines

11.3.9. Pregnancy

Not applicable.

12. STATISTICS

12.1. Study Design and Hypothesis

This is an single blinded controlled phase I dose-escalation safety study. As this is a descriptive study to acquire safety data in a small group of volunteers, null hypotheses are not relevant.
The study design will allow a comparison between safety and tolerability of two doses of AdCh63 ME-TRAP.
The secondary objective is to obtain immunogenicity data on the use of these vaccines. This may be used to compare the immunogenicity of two doses of AdCh63 ME-TRAP.
It will also allow a comparison between the safety and tolerability of two doses of MVA ME-TRAP in children, although this is a tertiary endpoint.

12.2. Study Population

We will enrol 16 healthy male volunteers between the ages of 18 and 50 years, 6 in group 1A and 10 in group 1B. We will enrol 6 healthy children aged between 2 and 6 years in each of the following groups; 2A, 2B, 2C, 3A, 3B and 3C. It is anticipated that most volunteers will be recruited from the region served by Sukuta Health Centre.

12.3. Sample Size Considerations

The sample size for phase I studies balances the need to avoid exposing a large group to an unknown risk with the need for data on an adequate sample. The aim is not to provide pre-marketing safety data, but to justify the future study of larger groups.

12.4. Planned Interim Analysis

12.4.1. Safety Review

Group 1:
The frequency, intensity and relationship to vaccination of solicited and unsolicited adverse events with a dose of 1 x 10^{10}vp AdCh63 ME-TRAP in adults (Group 1A) will be reviewed by the LSM prior to dose escalation to 5 x 10^{10}vp (Group 1B).

Groups 2 & 3:

The frequency, intensity and relationship to vaccination of solicited and unsolicited adverse events will be reviewed as outlined in section 4.4.1.

12.4.2. Immunogenicity or Efficacy Review

No interim immunogenicity analysis is planned.

12.5. Final Analysis Plan

No formal statistical analysis is planned for this study.

13. DATA HANDLING AND RECORD KEEPING

13.1. Data Management

The Principal Investigator will be responsible for receiving, entering, cleaning, querying, analysing and storing all data that accrues from the study. Responsibility for this may be delegated to the data manager at MRC. The data will be entered into the subjects' CRFs. Data will be subsequently transferred to an electronic database for analysis.

If any changes to the protocol are necessary during the study a formal amendment will be presented to the sponsor prior to submission to the relevant ethical and regulatory agencies for approval unless to eliminate an immediate hazard(s) to study participant without prior ethics approval. Any unforeseen and unavoidable deviations from the protocol will be documented and filed in as a protocol deviation in the Trial Master File, with explanation.

13.2. Data Capture Methods

Data capture will be on paper CRFs. The CRFs will be considered source documents as healthy volunteers will not have hospital case-notes.
Adverse events will be tabulated in an electronic database (OpenClinica®) for descriptive analysis.
Immunological data will be transferred to an electronic database for analysis without any volunteer identifier apart from the unique volunteer number.

13.3. Types of Data

Data collected will include solicited and non-solicited adverse event data, concomitant medications, clinical laboratory and exploratory immunology data. Source documents will include laboratory results and the case record file containing the case report forms for each volunteer as the healthy volunteers participating in this study may not have medical notes.

13.4. Timing/Reports

Annual Safety Report: Due on anniversary of Regulatory Approval – sent to Regulatory and Ethical Bodies
Annual Progress Report: Due on anniversary of Ethical Approval – sent to Ethics Committee

13.5. Archiving

The investigator must keep all trial documents for at least 15 years after the completion or discontinuation of the trial, whatever the nature of the investigational centre (private practice, hospital, institution).

13.6. Protocol Deviations

Any unforeseen and unavoidable deviations from the protocol will be documented and filed in a protocol deviation folder, with explanation.

14. Data Access and Quality Assurance

14.1. Direct Access to Source Data/Documents

The principal investigator will provide direct access to the source data documents to the Ethics Committee, to the regulatory agency, and to authorised representatives of the sponsor, permitting trial-related monitoring and audits.

14.2. Quality Assurance

14.2.1. Modifications to the Protocol

Any amendments to the trial that appear necessary during the course of the trial must be discussed by the investigator and sponsor concurrently unless to eliminate an immediate hazard(s) to study participants. If agreement is reached concerning the need for an amendment, it will be produced in writing by the sponsor and/or the investigator and will be made a formal part of the protocol. An amendment requires Ethics Committee approval.

All amendments must also be transmitted to Regulatory Authorities, if applicable.

An administrative change to the protocol is one that modifies administrative and logistical aspects of a protocol but does not affect the subjects' safety, the objectives of the trial and its progress. An administrative change does not require Ethics Committee approval. However, the Ethics committee must be notified whenever an administrative change is made.

The investigator is responsible for insuring that changes to an approved trial, during the period for which Ethics Committee approval has already been given, are not initiated without Ethics Committee review and approval except to eliminate apparent immediate hazards to the subject.

14.3. Monitoring

14.3.1. Initiation Visit

An initiation visit will be performed before the inclusion of the first subject in the study. The Monitor will verify and document that the material to be used during the trial has been received and that the investigational team has been properly informed about the trial and regulatory requirements.

14.3.2. Follow-Up Visits

The Monitor will carry out regular follow-up visits. The investigator commits to being available for these visits and to allow the monitoring staff direct access to subject medical files, if existing, and CRFs. The Monitor is committed to professional secrecy.

During the visits, the Monitor may:

- Carry out a quality control of trial progress: in respect of protocol and operating guidelines, data collection, signature of consent forms, completion of documents, SAE, sample and product management, cold chain monitoring
- Inspect the CRFs, TMF and correspondent correction sheets

The Monitor will discuss any problem with the investigator and define with him the actions to be taken.

14.3.3. Close-out Visit

A close-out visit will be performed at the end of the trial. Its goals are to make sure that:

- The centre has all the documents necessary for archiving
- All unused material has been recovered
- All vaccines have been accounted for

15. FINANCE

Justification of the Budget

The resources requested are for the proper conduct of the phase I trials and related activities in The Gambia. All salaries are based on MRC Laboratories, Gambia pay scales. A study physician is needed to conduct the trial under the supervision of the PI and Dr Katie Flanagan. 3 nurses/field workers are needed to help with the recruitment, follow-up and provide nursing care for the study subjects. Three laboratory assistants are needed to provide laboratory support for the study. A full time driver is needed to help with transportation of staff and study subjects for the duration of the study. A project manager is needed to ensure that all procedures and their documentation meet GCP and GCLP quality standards.

16. ETHICAL CONSIDERATIONS

16.1. Good Clinical Practice

This trial will be conducted in accordance with the Declaration of Helsinki as agreed by the World Medical Association General Assembly (Seoul 2008), ICH Good Clinical Practice and local regulatory requirements.

16.2. Ethical Review

Before the inclusion of the first subject in the study, the protocol must be approved by Ethical Review Committees in The Gambia and Oxford (OXTREC).

16.3. Informed Consent

The volunteer should give written informed consent before being included in the trial, after having been informed of the nature of the trial, the potential risks and their obligations. Informed consent forms will be provided in duplicate (original kept by the investigator, one copy kept by the subject or the subject's legally acceptable representative).

Please refer to Section 9.1 for a detailed discussion of the informed consent process.

16.4. Special Considerations

Potential subjects with HIV

Although it is standard practice to obtain safety data in healthy subjects before immunising those with HIV, we do not believe the vaccine to be unsafe in HIV. The need for an HIV test before immunisation would preclude widespread delivery of a successful immunisation, and it is clearly desirable to protect HIV infected individuals as well as uninfected. These highly attenuated vectors are unable to replicate in human cells, and are very likely to be safe in HIV. MVA vectored vaccines have been used safely in volunteers with HIV infection in small studies, and adults infected with HIV have previously received MVA ME-TRAP. In the current protocol HIV infection will be an exclusion criterion.

Confidentiality
All HIV tests will be identified by a unique code number only. The code key will be kept by the principal investigator who will be responsible for holding these files securely.

All other blood results and adverse event data will be encoded in an electronic database and stored securely by the principal investigator.

Maintaining confidentiality
A potential danger of excluding volunteers who are HIV positive is that excluded volunteers will be presumed to be HIV positive. We therefore propose to over recruit in this study and have stipulated other exclusion criteria. It would therefore not be a foregone conclusion that any excluded volunteer would be HIV positive, and this will be explicit in community and individual discussions. This approach has been used successfully previously, and we believe has protected the confidentiality of those screening positive. Only the principal investigator and a designee, if applicable, will be able to link HIV test results for the study population to individuals.

Inducement
There may be a perception amongst volunteers of benefit from physical examination, laboratory screening and HIV testing in the current study, in addition to free health care provided during the study period for non-vaccine related medical problems. We will also offer compensation for transport expenses for all study subjects, and time away from work or duties at home for adults study subjects in order to attend for immunisation and follow-up.
We do not feel these benefits are excessive, and believe it would be unreasonable to request the cooperation of a population in regular employment or with childcare responsibilities without offering compensation for time.

The use of a licensed vaccine in this trial may be considered to provide advantage to the control group over the vaccine group; therefore all volunteers in groups 2 and 3 will be offered a standard course of rabies vaccine at the end of the trial. This will be arranged by Dr K. Bojang but will not be considered part of this trial.

17. INDEMNITY/COMPENSATION/INSURANCE

17.1. Indemnity

Compensation for any injury caused by taking part in this study will be in accordance with the guidelines of the Association of the British Pharmaceutical Industry (ABPI). Broadly speaking the ABPI guidelines recommend that 'the sponsor', without legal commitment, should compensate participants without them having to prove that it is at fault. This applies in cases where it is likely that such injury results from giving any new drug or any other procedure carried out in accordance with the protocol for the study. 'The

sponsor' will not compensate participants where such injury results from any procedure carried out which is not in accordance with the protocol for the study. Participants' right at law to claim compensation for injury where negligence can be proven is not affected. In this instance the University of Oxford is the Research Sponsor Institution.

17.2. Compensation

- Compensation for adult male volunteers will be calculated on the basis of time lost to employment as a result of participation in the study.
  Each visit with blood test will be compensated by the equivalent of one full day's wage. Each vaccination visit will be compensated by the equivalent of two days wages.
  Carers of children enrolled in the study will be offered compensation for transport expenses.

17.3. Insurance

Oxford University Investigators participating in this trial will receive insurance coverage from the University clinical trials insurance policy.

18. REFERENCES

1. Okiro EA, Hay SI, Gikandi PW, et al. The decline in paediatric malaria admissions on the coast of Kenya. Malar J 2007;6:151
2. Sievers AC, Lewey J, Musafiri P, et al. Reduced paediatric hospitalizations for malaria and febrile illness patterns following implementation of community-based malaria control programme in rural Rwanda. Malar J 2008;7:167
3. O'Meara WP, Mwangi TW, Williams TN, McKenzie FE, Snow RW and Marsh K. Relationship between exposure, clinical malaria, and age in an area of changing transmission intensity. Am J Trop Med Hyg 2008;79:185-91
4. Ceesay SJ, Casals-Pascual C, Erskine J, et al. Changes in malaria indices between 1999 and 2007 in The Gambia: a retrospective analysis. Lancet 2008;372:1545-54
5. O'Meara WP, Bejon P, Mwangi TW, et al. Effect of a fall in malaria transmission on morbidity and mortality in Kilifi, Kenya. Lancet 2008;372:1555-62
6. Greenwood BM, Fidock DA, Kyle DE, et al. Malaria: progress, perils, and prospects for eradication. J Clin Invest 2008;118:1266-76
7. Snow RW, Guerra CA, Noor AM, Myint HY and Hay SI. The global distribution of clinical episodes of Plasmodium falciparum malaria. Nature 2005;434:214-7
8. Snow RW, Craig M, Deichmann U and Marsh K. Estimating mortality, morbidity and disability due to malaria among Africa's non-pregnant population. Bull World Health Organ 1999;77:624-640
9. Stapleton DH. Lessons of history? Anti-malaria strategies of the International Health Board and the Rockefeller Foundation from the 1920s to the era of DDT. Public Health Rep 2004;119:206-15
10. Carter ED. "God bless General Peron": DDT and the endgame of malaria eradication in Argentina in the 1940s. J Hist Med Allied Sci 2009;64:78-122
11. Bruce-Chwatt LJ. Malaria and its control: present situation and future prospects. Annu Rev Public Health 1987;8:75-110
12. Feachem RG, Phillips AA, Targett G and Group TME. Shrinking the malaria map. San Fransisco: The Global Health Group, 2009
13. Penny MA, Maire N, Studer A, Schapira A and Smith TA. What should vaccine developers ask? Simulation of the effectiveness of malaria vaccines. PLoS ONE 2008;3:e3193

14. Bojang KA, Milligan PJ, Pinder M, et al. Efficacy of RTS,S/AS02 malaria vaccine against Plasmodium falciparum infection in semi-immune adult men in The Gambia: a randomised trial. Lancet 2001;358:1927-34
15. Aponte JJ, Aide P, Renom M, et al. Safety of the RTS,S/AS02D candidate malaria vaccine in infants living in a highly endemic area of Mozambique: a double blind randomised controlled phase I/IIb trial. Lancet 2007;370:1543-51
16. Alonso PL, Sacarlal J, Aponte JJ, et al. Efficacy of the RTS,S/AS02A vaccine against Plasmodium falciparum infection and disease in young African children: randomised controlled trial. Lancet 2004;364:1411-20
17. Bejon P, Lusingu J, Olotu A, et al. Efficacy of RTS,S/AS01E vaccine against malaria in children 5 to 17 months of age. N Engl J Med 2008;359:2521-32
18. Nussenzweig RS, Vanderberg J, Most H and Orton C. Protective immunity produced by the injection of x-irradiated sporozoites of plasmodium berghei. Nature 1967;216:160-2
19. Hill AV, Allsopp CE, Kwiatkowski D, et al. Common west African HLA antigens are associated with protection from severe malaria. Nature 1991;352:595-600
20. Hill AV, Elvin J, Willis AC, et al. Molecular analysis of the association of HLA-B53 and resistance to severe malaria. Nature 1992;360:434-9
21. Todryk SM, Bejon P, Mwangi T, et al. Correlation of memory T cell responses against TRAP with protection from clinical malaria, and CD4 CD25 high T cells with susceptibility in Kenyans. PLoS One 2008;3:e2027
22. Khusmith S, Sedegah M and Hoffman SL. Complete protection against Plasmodium yoelii by adoptive transfer of a CD8+ cytotoxic T-cell clone recognizing sporozoite surface protein 2. Infect Immun 1994;62:2979-83
23. Romero P, Maryanski JL, Corradin G, Nussenzweig RS, Nussenzweig V and Zavala F. Cloned cytotoxic T cells recognize an epitope in the circumsporozoite protein and protect against malaria. Nature 1989;341:323-6
24. Roestenberg M, McCall M, Hopman J, et al. Protection against a malaria challenge by sporozoite inoculation. N Engl J Med 2009;361:468-77
25. Schofield L, Villaquiran J, Ferreira A, Schellekens H, Nussenzweig R and Nussenzweig V. Gamma interferon, CD8+ T cells and antibodies required for immunity to malaria sporozoites. Nature 1987;330:664-6
26. Hoffman SL, Isenbarger D, Long GW, et al. Sporozoite vaccine induces genetically restricted T cell elimination of malaria from hepatocytes. Science 1989;244:1078-81
27. Weiss WR, Mellouk S, Houghten RA, et al. Cytotoxic T cells recognize a peptide from the circumsporozoite protein on malaria-infected hepatocytes. J Exp Med 1990;171:763-73
28. Bejon P, Mwacharo J, Kai O, et al. The induction and persistence of T cell IFN-gamma responses after vaccination or natural exposure is suppressed by Plasmodium falciparum. J Immunol 2007;179:4193-201
29. Webster DP, Dunachie S, Vuola JM, et al. Enhanced T cell-mediated protection against malaria in human challenges by using the recombinant poxviruses FP9 and modified vaccinia virus Ankara. Proc Natl Acad Sci U S A 2005;102:4836-41
30. Bejon P, Peshu N, Gilbert SC, et al. Safety profile of the viral vectors of attenuated fowlpox strain FP9 and modified vaccinia virus Ankara recombinant for either of 2 preerythrocytic malaria antigens, ME-TRAP or the circumsporozoite protein, in children and adults in Kenya. Clin Infect Dis 2006;42:1102-10
31. Bejon P, Mwacharo J, Kai OK, et al. Immunogenicity of the candidate malaria vaccines FP9 and modified vaccinia virus Ankara encoding the pre-erythrocytic antigen ME-TRAP in 1-6 year old children in a malaria endemic area. Vaccine 2006
32. Bejon P, Mwacharo J, Kai O, et al. A Phase 2b Randomised Trial of the Candidate Malaria Vaccines FP9 ME-TRAP and MVA ME-TRAP among Children in Kenya. PLoS Clin Trials 2006;1:e29
33. Bejon P, Kai OK, Mwacharo J, et al. Alternating vector immunizations encoding pre-erythrocytic malaria antigens enhance memory responses in a malaria endemic area. Eur J Immunol 2006;36:2264-72
34. Reyes-Sandoval A, Sridhar S, Berthoud T, et al. Single-dose immunogenicity and protective efficacy of simian adenoviral vectors against Plasmodium berghei. Eur J Immunol 2008;38:732-41

35. Sridhar S, Reyes-Sandoval A, Draper SJ, et al. Single-dose protection against Plasmodium berghei by a simian adenovirus vector using a human cytomegalovirus promoter containing intron A. J Virol 2008;82:3822-33
36. Schneider J, Langermans JA, Gilbert SC, et al. A prime-boost immunisation regimen using DNA followed by recombinant modified vaccinia virus Ankara induces strong cellular immune responses against the Plasmodium falciparum TRAP antigen in chimpanzees. Vaccine 2001;19:4595-602
37. Moorthy VS, Imoukhuede EB, Milligan P, et al. A Randomised, Double-Blind, Controlled Vaccine Efficacy Trial of DNA/MVA ME-TRAP Against Malaria Infection in Gambian Adults. Plos Med 2004;1:e33
38. Bejon P, Andrews L, Andersen RF, et al. Calculation of liver-to-blood inocula, parasite growth rates, and preerythrocytic vaccine efficacy, from serial quantitative polymerase chain reaction studies of volunteers challenged with malaria sporozoites. J Infect Dis 2005;191:619-26
39. Mayr A, Stickl H, Muller HK, Danner K and Singer H. [The smallpox vaccination strain MVA: marker, genetic structure, experience gained with the parenteral vaccination and behavior in organisms with a debilitated defence mechanism (author's transl)]. Zentralbl Bakteriol [B] 1978;167:375-90
40. Mahnel H, Mayr A. [Experiences with immunization against orthopox viruses of humans and animals using vaccine strain MVA]. Berl Munch Tierarztl Wochenschr 1994;107:253-6
41. Stickl H, Hochstein-Mintzel V, Mayr A, Huber HC, Schafer H and Holzner A. [MVA vaccination against smallpox: clinical tests with an attenuated live vaccinia virus strain (MVA) (author's transl)]. Dtsch Med Wochenschr 1974;99:2386-92
42. Top FH, Jr., Grossman RA, Bartelloni PJ, et al. Immunization with live types 7 and 4 adenovirus vaccines. I. Safety, infectivity, antigenicity, and potency of adenovirus type 7 vaccine in humans. J Infect Dis 1971;124:148-54
43. Tatsis N, Ertl HC. Adenoviruses as vaccine vectors. Mol Ther 2004;10:616-29
44. Kobinger GP, Feldmann H, Zhi Y, et al. Chimpanzee adenovirus vaccine protects against Zaire Ebola virus. Virology 2006;346:394-401
45. Casimiro DR, Chen L, Fu TM, et al. Comparative immunogenicity in rhesus monkeys of DNA plasmid, recombinant vaccinia virus, and replication-defective adenovirus vectors expressing a human immunodeficiency virus type 1 gag gene. J Virol 2003;77:6305-13
46. Xiang Z, Gao G, Reyes-Sandoval A, et al. Novel, chimpanzee serotype 68-based adenoviral vaccine carrier for induction of antibodies to a transgene product. J Virol 2002;76:2667-75
47. Catanzaro AT, Koup RA, Roederer M, et al. Phase 1 safety and immunogenicity evaluation of a multiclade HIV-1 candidate vaccine delivered by a replication-defective recombinant adenovirus vector. J Infect Dis 2006;194:1638-49
48. Harro CD, Robertson MN, Lally MA, et al. Safety and immunogenicity of adenovirus-vectored near-consensus HIV type 1 clade B gag vaccines in healthy adults. AIDS Res Hum Retroviruses 2009;25:103-14
49. Tatsis N, Blejer A, Lasaro MO, et al. A CD46-binding Chimpanzee Adenovirus Vector as a Vaccine Carrier. Mol Ther 2007
50. Dudareva M, Andrews L, Gilbert SC, et al. Prevalence of serum neutralizing antibodies against chimpanzee adenovirus 63 and human adenovirus 5 in Kenyan Children, in the context of vaccine vector efficacy. Vaccine 2009;27:3501-4
51. Gilbert SC, Plebanski M, Harris SJ, et al. A protein particle vaccine containing multiple malaria epitopes. Nat Biotechnol 1997;15:1280-4
52. Robson KJ, Hall JR, Jennings MW, et al. A highly conserved amino-acid sequence in thrombospondin, properdin and in proteins from sporozoites and blood stages of a human malaria parasite. Nature 1988;335:79-82
53. Lalvani A, Aidoo M, Allsopp CE, Plebanski M, Whittle HC and Hill AV. An HLA-based approach to the design of a CTL-inducing vaccine against Plasmodium falciparum. Res Immunol 1994;145:461-8
54. Rogers WO, Malik A, Mellouk S, et al. Characterization of Plasmodium falciparum sporozoite surface protein 2. Proc Natl Acad Sci U S A 1992;89:9176-80
55. Walther M, Thompson FM, Dunachie S, et al. Safety, immunogenicity, and efficacy of prime-boost immunization with recombinant poxvirus FP9 and modified vaccinia virus Ankara encoding the full-length Plasmodium falciparum circumsporozoite protein. Infect Immun 2006;74:2706-16

56. Plebanski M, Gilbert SC, Schneider J, et al. Protection from Plasmodium berghei infection by priming and boosting T cells to a single class I-restricted epitope with recombinant carriers suitable for human use. Eur J Immunol 1998;28:4345-55
57. Reece WH, Pinder M, Gothard PK, et al. A CD4(+) T-cell immune response to a conserved epitope in the circumsporozoite protein correlates with protection from natural Plasmodium falciparum infection and disease. Nat Med 2004;10:406-10
58. Keating SM, Bejon P, Berthoud T, et al. Durable Human Memory T Cells Quantifiable by Cultured Enzyme-Linked Immunospot Assays Are Induced by Heterologous Prime Boost Immunization and Correlate with Protection against Malaria. J Immunol 2005;175:5675-80
59. Bavarian-Nordic. Bavarian Nordic's HIV Vaccine Candidate Reveals Strong Saftey and Immunogenicity Profile in HIV Infected Patients. Copenhagen: Baverian-Nordic, 2002
60. Xia Jin MR, Shady Barsoum, Geoffrey Deschenes, Lei Ba, James Binley, Daryl Schiller, Daniel bauer, David Ho, Martin Markowitz. Saftey and Immunogencitiy of ALVAC vCP1452 and recombinant gp160 in Newly Human Immunodeficiency Virus Type 1 infected patients treated with prolonged Highly Active Antiretroviral Therapy. Journal of Virology 2002;76:2206-2216
61. Gray GC, McCarthy T, Lebeck MG, et al. Genotype prevalence and risk factors for severe clinical adenovirus infection, United States 2004-2006. Clin Infect Dis 2007;45:1120-31
62. Bonhoeffer J, Bentsi-Enchill A, Chen RT, et al. Guidelines for collection, analysis and presentation of vaccine safety data in pre- and post-licensure clinical studies. Vaccine 2009;27:2282-8
