# Supplementary material for: Safety and Immunogenicity of Heterologous Prime-Boost Immunisation with Plasmodium falciparum Malaria Candidate Vaccines, ChAd63 ME-TRAP and MVA ME-TRAP, in Healthy Gambian and Kenyan Adults
Source: PLoS One. 2013 Mar 19;8(3):e57726. doi: 10.1371/journal.pone.0057726 (PMC3602521; doi:10.1371/journal.pone.0057726)
Supplement: Protocol S2 — Clinical trial protocol for Trial B. (PDF) [file pone.0057726.s006.pdf]

1) Title of project

Safety and immunogenicity of heterologous prime-boost with the candidate malaria vaccines AdCh63 ME-TRAP and MVA ME-TRAP in healthy adults in a malaria endemic area.

2) Investigators

Roma Chilengi (PI)
Patricia Njuguna
Britta Urban
Peninah Soipei
Philip Bejon
Ken Awoundo
Jimmy Shangala
Kevin Marsh
(Kenya Medical Research Institute, Centre for Geographic Medicine Research – Coast)

Christopher Duncan
Alison Lawrie
Adrian Hill
(Jenner Institute, Nuffield Dept. of Clinical Medicine, University of Oxford)

Vincent Mwatemo
(Rea Vipingo Plantation Clinic)

Data Safety and Monitoring Board:
Professor Geoff Target (Chair)

3) Abbreviations

**AdCh63 ME**	Recombinant chimpanzee adenovirus 63 encoding a sequence from a multi epitope TRAP
**AdHu**	Human Adenovirus
**ALT**	ALanine Transfarase
**CCRC**	Comprehensive Care and Research Centre
**CRF**	Case Report Form
**ICS**	Intracellular cytokine staining
**CEF**	Chicken Embryo Fibroblasts
**CGMRC**	Centre for Geographical Medicine Coast
**CTF**	Clinical Trials Facility
**CTL**	Cytotoxic T-Lymphocytes
**CS**	Circumsporozoite
**ERC**	Ethical Review Committee
**ELISPOT**	Enzyme linked immunospot
**DSMB**	Data Safety and Monitoring Board
**FBC**	Full Blood Count
**FACS**	Florecence - activated Cell Sorting
**FP9**	Fow pox 9
**HLA**	Human Leukocyte Antigen
**HIV**	human Immuno Deficiency Virus
**IFNγ**	Interferon- gamma

**IB**	Investigator Brochure
**KEMRI**	Kenya Medical Research Institute
**KDH**	Kilifi District Hospital
**MVA**	Modified Vaccinia Ankara
**PI**	Principal Investigator
**PPB**	Pharmacy and Poisons Board
**PBMC**	Peripheral Blood Mononuclear Cell
**SSC**	Scientific Steering Committee
**SFC**	Spot Forming Cells
**SAE**	Serious Adverse Event
**SOP**	Standard Operating Procedure
**TRAP**	Thrombospondin Related Adhesion Protein
**MVA ME TRAP**	Recombinant modified vaccinia virus Ankara encoding a multi epitope sequence from malaria parasites
**VCT**	Voluntary Counselling and Testing
**VP**	Viral particles
**VV**	Vaccinia virus

4) Abstract

Malaria transmission is falling in some parts of Africa as bednets and anti-malarials become more widely available. However, transmission still persists and it appears that additional control measures are required. The leading malaria vaccine candidate in development is RTS,S, which has efficacy against clinical malaria measured at 30-50% in the field. This partial protection might be enhanced by combination with other components. The other vaccination approach that has produced repeatable efficacy in humans is the use of viral vectors to induce T cell responses. Previous attempts with this vaccine approach have been effective in challenge studies in Oxford, but ineffective in the field, probably because of reduced immunogenicity.

Recently, studies in Oxford have shown higher levels of immunogenicity by using a chimpanzee adenovirus (AdCh63) followed by an attenuated vaccinia virus (modified vaccinia Ankara) to deliver the pre-erythrocytic antigen, ME-TRAP. The increase in immunogenicity has lead to complete protection in 2 of 8 volunteers in challenge studies. Further challenge studies are in progress. It is important to measure immunogenicity in adults in Kenya at an early stage, to allow time to investigate and select the optimal regimen before planning for larger efficacy trials in the field.

We propose a Phase 1 study of safety and immunogenicity in a small group (n=30) of healthy adults in Kenya. We will assess safety by fieldworker and clinical evaluations post vaccination, and assess immunogenicity by the ELISpot assay and intra-cellular cytokine staining on FACS.

5) Introduction

Malaria transmission is now falling in some parts of Africa [1,2,3,4,5], leading some to suggest elimination is possible [6]. This is desirable but ambitious: there were 500 million malaria episodes worldwide in 2002, over a million deaths in Africa [7,8], and previous attempts at elimination have had mixed success [9,10,11]. It is currently thought that additional control measures will be required [12], including vaccination [13].

Progress towards a malaria vaccine

The candidate pre-erythrocytic malaria vaccine RTS,S, targeting the circumsporozoite protein, is the most advanced vaccine in development. Preliminary estimates of efficacy against infection rates after curative anti-malarial treatment were 34.0% (95%CI 8-53%) in adults [14] and 65.9% (95%CI 43-80) in infants [15]. Efficacy against the more clinically relevant endpoint of clinical malaria in 1-4 year old children was 29.9% (95%CI 11-45%)

[16]. Efficacy with the more immunogenic AS01 adjuvant was 52.9% (95%CI 28-69%) in Kenya and Tanzania [17]. A Phase III trial is now in progress. The observed efficacy of RTS,S is partial, indicating that we should continue to develop other candidate vaccines, which could be used in combination with RTS,S if efficacy is established, or give rise to more efficacious approaches. The only other vaccination approach that has demonstrated partial efficacy in humans is using virally vectored vaccines to induce T cells.

Previous studies with T cell inducing vaccinations

Following challenge studies in Oxford protecting 2 out of 16 volunteers using FP9 ME-TRAP followed by MVA ME-TRAP [18], studies were undertaken in adults and then children in Kilifi [19,20]. Immunogenicity was lower than expected [21], and efficacy was not seen in a study of 400 children in Kilifi district [22]. Furthermore, FP9 ME-TRAP shows variability in potency by batch [23]. Further development of T cell inducing vaccination in Oxford in pre-clinical studies has therefore examined more immunogenic vectors such as adenovirus vectors, in order to attain greater efficacy.

Pre-clinical studies

Using the chimpanzee adenovirus vectors AdCh6, AdCh7 and AdCh9 encoding ME-TRAP, and rendered non-replication competent by deletion of the virus E1 and E3 genes, in a P. berghei challenge model it has been shown that single immunisation resulted in sterile protection at sporozoite challenge in 67%, 83% and 92% of mice respectively [24]. In contrast, single dose MVA ME-TRAP and FP9 ME-TRAP alone offered no protection [25].
AdCh68 and AdCh63 expressing ME-TRAP generated similar construct specific T cell immunogenicity (measured by splenic peptide-specific interferon-γ (IFN-γ) secreting CD8+ T cells). However at challenge with P.berghei AdCh63 generated 83% protection at 20 days compared to 30% protection in the AdCh68 group (A. Reyes-Sandoval, unpublished). Protection was correlated with CD8+ IFNγ T cells by ICS.

Similar studies have compared the potency of the simian adenovirus vectors encoding ME-TRAP. AdCh9 produced the highest levels $CD8^+$ cells, and provided 92% protection to a P. berghei sporozoite challenge 14 days later compared to 83% protection with AdHu5 and AdCh7. At 60 days however protection to challenge was reduced to 17% in the AdCh9 group, 50% in the AdCh7 group and was absent in all other groups (Reyes Sandoval, submitted). A comparison of AdCh63 and AdCh9 expressing ME-TRAP showed a similar level of splenic IFN-γ secreting cells in mice, but AdCh63 produced high responses when measured in the blood (>20,000 SFC/million PBMCs compared to 12.5 million for AdCh9). After a challenge with P. berghei sporozoites, AdCh63 showed 83% protection at 20 days compared to 33% in the AdCh9 group (Reyes Sandoval, submitted).

Clinical studies of AdCh63 ME-TRAP in Oxford

64 healthy volunteers have received AdCh63 ME-TRAP in Oxford to date. Over 700 healthy volunteers and 5 HIV positive volunteers have received MVA ME-TRAP in Oxford, Kenya

and Gambia [18,19,22,26]. No linked serious adverse events were reported for either vaccine.

Experimentally induced malaria sporozoite infections ("sporozoite challenge") allow direct evaluation of a vaccine's efficacy prior to field studies. A phase 2a study began in February 2009 to determine the efficacy of AdCh63 ME-TRAP when used alone, and when used in combination with MVA ME-TRAP. AdCh63 was immunogenic, but did not protect any of 10 vaccinated volunteers. The AdCh63 ME-TRAP and MVA ME-TRAP regime was more immunogenic and protected 2 out of 8 volunteers. Protection in these volunteers was correlated with levels of CD8+ IFNγ T cells by ICS. All of 6 control unvaccinated volunteers developed malaria, as have all the 68 unvaccinated volunteers challenged to date in Oxford [27]. Further details of previous MVA ME-TRAP studies can be found in the IB for MVA ME-TRAP.

Investigational products relevant to this application.

The Modified vaccinia Ankara (MVA) vector

The MVA vector was selected for its safety and immunogenicity profile. Vaccinia was successfully used to vaccinate against and eliminate smallpox. MVA was originally derived from the vaccinia strain Ankara by over 500 serial passages in primary chicken embryo fibroblasts (CEF cells). MVA has six major genomic deletions compared to the parental Ankara genome and cannot replicate in non-transformed mammalian cells. The viral genome has been proven to be stable through a large series of passages in chicken embryo fibroblasts [28]. MVA shows no cytopathic effect or plaque formation in cells of human origin. In irradiated mice MVA did not elicit any morbidity or lethality even when administered at high doses intra-cerebrally [28]. From 1972 until 1980 (the end of compulsory smallpox vaccination) MVA was licensed in Germany [29] and was included in the official immunisation schedule [30]. In a large field study carried out in Germany over 120,000 previously unvaccinated individuals were vaccinated with MVA (0.2 mL) administered either intra-dermally or subcutaneously. The study population included high-risk groups (e.g. people suffering from allergies, elderly people, alcoholics) [28]. MVA proved to be non-contagious and avirulent. Viral replication is blocked late during infection of cells but importantly viral and recombinant protein synthesis is unimpaired even during this abortive infection. Replication-deficient recombinant MVA has been viewed as an exceptionally safe viral vector.

AdCh63 vector

Adenoviruses are attractive vectors for human vaccination. They possess a genetically stable virion so that inserts of foreign genes are not deleted. Adenoviruses can infect large numbers of cells without any evidence of insertional mutagenesis. Previous mass vaccination campaigns in very large numbers of US military personnel using orally administered live human adenovirus serotype 4 and 7 have shown good safety and efficacy data [31].

The most widely studied recombinant adenovirus vector is the human adenovirus, AdHu5. However, the ubiquity of human adenovirus infections can generate host anti vector immunity that may limit the utility of this vector. Depending on the geographical region, between 45 – 80 % of adults carry AdHu5-neutralising antibodies [32]. Immunisation with

AdHu vectors in animal models in the presence of pre-exposure to human adenoviruses attenuates responses to the vaccine [33,34,35]. Phase I trials of a multiclade HIV-1 vaccine delivered by a replication defective AdHu5 have previously excluded volunteers with pre-existing antibodies to AdHu5 at titres greater than 1:12 [36]. Higher antibody titres attenuate immunogenicity, although they do not result in higher reactogenicity [37].

The prevalence of immunity to human adenoviruses prompts the consideration of simian adenoviruses as vectors. They exhibit hexon structures homologous to that of human adenoviruses. Simian adenoviruses are not known to cause pathological illness in humans and the prevalence of antibodies to chimpanzee origin adenoviruses is less than 5% in humans residing in the US [38]. In a recent study in Kilifi Kenya, 23% of children (aged 1-6 years) had high-titre neutralising antibodies to AdHu5, whilst only 4% had high-titre neutralising antibodies to AdCh63 [39].

The ME-TRAP insert

The polypeptide encoded consists of a series of known CTL epitopes from Plasmodium falciparum pre-erythrocytic stage antigens [40], fused to a complete pre-erythrocytic stage antigen, Thrombospondin Related Adhesion Protein (TRAP) [41]. The individual CTL epitopes are recognised by a number of common human HLA types, represent a variety (six) of potentially protective target antigens and are included to ensure an immune response to the vaccine in the majority of the population vaccinated [42]. TRAP is an abundant pre-erythrocytic stage antigen. Human volunteers immunised with irradiated sporozoites and protected against malaria develop T cell responses against TRAP making it a strong candidate for inclusion in a malaria vaccine [43]. Viral vectors containing the CS antigen used in the RTS,S vaccines are much less immunogenic than TRAP, and were not protective in sporozoite challenge studies [44].

Laboratory assays

When assessing immunogenicity in clinical studies we use the gamma-interferon enzyme-linked immunospot (ELISPOT) assay in two forms. In its ex vivo form this assay correlated directly with protection in two mouse models of malaria [45]. In its short-term culture form, it correlated with protection in the field trial of RTS,S/AS02 in the Gambia [46] and in sporozoite challenge studies of viral vector vaccinations in Oxford [47]. We assay with pools of 20-mer peptides. The ELISPOT enumerates T cells in volunteers' peripheral blood which secrete gamma-interferon on contact with an epitope from the construct. Gamma-interferon secreted by T cells after interaction with infected liver cells has been shown to induce death of liver-stage parasites[48]. We will also use FACS studies to examine the CD8+ IFNγ T cell population that correlated with protection in the recent sporozoite challenge studies described above.

Study proposed for Phase 1 studies in Kilifi

The purpose of this trial is to assess the safety and immunogenicity of these promising candidate vaccines in healthy adult volunteers in a malaria endemic region. We would aim to progress to safety and then efficacy trials in Kenyan children under subsequent proposals. The regime proposed here has protected non-immune volunteers in Oxford against sporozoite challenge, and so may be protective against naturally acquired infection in Kenya.

The study population will comprise 30 healthy adult males aged 18-50. Although female volunteers have received these vaccines in Oxford, it is conventional for phase I studies of new vaccines in a new population occur first in adult males, to avoid the theoretical risk to foetuses which cannot be fully excluded by pregnancy testing and contraception during the course of the trial.

We will examine the safety and immunogenicity of the vaccination regimen found to be protective in Oxford (i.e. AdCh63 ME-TRAP followed by MVA ME-TRAP). MVA ME-TRAP has been extensively used, including trials in Kenya, and we will begin with the full dose. AdCh63 ME-TRAP has not been used before in Kenya, and so we will begin with one fifth of the dose used in Oxford in 10 volunteers before using the full dose. AdCh63 ME-TRAP has previously been delivered by intra-muscular injection, but MVA ME-TRAP has usually been given by intra-dermal injection. However, MVA can be delivered by the intra-muscular route [44], and this would be preferable for vaccinations in children. We will therefore allocate 15 volunteers to intra-dermal and 15 to intra-muscular injection, to establish immunogenicity and safety data for both routes of administration.

We do not propose to include a placebo group. At this stage our objective is to describe the safety profile in a small number of individuals, and the confidence intervals for the proportion of individuals with a particular event would be too wide for meaningful comparison with a placebo group. Immunogenicity will be judged by comparison with baseline.

Vaccine development plan

A correlate of immunity has been identified in the recent challenge studies, and this makes immunogenicity studies at Phase 1 particularly informative. In previous studies we found attenuated immunogenicity in Kilifi, which may have been due to exposure to malaria [21]. Phase 1 immunogenicity data from adults in Kenya would therefore have a substantial impact on the overall vaccine development plan, and it is important to assess this at an early stage, to allow time to investigate and select the optimal regimen before planning for larger efficacy trials in the field.

If the safety and immunogenicity data that we observe in the study relating to this application is favourable, as are the results of ongoing sporozoite challenge studies in Oxford and safety and immunogenicity data from adults and older children in The Gambia, then we would apply for permission for a Phase 2b trial in young children in 2011.

Potential subjects with HIV

Although it is standard practice to obtain safety data in healthy subjects before immunising those with HIV, we do not believe the vaccine to be unsafe in HIV infected individuals. The need for an HIV test before immunisation would preclude widespread delivery of a successful immunisation, and it is clearly desirable to protect HIV infected individuals as well as uninfected. Safety data for MVA is available from HIV infected adults in Germany [49] and New York [50], and for MVA ME-TRAP in adults in Kenya [19], and for 400 children in Kenya of whom 1% were probably HIV positive [22]. HIV is not reported as a risk factor for human adenoviral infection [51]. Furthermore, since AdCh63 is replication deficient in human cells, after initial infection of the cell that the virus enters there will be no

further viral replication, irrespective of immunodeficiency. However, in this first trial of AdCh63 ME-TRAP and MVA ME-TRAP in Africa, we will screen for and exclude HIV positive adults, but plan to include HIV positive volunteers at a later date.

6) Justification

This study is justified by the high incidence of malaria mortality and morbidity in Kenya and the rest of sub-Saharan Africa. There is pressing need for an effective vaccination. While insecticide-treated bednets, vector control measures and new cheap antimalarial drug development are all important aspects of malaria control, a co-existing vaccine development programme is essential.

Before efficacy trials can take place, it is necessary to identify a safe, immunogenic combination vaccine, and Phase 1 trials such as proposed here are essential for a rational vaccine development programme. The regime proposed here has protected non-immune volunteers in Oxford against heterologous sporozoite challenge, and so may be protective against naturally acquired infection in Kenya.

7) Null Hypotheses

This is a descriptive study to acquire safety data in a small group of volunteers. Null hypotheses are not applicable.

8) Objectives

General Objectives

To obtain preliminary data for use of AdCh63 ME-TRAP followed by MVA ME-TRAP in male adults in Kenya.

Specific objectives

To assess safety and reactogenicity of AdCh63 ME-TRAP followed by MVA ME-TRAP in adults in Kenya.

To evaluate the immunogenicity of AdCh63 ME-TRAP followed by MVA ME-TRAP in adults in Kenya.

To compare the use of intra-muscular and intra-dermal MVA ME-TRAP.

9) Design

Study site

The investigation will take place at the KEMRI Centre for Geographic Medicine Research – Coast, Kilifi. We will aim to recruit adults in local plantations, since these adults are less likely to leave the study area and this improves follow up.

Study population

There are well over 900 adult males between the ages of 18 and 50 years on the plantation. We will screen and recruit healthy adults in to the study, for a final sample size of 30.

Sensitisation and recruitment

We will hold a series of public meetings to explain the study. During a meeting the investigators will explain the following: the need for a vaccine; the current status of vaccine development; the study screening and informed consent procedure; risks of vaccination and the unproven benefits of vaccination. It will be stressed that this is an experimental vaccine, and cannot be guaranteed to provide protection and that it will therefore still be necessary to seek treatment for possible malaria even after vaccination. It will also be made clear that we will request HIV testing for volunteers. We will explain that we plan eventually to offer immunisation to those testing HIV positive, but that normal practice at is to begin with those who are HIV negative. In order to preserve the confidentiality of those volunteers infected with HIV who are undergoing immunisation, we will make it clear that screening on enrolment will include a range of diseases (not just HIV) and that only healthy adults may proceed to vaccination. Volunteers will have access to a trained counsellor. HIV positivity will be diagnosed by the standard rapid tests used in VCT centres. Results will be disclosed and post test counselling will be offered to all. We will offer a referral to CCRC at Kilifi District Hospital or any another appropriate health care for any testing positive.

After this meeting the field worker(s) will identify potential volunteers, and information sheets translated into relevant local languages will be distributed. During these discussions, it will be stressed that this is the beginning of a long process of vaccine development, which will be accelerated by the conduct of such small trials in Africa.

Field workers will visit each volunteer to explain the study further on an individual basis. Individuals who feel that the trial is appropriate for them will be invited to attend a formal screening visit. This will include a further discussion of the study with the principal investigator, and another opportunity for private discussion.

Screening visit.

We will take a clinical history, examine all volunteers carefully and conduct a number of standard laboratory tests (see below) to screen subjects for clinically significant acute or chronic diseases.

Inclusion criteria

- Consenting adult males aged 18-50 years in good health.
- Will remain resident in the study area for the study duration

Exclusion criteria

Exclusion criteria will be:

- Clinically significant history of the following conditions; skin disorder (eczema, etc.), allergy, symptomatic immunodeficiency, cardiovascular disease, respiratory disease, endocrine disorder, liver disease, renal disease, gastrointestinal disease, neurological illness.

- History of splenectomy
- Haemoglobin less than 9.0 g/dl
- Clinically significant abnormalities of laboratory screening tests (full blood count, ALT, creatinine levels, urine dipstick examination for blood and protein).
- Blood transfusion within one month of the beginning of the study
- History of vaccination with previous experimental malaria vaccines
- Administration of any other vaccine or immunoglobulin within two weeks before vaccination.
- Current participation in another clinical trial, or within 12 weeks of this study
- Any other finding which in the opinion of the investigators would increase the risk of an adverse outcome from participation in the trial.
- Likelihood of travel away from the study area
- HIV positive.
- History of contact dermatitis (due to the use of a potentially irritant disinfectant that may be present in trace amounts in the AdCh63 ME-TRAP vaccine, see the investigators brochure for details, attached)

Sample Size

The sample size for phase 1 studies balances the need to avoid exposing a large group to an unknown risk with the need for data on an adequate sample. The aim is not to provide pre-marketing safety data, but to justify the future study of larger groups. We plan to enrol 30 eligible male adults in this study.

Blood sampling

5 mls of blood will be collected at screening to test eligibility, which will be used for;

- Full blood count
- Serum ALT and creatinine
- HIV antibody testing (pre vaccination only)

40 mls of blood will be collected on weeks 1,3,9,10,14 and 45. The following tests will be performed at these time points;

- Full blood count
- Serum ALT and creatinine
- Cellular immunology studies.
- Plasma for antibody studies of immunity to viral vectors.

Laboratory procedure.

Plasma and cells will be stored at -20°C and -192°C respectively. *Ex vivo* ELISPOTs and FACS studies following intracellular cytokine staining are currently being performed in the immunology laboratory at the CGMRC, Kilifi, and results are validated by rigorous use of negative and positive controls. This technique uses an overnight stimulation by antigen of separated lymphocytes from the volunteer’s blood sample to count the number of interferon gamma producing cells. Analysis by FACS, where sufficient numbers of lymphocytes are

isolated, will allow further characterisation of the response, by co-staining lymphocytes with CD4 and CD8 as well as IFN gamma.

Vaccine administration

The vaccines will be shipped from Oxford on dry ice then stored at –70^{0}C at the CGMR-C unit until required. Vaccines will be thawed on the morning of use, and kept in a cold box in the field. They will be used within 4 hours of thawing.

Vaccination will be deferred in any volunteer with a clinical illness (defined as signs or symptoms of clear clinical disease and/or a fever >37.5°C) on the day of vaccination. Medical treatment will be provided, including inpatient care if necessary.

All AdCh63 ME-TRAP vaccinations will be intra-muscular, but MVA ME-TRAP vaccinations will be divided between intra-muscular and intra-dermal administration. Intradermal vaccinations will be given in the left deltoid region with an insulin syringe and needle (i.e. fine gauge). A randomization code list will be generated by an independent statistician and its use guided by a clear SOP on how the MVA ME-TRAP will be allocated to the two routes of vaccination. Intra-muscular vaccination will be given by a 23G needle and 1 ml syringe.

Each volunteer will be monitored for one hour (or longer if necessary) after each vaccination. Resuscitation (including intubation) equipment and medication will be available in the clinic site and a clinician trained in resuscitation present at all times.

We will use a dose escalation design, so that safety of a lower dose is evaluated before proceeding to the full dose. To ease operations, we will divide the cohort into 4 sub-groups with a week lag time between each as shown below.

Week			0	1	3	9	10	14	45
Group 1	A	3 Subjects	Screen •	AdCh63 ME-TRAP 1 x 10^{10}vp ♦	♦	MVA ME-TRAP 2 x 10^8 pfu ♦ *	♦	♦	♦
	B	7 Subjects	Screen •	AdCh63 ME-TRAP 1 x 10^{10}vp ♦	♦	MVA ME-TRAP 2 x 10^8 pfu ♦ *	♦	♦	♦
Group 2	A	10 Subjects	Screen •	AdCh63 ME-TRAP 5 x 10^{10}vp ♦	♦	MVA ME-TRAP 2 x 10^8 pfu ♦ *	♦	♦	♦
	B	10 Subjects	Screen •	AdCh63 ME-TRAP 5 x 10^{10}vp ♦	♦	MVA ME-TRAP 2 x 10^8 pfu ♦ *	♦	♦	♦

* The MVA ME-TRAP vaccination will be randomized 1:1 between intra-dermal and intra-muscular delivery. All the AdCh63 ME-TRAP doses will be administered intramuscularly.
Week 0 will not be the same date for the different groups. • = Blood test (5mls) and clinical examination. ♦=blood test (40mls) and clinical examination.
2 different vectors are used; AdCh63 = Chimpanzee Adenovirus 63, MVA= Modified Vaccinia Ankara.

Results of the FBC, creatinine and ALT will be reviewed before the next immunisation is given, and communicated to volunteers on their next clinic visit.

There will be a one week lag-time between the 4 sub-groups, so that any early adverse events from the low dose will inform the decision to immunise at a higher dose. Furthermore, in the low dose group (Group 1) we will begin by vaccinating 3 individuals only, so as to avoid exposing all 10 volunteers simultaneously.

Assessment and follow up

Medical officers and field workers supervised by medical officers **will assess and record local adverse events including pain, swelling, discolouration and limitation of arm movement according to the severity grading scales in Tables 2 and 3. He or she will then assess and record systemic adverse events including temperature, headache, malaise and nausea. Each side effect will be classified as absent, mild, moderate or severe according to the severity grading scale in Table 4. Each volunteer will be seen** ***at weeks 3, 10, 14 and 45 for a full safety and reactogenicity assessment by a medically qualified investigator*****. Additional visits at home will be made by a field worker (reporting to the medical officer) on days 1, 2 and 3 after each vaccination and if necessary daily, as directed by the medical officer, until the symptom(s) have resolved.** ***The medical officer will review reports from these home visits, and arrange to see the volunteers in person if appropriate.***

Volunteers will be asked to present to the clinic or to KEMRI if they develop any illness during follow up. Standard Operating Procedures, Case Report Forms and practice will be reviewed by an independent safety monitor. The emerging safety data will be described to the volunteers when they attend for the next immunisation.

The solicited adverse events that will be examined at each visit are shown in Table 1 below.

Table 1: Solicited local and general adverse events to be documented in the CRF

**Adverse events**	
**Local (injection site)**	Pain at the injection site
	Redness at the injection site
	Swelling at injection site
	Warmth at the injection site
	Itch at the injection site
	Scaling/Pustules/Blistering at injection site
**General**	Documented fever (axillary temperature > 37.5° C)
	Symptoms of fever
	Malaise
	Arthralgia
	Headache
	Myalgia
	Nausea / vomiting

Definitions of adverse event categories

Adverse Event

Any untoward medical occurrence in a patient or clinical investigation subject occurring in any phase of the clinical study whether or not considered related to the vaccine. This includes an exacerbation of pre-existing conditions or events, intercurrent illnesses, or vaccine or drug interaction. Anticipated day-to-day fluctuations of pre-existing conditions, including the disease under study, that do not represent a clinically significant exacerbation will not be considered adverse events. Discrete episodes of chronic conditions occurring during a study period will be reported as adverse events in order to assess changes in frequency or severity.

Adverse events will be documented in terms of a medical diagnosis(es). When this is not possible, the adverse event will be documented in terms of signs and symptoms observed by the investigator or reported by the subject at each study visit.

Pre-existing conditions or signs and/or symptoms (including any which are not recognised at study entry but are recognised during the study period) present in a subject prior to the start of the study will be recorded on the Medical History form within the subject's CRF.

Serious Adverse Events (SAE)

A serious adverse event is any untoward medical occurrence that at any dose:

- results in death,
- is life-threatening,

  Note: The term “life-threatening” in the definition of “serious” refers to an event in which the patient was at risk of death at the time of the event; it does not refer to an event which hypothetically might have caused death if it were more severe.

- requires inpatient hospitalisation or prolongation of existing hospitalisation,
- results in persistent or significant disability/incapacity, or
- is a congenital anomaly/birth defect.

Medical and scientific judgment should be exercised in deciding whether expedited reporting is appropriate in other situations, such as important medical events that may not be immediately life-threatening or result in death or hospitalisation but may jeopardise the patient or may require intervention to prevent one of the other outcomes listed in the definition above. These should also usually be considered serious.

SAEs are subject to expedited reporting to the sponsor, ethics committee and local safety monitor (see below).

Suspected Unexpected Serious Adverse Reactions (SUSAR)

An adverse reaction, the nature or severity of which is not anticipated based on the applicable product information (*e.g.*, Investigator's Brochure for an unapproved investigational medicinal product) is considered as an unexpected adverse drug reaction. Where the adverse reaction is also considered to have a possible, probable or definite relationship with the vaccine, and also meets the criteria for a serious adverse reaction, it is termed a Suspected Unexpected Serious Adverse Reaction (SUSAR). These events are subject to expedited reporting as for SAEs, and are also reported to the regulatory authority within one working day (see below).

Collection of Adverse Events

At each visit a physical examination will be documented including an examination of local reactions at the injection site. The largest diameter through the injection site of any redness will be recorded in millimetres. The largest diameter through the injection site of local swelling, defined as a more generalized swelling of the deltoid muscle will be recorded in millimetres. Severity of these local findings will be graded using the scale given in

Table 2.

Table 2: Grading a) for swelling

Grade	**Diameter [mm]**
0	0
1	< 20
2	20 – 50
3	> 50

b) for redness

Grade	**Diameter [mm]**
0	0
1	< 50
2	50 – 100
3	> 100

Study subjects will be asked to indicate the maximum degree of pain they experience at the injection site using a scale ranging from 0 to 3 as described in
Table 3

Table 3: Pain scale

Grade	Description
**0**	No pain at all
**1**	Painful on touch, no restriction in movement of arms, able to work, drive, carry heavy objects as normal
**2**	Painful when limb is moved<br>(*i.e.* restriction in range of movement in arm, difficulty in carrying objects)
**3**	Severe pain at rest<br>(*i.e.* unable to use arm due to pain.)

All local reactions will be considered causally related to the vaccination.

At each visit subjects will be requested to report local and general side effects they might have experienced since they last were seen. The investigator will assess the severity of the solicited signs and symptoms using the key provided in Table 4.

Table 4: Intensity of the general adverse events will be assessed as described:

GRADE 0	None
GRADE 1	Mild: Transient or mild discomfort (< 48 hours); no medical intervention/therapy required
GRADE 2	Moderate: Mild to moderate limitation in activity - some assistance may be needed; no or minimal medical intervention/therapy required
GRADE 3	Severe: Marked limitation in activity, some assistance usually required; medical intervention/therapy required, hospitalisation possible
GRADE 4	Serious, life-threatening: Extreme limitation in activity, significant assistance required; significant medical intervention/therapy required, hospitalisation or hospice care probable

The investigator will use the guidelines provided below to assess the relationship of the event to the administration of the vaccine. Both severity of the event and its relationship to the vaccine administration will be documented in the CRF.

Further details for any AE (such as start/stop date and any treatment), will also be gathered, regardless of the relationship to the vaccine. On the CRF space is allocated to document any unsolicited adverse event reported by the volunteer. Serious adverse events (SAE) as defined above will be documented in the CRF and reported using a serious adverse event reporting form.

Follow-up of Adverse Events

Adverse events likely to be related to the vaccine, whether serious or not, which persist at the end of the trial will be followed up by the investigator until their complete disappearance.

Moreover, any serious adverse event likely to be related to the vaccine and occurring after trial termination should be reported by the investigator according to the procedure described below.
Outcome of any non-serious adverse event occurring within 30 days post-vaccination (*i.e.* unsolicited adverse event) or any SAE reported during the entire study will be assessed as:

- Recovered/resolved
- Not recovered/not resolved
- Recovering/resolving
- Recovered with sequelae/resolved with sequelae
- Fatal (SAEs only)

Subjects who have moderate or severe on-going adverse events that are not vaccine linked will be referred to an appropriate at the completion of the study will be advised to consult a primary care physician if the event is not considered to be related to the study vaccine. A follow-up visit will be arranged to manage the problem and to determine the severity and duration of the event, if it is considered to be related to the study vaccine. If appropriate, specialist review within the Kilifi DGH will be arranged.

The causal relationship between the SAE and the product will first be evaluated by the investigator with the following scale:

No relationship:
No temporal relationship to study product; *and*
Alternate aetiology (clinical state, environmental or other interventions); *and*
Does not follow known pattern of response to study product

Possible relationship:
Reasonable temporal relationship to study product; *or*
Event not readily produced by clinical state, environmental or other interventions; *or*
Similar pattern of response to that seen with other vaccines

Probable relationship:
Reasonable temporal relationship to study product; *and*
Event not readily produced by clinical state, environment, or other interventions *or*
Known pattern of response seen with other vaccines

Definite relationship:
Reasonable temporal relationship to study product; *and*
Event not readily produced by clinical state, environment, or other interventions; *and*
Known pattern of response seen with other vaccines

Dissemination and explanation of blood results

All non-immunological blood results will be given to and explained to all participants after screening and at follow-up visits. Immunological results will be explained

in general terms, for the group not for individuals, at the end of the study. Those with abnormal blood results at screening will be offered appropriate investigations and treatment or referral as necessary.

Trial Governance

Oxford University will sponsor the study, and provide insurance for the trial. The study will be monitored by the Clinical Trials Facility in KEMRI, CGMR-C, and monitoring reports will be submitted to the sponsor at site initiation, following the first vaccinations, after the last vaccination, mid way during follow up, and at the study end. The monitor will carry out quality control of compliance with the protocol, selective quality control of data entry (with access to medical notes and CRFs) and check consent sheets. The monitor will submit a report to the sponsor via the clinical trials facility in KEMRI. Data management will be conducted in KEMRI, and a copy of the anonymized database provided to the sponsor for archiving.

The sponsor will convene a Data Safety Monitoring Board, that will review the safety data from low dose AdCh63 ME-TRAP (i.e. group 1) before high dose AdCh63 ME-TRAP (i.e. group 2) is used. A local safety monitor will be recruited by the investigators in KEMRI, whose role will be to communicate with the DSMB and provide independent assessments of unusual or serious adverse events should it be requested by the DSMB or by the investigators. SAEs will be reported (by the investigators) to the local safety monitor and to KEMRI ERC within one working day of the investigators becoming aware of them. The Principal Investigator will be responsible for reporting of SAEs to the DSMB in regular updates. The investigators will be responsible for reporting summary safety data at the end of the trial to the Kenyan Drugs and Poisons Board, and will report SUSARs and SAEs deemed causally related to the study vaccine to the Pharmacy and Poisons Board within one working day.

The DSMB will be empowered to request vaccinations be halted or discontinued, and will report to the sponsor. The investigators will inform KEMRI ERC and the Drugs and Poisons Board if the trial is discontinued, with a full explanation.

10) Data Management

Data storage

Adverse events will be documented in individual case report files for each volunteer. They will be recorded under two headings, local and general. There will be separate sections for concomitant medication, concomitant vaccination, non-serious adverse event documentation, serious adverse event documentation and study conclusion. Any deviations from the study protocol will be documented. Case report files will be kept securely, but may be taken to the field for direct entry of data. HIV status will not be recorded in the CRF, but will be held securely in a separate document by the principle investigator.

Data management and analysis

Data entry from paper records into a database will be conducted in the Clinical Trials Facility in KEMRI. For DSMB and end of trial reports, adverse events will be tabulated for summaries of frequency, duration and severity of event. Immunogenicity will be tabulated as geometric mean and confidence intervals of summed responses. This study is descriptive in nature, and data will be presented graphically for comparison. Formal statistical analysis is not planned.

The PI is able to confirm that "Identifiable subject information will be held securely, and accessed by the medical officer and other medical staff directly involved in the subject's clinical supervision only. Unidentifiable data will be made available to the other investigators for analysis."

11) Time Frame

Pilot Study

Not applicable

Definitive study

Community sensitisation, setting up of laboratory, test ELISPOTS, consent and screening procedures	1st Quarter 2010
Vaccinations	2nd Quarter 2010
Follow-up/data analysis	4th Quarter 2010

12) Ethical Considerations

Human subjects

Confidentiality

Personal information of the volunteers will be handled confidentially. All HIV tests and HIV related referrals will be handled with particular sensitivity. They will be linked to personal identifying information only on documents held securely in the Clinical Trials Facility, and no personal information will be entered into the electronic database. This will identify subjects by their unique code number only.

We can confirm that KEMRI IRB will have access to the data on site, and that KEMRI IRB may request summary or individual unidentifiable data for study subjects should there be any cause for concern, or an occasion for a "spot check".

Any future research related to the data or samples from this study will require written scientific and ERC approval before it can be done.

What risks may be involved?

The general risks to participants are those associated with blood sampling and vaccination. Mild tenderness, bruising, light-headedness, or rarely vasovagal syncope, may result from venepuncture. As with any invasive procedure, infection is also a risk.

This risk is minimized by the use of pre-packaged sterile equipment and careful attention to proper technique.
Potential risks from vaccination include local and systemic reactions, which shall be solicited for in this study. Local symptoms including discomfort/pain, redness, swelling, scaling, tenderness, itching or blistering at the site of injection may be experienced by the volunteers.

Vaccine related systemic serious adverse events are unlikely, but could rarely include a transient mild "flu-like" illness within 24 hours of vaccination which resolves rapidly. As with any vaccine, there is an extremely low chance of an anaphylactic reaction.

However, clinical experience with these vaccines is such that there have so far been no vaccine-related serious adverse events with either vaccine to date. Vacccination usually provokes a local inflammatory reaction, which may include redness, swelling, scaling, tenderness, itching or blistering. In previous studies using recombinant malaria vaccines, these local reactions have been mild to moderate and spontaneously resolved within days.

To date 85 volunteers have received AdCh63 ME-TRAP. Local adverse events such as pain would be expected to occur frequently. Less frequent adverse events are likely to include erythema, swelling, itching and warmth. Adverse events are likely to be mild in nature and should resolve rapidly. More than 700 volunteers have received MVA ME-TRAP in the UK, Kenya and the Gambia. Most volunteers developed erythema (or discolouration in African skin) and swelling post vaccination which in the majority of cases was mild to moderate in nature. This was accompanied by discomfort, warmth, itching and scaling which typically resolved to a faint flat papule. Intramuscular administration of MVA ME-TRAP has significantly less local reactogenicity than intradermal.

Duty to minimise risks to volunteers.

Adverse events are possible with any new vaccine. The risk is minimised by use of two candidate vectors chosen specifically for their good safety profile. Nevertheless, this study is designed to employ small numbers of closely supervised vaccinations, initially at reduced dose, to be sure of minimizing exposure should unexpected side effects occur. The comprehensive quality control, toxicology and manufacturing data and the absence of serious adverse events in phase I studies in Oxford provides evidence of the minimal nature of risk in this study. However anaphylaxis is estimated to occur at a frequency at 1 in 10^5 to 10^6 with all vaccines. Should such a rare event occur resuscitation facilities will be available to manage the reaction.

Benefits of participating in this study

There are no direct benefits to individuals participating in this phase I trial other than information about the volunteers' health status, and free medical care during the trial.

In terms of protection against malaria, this vaccine regimen has shown some evidence of protecting malaria naïve volunteers and therefore, may be protective against naturally occurring malaria infection in Kenya. However, because malaria mainly affects young children, the benefits of participating in this study may be considered as

being altruistic in nature as they would potentially benefit the wider society at large, if the vaccine is eventually proved to be safe and efficacious.

Duty to assure high standards of informed consent

We will provide detailed information about the study for distribution to volunteers. The principal investigator will endeavour to ensure that all volunteers fully understand the risks. Any participant who appears to have less than complete understanding will be excluded from enrolment by the principal investigator. As with any experimental vaccine the participants must understand that they have not yet been shown to prevent infection and this will be stressed during the recruitment stage. They must also understand the very small chance of anaphylactic reactions and thereby the importance of complying with the one hour observation period after each vaccination. The information sheet (attached) covers these points in detail, and each participant will have attended a public meeting, had the contents of the sheet explained in individual meetings on 2 separate occasions, including a quiz/ comprehension test administered prior to signing the form.

Compensation

We will also offer compensation for transport expenses, and time taken away from work in order to attend for immunisation and follow-up. The Social Behavioural Research group within the programme will lead the process of engagement with the community and shall consult widely before they reach a decision on what may be acceptable compensation per visit to the participants. That amount will take into consideration any lost wages for attending to the study while ensuring that it neither unduly coerces potential participants nor sets a difficult precedent for all research done within the programme. We believe that these benefits will not be excessive, and believe it would be unreasonable to request the cooperation of a population in regular employment without jeopardising their regular productivity or income.

Animal subjects

Not applicable

13) Expected Application of Results

Data from this study will provide further information about the safety and immunogenicity of these new promising vaccines, and such studies are essential for developing effective malaria vaccines. Findings would potentially benefit the wider community in allowing vaccine programmes for larger populations to progress.

14) References

1. Okiro EA, Hay SI, Gikandi PW, Sharif SK, Noor AM, et al. (2007) The decline in paediatric malaria admissions on the coast of Kenya. Malar J 6: 151.
2. Sievers AC, Lewey J, Musafiri P, Franke MF, Bucyibaruta BJ, et al. (2008) Reduced paediatric hospitalizations for malaria and febrile illness patterns following implementation of community-based malaria control programme in rural Rwanda. Malar J 7: 167.

3. O'Meara WP, Mwangi TW, Williams TN, McKenzie FE, Snow RW, et al. (2008) Relationship between exposure, clinical malaria, and age in an area of changing transmission intensity. Am J Trop Med Hyg 79: 185-191.
4. Ceesay SJ, Casals-Pascual C, Erskine J, Anya SE, Duah NO, et al. (2008) Changes in malaria indices between 1999 and 2007 in The Gambia: a retrospective analysis. Lancet 372: 1545-1554.
5. O'Meara WP, Bejon P, Mwangi TW, Okiro EA, Peshu N, et al. (2008) Effect of a fall in malaria transmission on morbidity and mortality in Kilifi, Kenya. Lancet 372: 1555-1562.
6. Greenwood BM, Fidock DA, Kyle DE, Kappe SH, Alonso PL, et al. (2008) Malaria: progress, perils, and prospects for eradication. J Clin Invest 118: 1266-1276.
7. Snow RW, Guerra CA, Noor AM, Myint HY, Hay SI (2005) The global distribution of clinical episodes of Plasmodium falciparum malaria. Nature 434: 214-217.
8. Snow RW, Craig M, Deichmann U, Marsh K (1999) Estimating mortality, morbidity and disability due to malaria among Africa's non-pregnant population. Bull World Health Organ 77: 624-640.
9. Stapleton DH (2004) Lessons of history? Anti-malaria strategies of the International Health Board and the Rockefeller Foundation from the 1920s to the era of DDT. Public Health Rep 119: 206-215.
10. Carter ED (2009) "God bless General Peron": DDT and the endgame of malaria eradication in Argentina in the 1940s. J Hist Med Allied Sci 64: 78-122.
11. Bruce-Chwatt LJ (1987) Malaria and its control: present situation and future prospects. Annu Rev Public Health 8: 75-110.
12. Feachem RG, Phillips AA, Targett G, Group TME (2009) Shrinking the malaria map. San Fransisco: The Global Health Group.
13. Penny MA, Maire N, Studer A, Schapira A, Smith TA (2008) What should vaccine developers ask? Simulation of the effectiveness of malaria vaccines. PLoS ONE 3: e3193.
14. Bojang KA, Milligan PJ, Pinder M, Vigneron L, Alloueche A, et al. (2001) Efficacy of RTS,S/AS02 malaria vaccine against Plasmodium falciparum infection in semi-immune adult men in The Gambia: a randomised trial. Lancet 358: 1927-1934.
15. Aponte JJ, Aide P, Renom M, Mandomando I, Bassat Q, et al. (2007) Safety of the RTS,S/AS02D candidate malaria vaccine in infants living in a highly endemic area of Mozambique: a double blind randomised controlled phase I/IIb trial. Lancet 370: 1543-1551.
16. Alonso PL, Sacarlal J, Aponte JJ, Leach A, Macete E, et al. (2004) Efficacy of the RTS,S/AS02A vaccine against Plasmodium falciparum infection and disease in young African children: randomised controlled trial. Lancet 364: 1411-1420.
17. Bejon P, Lusingu J, Olotu A, Leach A, Lievens M, et al. (2008) Efficacy of RTS,S/AS01E vaccine against malaria in children 5 to 17 months of age. N Engl J Med 359: 2521-2532.
18. Webster DP, Dunachie S, Vuola JM, Berthoud T, Keating S, et al. (2005) Enhanced T cell-mediated protection against malaria in human challenges by using the recombinant poxviruses FP9 and modified vaccinia virus Ankara. Proc Natl Acad Sci U S A 102: 4836-4841.
19. Bejon P, Peshu N, Gilbert SC, Lowe BS, Molyneux CS, et al. (2006) Safety profile of the viral vectors of attenuated fowlpox strain FP9 and modified vaccinia virus Ankara recombinant for either of 2 preerythrocytic malaria antigens, ME-TRAP or the circumsporozoite protein, in children and adults in Kenya. Clin Infect Dis 42: 1102-1110.
20. Bejon P, Mwacharo J, Kai OK, Todryk S, Keating S, et al. (2006) Immunogenicity of the candidate malaria vaccines FP9 and modified vaccinia virus Ankara encoding the pre-erythrocytic antigen ME-TRAP in 1-6 year old children in a malaria endemic area. Vaccine.
21. Bejon P, Mwacharo J, Kai O, Todryk S, Keating S, et al. (2007) The induction and persistence of T cell IFN-gamma responses after vaccination or natural exposure is suppressed by Plasmodium falciparum. J Immunol 179: 4193-4201.
22. Bejon P, Mwacharo J, Kai O, Mwangi T, Milligan P, et al. (2006) A Phase 2b Randomised Trial of the Candidate Malaria Vaccines FP9 ME-TRAP and MVA ME-TRAP among Children in Kenya. PLoS Clin Trials 1: e29.
23. Bejon P, Kai OK, Mwacharo J, Keating S, Lang T, et al. (2006) Alternating vector immunizations encoding pre-erythrocytic malaria antigens enhance memory responses in a malaria endemic area. Eur J Immunol 36: 2264-2272.
24. Reyes-Sandoval A, Sridhar S, Berthoud T, Moore AC, Harty JT, et al. (2008) Single-dose immunogenicity and protective efficacy of simian adenoviral vectors against Plasmodium berghei. Eur J Immunol 38: 732-741.
25. Sridhar S, Reyes-Sandoval A, Draper SJ, Moore AC, Gilbert SC, et al. (2008) Single-dose protection against Plasmodium berghei by a simian adenovirus vector using a human cytomegalovirus promoter containing intron A. J Virol 82: 3822-3833.

26. Moorthy VS, Imoukhuede EB, Milligan P, Bojang K, Keating S, et al. (2004) A Randomised, Double-Blind, Controlled Vaccine Efficacy Trial of DNA/MVA ME-TRAP Against Malaria Infection in Gambian Adults. Plos Med 1: e33.
27. Bejon P, Andrews L, Andersen RF, Dunachie S, Webster D, et al. (2005) Calculation of liver-to-blood inocula, parasite growth rates, and preerythrocytic vaccine efficacy, from serial quantitative polymerase chain reaction studies of volunteers challenged with malaria sporozoites. J Infect Dis 191: 619-626.
28. Mayr A, Stickl H, Muller HK, Danner K, Singer H (1978) [The smallpox vaccination strain MVA: marker, genetic structure, experience gained with the parenteral vaccination and behavior in organisms with a debilitated defence mechanism (author's transl)]. Zentralbl Bakteriol [B] 167: 375-390.
29. Mahnel H, Mayr A (1994) [Experiences with immunization against orthopox viruses of humans and animals using vaccine strain MVA]. Berl Munch Tierarztl Wochenschr 107: 253-256.
30. Stickl H, Hochstein-Mintzel V, Mayr A, Huber HC, Schafer H, et al. (1974) [MVA vaccination against smallpox: clinical tests with an attenuated live vaccinia virus strain (MVA) (author's transl)]. Dtsch Med Wochenschr 99: 2386-2392.
31. Top FH, Jr., Grossman RA, Bartelloni PJ, Segal HE, Dudding BA, et al. (1971) Immunization with live types 7 and 4 adenovirus vaccines. I. Safety, infectivity, antigenicity, and potency of adenovirus type 7 vaccine in humans. J Infect Dis 124: 148-154.
32. Tatsis N, Ertl HC (2004) Adenoviruses as vaccine vectors. Mol Ther 10: 616-629.
33. Kobinger GP, Feldmann H, Zhi Y, Schumer G, Gao G, et al. (2006) Chimpanzee adenovirus vaccine protects against Zaire Ebola virus. Virology 346: 394-401.
34. Casimiro DR, Chen L, Fu TM, Evans RK, Caulfield MJ, et al. (2003) Comparative immunogenicity in rhesus monkeys of DNA plasmid, recombinant vaccinia virus, and replication-defective adenovirus vectors expressing a human immunodeficiency virus type 1 gag gene. J Virol 77: 6305-6313.
35. Xiang Z, Gao G, Reyes-Sandoval A, Cohen CJ, Li Y, et al. (2002) Novel, chimpanzee serotype 68-based adenoviral vaccine carrier for induction of antibodies to a transgene product. J Virol 76: 2667-2675.
36. Catanzaro AT, Koup RA, Roederer M, Bailer RT, Enama ME, et al. (2006) Phase 1 safety and immunogenicity evaluation of a multiclade HIV-1 candidate vaccine delivered by a replication-defective recombinant adenovirus vector. J Infect Dis 194: 1638-1649.
37. Harro CD, Robertson MN, Lally MA, O'Neill LD, Edupuganti S, et al. (2009) Safety and immunogenicity of adenovirus-vectored near-consensus HIV type 1 clade B gag vaccines in healthy adults. AIDS Res Hum Retroviruses 25: 103-114.
38. Tatsis N, Blejer A, Lasaro MO, Hensley SE, Cun A, et al. (2007) A CD46-binding Chimpanzee Adenovirus Vector as a Vaccine Carrier. Mol Ther.
39. Dudareva M, Andrews L, Gilbert SC, Bejon P, Marsh K, et al. (2009) Prevalence of serum neutralizing antibodies against chimpanzee adenovirus 63 and human adenovirus 5 in Kenyan Children, in the context of vaccine vector efficacy. Vaccine 27: 3501-3504.
40. Gilbert SC, Plebanski M, Harris SJ, Allsopp CE, Thomas R, et al. (1997) A protein particle vaccine containing multiple malaria epitopes. Nat Biotechnol 15: 1280-1284.
41. Robson KJ, Hall JR, Jennings MW, Harris TJ, Marsh K, et al. (1988) A highly conserved amino-acid sequence in thrombospondin, properdin and in proteins from sporozoites and blood stages of a human malaria parasite. Nature 335: 79-82.
42. Lalvani A, Aidoo M, Allsopp CE, Plebanski M, Whittle HC, et al. (1994) An HLA-based approach to the design of a CTL-inducing vaccine against Plasmodium falciparum. Res Immunol 145: 461-468.
43. Rogers WO, Malik A, Mellouk S, Nakamura K, Rogers MD, et al. (1992) Characterization of Plasmodium falciparum sporozoite surface protein 2. Proc Natl Acad Sci U S A 89: 9176-9180.
44. Walther M, Thompson FM, Dunachie S, Keating S, Todryk S, et al. (2006) Safety, immunogenicity, and efficacy of prime-boost immunization with recombinant poxvirus FP9 and modified vaccinia virus Ankara encoding the full-length Plasmodium falciparum circumsporozoite protein. Infect Immun 74: 2706-2716.
45. Plebanski M, Gilbert SC, Schneider J, Hannan CM, Layton G, et al. (1998) Protection from Plasmodium berghei infection by priming and boosting T cells to a single class I-restricted epitope with recombinant carriers suitable for human use. Eur J Immunol 28: 4345-4355.
46. Reece WH, Pinder M, Gothard PK, Milligan P, Bojang K, et al. (2004) A CD4(+) T-cell immune response to a conserved epitope in the circumsporozoite protein correlates with protection from natural Plasmodium falciparum infection and disease. Nat Med 10: 406-410.
47. Keating SM, Bejon P, Berthoud T, Vuola JM, Todryk S, et al. (2005) Durable Human Memory T Cells Quantifiable by Cultured Enzyme-Linked Immunospot Assays Are Induced by Heterologous Prime Boost Immunization and Correlate with Protection against Malaria. J Immunol 175: 5675-5680.

48. Schofield L, Villaquiran J, Ferreira A, Schellekens H, Nussenzweig R, et al. (1987) Gamma interferon, CD8+ T cells and antibodies required for immunity to malaria sporozoites. Nature 330: 664-666.
49. Bavarian-Nordic (2002) Bavarian Nordic's HIV Vaccine Candidate Reveals Strong Saftey and Immunogenicity Profile in HIV Infected Patients. Copenhagen: Baverian-Nordic.
50. Xia Jin MR, Shady Barsoum, Geoffrey Deschenes, Lei Ba, James Binley, Daryl Schiller, Daniel bauer, David Ho, Martin Markowitz (2002) Saftey and Immunogencitiy of ALVAC vCP1452 and recombinant gp160 in Newly Human Immunodeficiency Virus Type 1 infected patients treated with prolonged Highly Active Antiretroviral Therapy. Journal of Virology 76: 2206-2216.
51. Gray GC, McCarthy T, Lebeck MG, Schnurr DP, Russell KL, et al. (2007) Genotype prevalence and risk factors for severe clinical adenovirus infection, United States 2004-2006. Clin Infect Dis 45: 1120-1131.

15) Budget

a) Personnel, salaries and benefits disbursement	US 100,000	Ksh	8,000,000
b) Patient costs, travel, food and/or supplies	US 1,000	Ksh	80,000
c) Equipment	US 50,000	Ksh	4,000,000
d) Supplies	US 20,000	Ksh	1,600,000
e) Travel and accommodation - local	US 700	Ksh	56,000
- international		Ksh	0
f) Transportation, vehicle repairs etc	US 20,000	Ksh	1,600,000
g) Operating expenses postage, printing etc.	US 1,000	Ksh	8,000
h) Animals acquisition etc.	US 0		
i) Consultancy fees	US 0		
j) Contingency fees (15% of above)	US 28,905	Ksh	2,312,400
k) Institutional administrative overheads	US 10,000	Ksh	800,000
Total	US 231,605	Ksh 18,528,400	

16) Justification of the Budget

As part of a series of studies on immunisations planned to take place, the costs are estimated as proportions of the overall expense. Personnel costs are to take account of the contributions by a medical officer, data manager (part time), one laboratory technician and 2 field workers over a 9 month period. Patient costs include compensation and transportation costs, as well as medical costs incurred in any treatment required. Equipment is estimated from costs recently incurred in immunological studies.

The study is part of the KEMRI-Wellcome Trust programme and will not incur any consultancy fees or additional administrative overheads.

17) Appendices

a) <u>State the role of each participating investigator</u>

Study design: Philip Bejon, Roma Chilengi, Adrian Hill, Kevin Marsh, Christopher Duncan.

Sensitisation and Recruitment: Roma Chilengi, Patricia Njuguna, Peninah Soipei, Vincent Mwatemo.

Clinical Laboratory: Ken Awoundo

Vaccine Management at Kilifi Site: Jimmy Shangala

Provision of vaccines: Adrian Hill, Alison Lawrie

Administration of vaccine, follow-up and collection of specimens: Roma Chilengi, Patricia Njuguna, Vincent Mwatemo, Peninah Soipei.

ELISPOT assays: Britta Urban

Data analysis: Roma Chilengi, Britta Urban, Philip Bejon

FACS data acquisition and analysis: Britta Urban

Project management in Oxford (regulatory, ethics submissions (Oxtrec) and Vaccine management: Alison Lawrie

Sponsors safety reporting and annual progress reports: Christopher Duncan

A medical officer will be recruited for the day to day running of the trial, who will be supervised by Roma Chilengi.

b) Please see attached documents: CV's for Non KEMRI investigators (Adrian Hill, Alison Lawrie, Christopher Duncan and Vincent Mwatemo), the Information Sheet/ Consent Form and Investigators Brochures.
